# Photoactivated Proximity Protein Labeling Reveals Enhanced Tumor Retention of a D‐Peptide‐Ruthenium Prodrug Conjugate

**DOI:** 10.1002/adhm.202502174

**Published:** 2025-10-20

**Authors:** Liyan Zhang, Peiyuan Wang, Hildert Bronkhorst, Yurii Husiev, Ludovic Bretin, Maarten N. van Ginkel, Wen Sun, Sylvestre Bonnet

**Affiliations:** ^1^ Leiden Institute of Chemistry Universiteit Leiden Einsteinweg 55 Leiden 2333 CC Netherlands; ^2^ State Key Laboratory of Fine Chemicals Dalian University of Technology 2 Linggong Road Dalian 116024 P. R. China; ^3^ Key Laboratory of Design and Assembly of Functional Nanostructures Fujian Institute of Research on the Structure of Matter Chinese Academy of Sciences Fuzhou 350002 P. R. China

**Keywords:** peptide chirality, photoactivated chemotherapy, proximity protein labeling, ruthenium‐peptide conjugates

## Abstract

Amino acid chirality is known to influence the biological properties of peptide‐containing prodrugs. In this work, both Δ and Λ isomers of three cyclic ruthenium‐peptide photoactivated chemotherapy (PACT) conjugates [**1**]Cl_2_‐[**3**]Cl_2_ are prepared that bear the bidentate peptide Ac‐MRGDM‐NH_2_, Ac‐mrGdm‐NH_2_, or Ac‐MrGdM‐NH_2_, respectively, where M, R, and D are l‐amino acids and m, r, and d are their D‐isomers. All six PACT compounds show low dark cytotoxicity (EC_50,dark_ > 30 µm) toward normoxic (21% O_2_) and hypoxic (1% O_2_) A549 human lung cancer cells. Upon green light irradiation, the peptide is cleaved off via an efficient two‐step photosubstitution reaction, which raises the cytotoxicity up to 20‐fold in normoxia and 4.5‐fold in hypoxia. The Λ‐[**1**]Cl_2_, Λ‐[**2**]Cl_2_ and Λ‐[**3**]Cl_2_ isomers are further studied in A549 human lung xenograft in mice. Strikingly, the D‐peptide conjugate Λ‐[**2**]Cl_2_ has higher antitumor activity than the two other isomers. For the first time, the fate of the photoactivated PACT prodrug can be tracked in vivo via red phosphorescence resulting from proximity labeling of histidine‐containing proteins. Photoactivated Λ‐[**2**]Cl_2_ shows higher tumor retention and better clearance from the rest of the body, thereby explaining the excellent antitumor properties of this PACT compound.

## Graphical abstract

Six stereoisomeric ruthenium‐peptide conjugates were synthesized and characterized, which showed high potential in photoactivated chemotherapy (PACT) and proximity protein labeling.

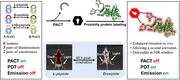

## 1 Introduction

When solid tumors reach a certain size too quickly, the O_2_ partial pressure in the tumor tissue may become very low due to sub‐optimal O_2_ delivery and high consumption of O_2_ caused by the high cell proliferation rates. This condition, which is called hypoxia, represents one of the most important barriers to the anticancer action of a number of chemotherapy agents.^[^ ^1^, ^2^, ^3^ ^]^ To adapt to the hypoxic environment of solid tumors, cancer cells trigger a biological survival response by switching on hypoxia‐inducible factors 1 and 2 (HIF1, HIF2).^[^ ^4^ ^]^ HIF activation reprograms cancer cells by regulating the expression of multiple genes involved in angiogejnesis, metabolic regulation, cancer cell invasion, metastasis, etc.^[^ ^3^ ^]^ Such reprogramming makes hypoxic solid tumors particularly resistant to many anticancer drugs, especially the ones that rely on the generation of reactive oxygen species (ROS).^[^ ^1^, ^5^ ^]^ For instance, cisplatin, one of the most commonly used chemotherapy drugs for treating lung cancer, not only generates DNA cross‐linking but also generates mitochondria‐dependent ROS that cause nuclear DNA damage and ultimately lead to cell death.^[^ ^6^, ^7^ ^]^ The clinical effectiveness of radiotherapy and immunotherapy is also directly or indirectly hindered by the hypoxic environment of the tumor. The particular concern goes to photodynamic therapy (PDT), in which a photosensitizer absorbs light and relies on either electron or energy transfer from the excited state of the photosensitizer to the O_2_ present in the irradiated tissues, to generate large amounts of cytotoxic ROS such as singlet oxygen (^1^O_2_), superoxide radical ions (O_2_^•−^) or hydroxyl radical (OH∙). At low O_2_ concentrations in tumor tissues, such generation usually fails, making PDT sensitizers lose their efficacy.^[^ ^8^, ^9^, ^10^, ^11^ ^]^

In order to overcome these limitations, an alternative light‐triggered cancer treatment modality called photoactivated chemotherapy (PACT) has been developed, which is characterized by its oxygen‐independent light activation mechanism.^[^ ^12^, ^13^ ^]^ PACT relies on a bond photocleavage reaction that releases a cytotoxic molecule that, in the dark, remains coordinated with the metal center.^[^ ^14^ ^]^ Depending on the molecular design of the prodrug, either the released ligand or the uncaged metal‐containing photoproduct may be cytotoxic.^[^ ^15^, ^16^, ^17^ ^]^ Many PACT compounds have been described,^[^ ^18^, ^19^ ^]^ but those based on ruthenium(II) polypyridyl complexes have emerged as particularly promising.^[^ ^20^, ^21^ ^]^ These complexes carry one or more photolabile ligands that are replaced by solvent molecules upon activation by visible light.^[^ ^19^, ^22^ ^]^ Different types of ligands have been described that are readily substituted, including ammines,^[^ ^23^ ^]^ nitriles,^[^ ^24^, ^25^ ^]^ thioethers,^[^ ^26^, ^27^ ^]^ or sulfonates.^[^ ^28^ ^]^ These ligands are usually much easier to photodissociate than pyridines or imidazoles because they generate an smaller energy gap between the ^3^MLCT and ^3^MC excited states of the ruthenium complexes.^[^ ^29^ ^]^

Usually, key factors proposed for the development of new PACT compounds are a suitable light activation window,^[^ ^30^ ^]^ high photosubstitution efficiency,^[^ ^27^ ^]^ and marked differences in cytotoxicity between dark and light conditions.^[^ ^22^ ^]^ When considering clinical applications, however, biosafety and bioavailability are equally critical: anticancer drugs should also have high solubility in aqueous solutions, good cellular uptake, good stability in biological media, and excellent tumor selectivity.^[^ ^31^ ^]^ To improve biocompatibility, conjugating biomolecules such as peptides^[^ ^32^, ^33^, ^34^, ^35^, ^36^, ^37^ ^]^ or proteins^[^ ^38^ ^]^ to the (pro)drugs has proven beneficial. Peptides are particularly attractivefor the development of targeted (pro)drugs, as they combine high selectivity with facile synthesis.^[^ ^39^, ^40^ ^]^ On the other hand, peptides can be hydrolyzed in vivo by peptidase enzymes present in the bloodstream, potentially reducing the selectivity, efficacy, and/or bioavailability of peptide‐drug conjugates. To increase the metabolic and circulation half‐life of peptides and peptide conjugates, different strategies have been described focusing on specific modifications at critical cleavage sites, including N‐/C‐terminal protection,^[^ ^41^ ^]^ peptide cyclization,^[^ ^42^ ^]^ the use of amide bond mimetics (e.g., thioamides, peptoids or β‐amino acids),^[^ ^43^, ^44^, ^45^ ^]^ or replacing natural l‐amino acids by unnatural d‐amino acids.^[^ ^46^ ^]^ In general, most natural proteins and peptides are composed of l‐amino acids; the complementarity between a peptide's chirality and that of its protein target is crucial for stereospecific binding and recognition. On the other hand, such complementarity also affects interactions of the peptide with targeted proteins on the cancer cell surface as well as its recognition by peptidase enzymes.^[^ ^47^ ^]^ Typically, higher physiological stabilities have been described for peptides that contain d‐amino acids, compared to their l‐peptide analogues.^[^ ^48^, ^49^ ^]^ When designing a drug‐peptide conjugate, it is hence essential to find a proper balance between d‐ and l‐amino acids to prevent as much as possible the digestion by peptidases while keeping a good binding to the protein target.^[^ ^50^ ^]^

We recently reported a study of integrin‐targeted ruthenium‐peptide conjugates for the PACT treatment of brain tumors.^[^ ^51^ ^]^ These compounds have the general formula [Ru(Ph_2_phen)_2_(Ac‐X_1_RGDX_2_‐NH_2_)]Cl_2_ (Ph_2_phen = 4,7‐diphenyl‐1,10‐phenanthroline). All compounds showed promising antitumor activity in vivo, but the mechanism of light activation was strongly dependent on the amino acids X_1_ and X_2_. When X_1_ and X_2_ were methionines, light activation consisted of a selective photosubstitution reaction breaking the Ru–S coordination bonds typical of PACT prodrugs, while when X_1_ and X_2_ were histidines, the molecule became red‐phosphorescent and killed cancer cells by a photodynamic mechanism based on O_2_ activation (PDT). In this work, we decided to explore the influence of the chirality of the amino acids of the methionine‐based PACT complex on their (photo)chemistry and (photo)biology. Three complexes [Ru(Ph_2_phen)_2_(peptide)]Cl_2_ complexes [**1**]Cl_2_‐[**3**]Cl_2_ were considered with three different peptides. In the first peptide, p1 = Ac‐MRGDM‐NH_2_, all amino acids had l‐chirality; in the second peptide, p2 = Ac‐mrGdm‐NH_2_, all amino acids had d‐chirality; and in the third peptide, p3 = Ac‐MrGdM‐NH_2_, a mixture of l‐ (methionine) and d‐ (arginine and aspartic acid) amino acids was used. In this notation, capital letters M, R, and D represent l‐amino acids, and small letters m, r, d are d‐amino acids. All isomers of these three cyclic Ru‐peptide conjugates were synthesized, isolated, and chemically characterized, and their photochemistry and photobiological properties in vitro and in vivo were evaluated in an integrin α_v_β_3_ overexpressing lung cancer tumor model. We serendipitously discovered that compound Λ‐[**2**]Cl_2_ had improved antitumor properties and improved clearance compared with the two other isomers tested in vivo, Λ‐[**1**]Cl_2_ and Λ‐[**3**]Cl_2_. More interestingly, we report for the first time that the fate of a photoactivated PACT prodrug can be tracked in vivo viared phosphorescence resulting from proximity labeling of histidine‐containing proteins by the ruthenium complex.

## 2 Results

### 2.1 Synthesis and Characterization

**Figure 1:**
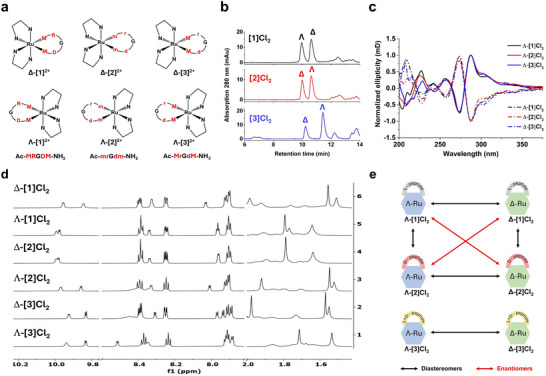
a) Simplified structures of Δ‐[**1**]^2+^, Λ‐[**1**]^2+^, Δ‐[**2**]^2+^, Λ‐[**2**]^2+^, Δ‐[**3**]^2+^ and Λ‐[**3**]^2+^ formed from different combination of Δ‐/Λ‐isomers on the octahedral ruthenium center, and the peptides Ac‐MRGDM‐NH_2_ (p1), Ac‐mrGdm‐NH_2_ (p2) or Ac‐MrgdM‐NH_2_ (p3). First column: Δ‐ and Λ‐isomers of [**1**]^2+^ carrying a fully L‐peptide. Second column: Δ‐ and Λ‐ isomers of [**2**]^2+^ carrying a fully D‐peptide. Third column: Δ‐ and Λ‐ isomers of [**3**]^2+^ carrying a mixed L/D‐peptide. Charges and terminal protecting group of the peptides are omitted for clarity.b) HPLC traces of the crude Ru‐peptide conjugates [**1**]Cl_2_, [**2**]Cl_2_, and [**3**]Cl_2_ l pep Λ‐/Δ‐peaks are attributed according to Figure S2 (Supporting Information). Gradient: 30–40% acetonitrile/H_2_O, 15 min, flow rate = 14 mL min^−1^, collection UV channel = 280 nm. c) Normalized CD spectra (0.1 mm, MilliQ H_2_O) and d) partial ^1^H NMR spectra (850 MHz, CD_3_OD) of HPLC‐isolated isomers Δ‐[**1**]Cl_2_, Λ‐[**1**]Cl_2_, Δ‐[**2**]Cl_2_, Λ‐[**2**]Cl_2_, Δ‐[**3**]Cl_2_ and Λ‐[**3**]Cl_2_. Full spectra can be found in the Supporting Information. e) Structural correlations between the six isomers.

The three ruthenium‐peptide conjugates [**1**]Cl_2_‐[**3**]Cl_2_ were prepared by mixing one of the three peptides p1, p2, or p3, with *rac‐*[Ru(Ph_2_phen)_2_Cl_2_] in a 1:1 mixture of ethanol and an aqueous solution at pH 7.0.^[^ ^51^ ^]^ As a result of the octahedral nature of the Ru center and its coordination with three bidentate chelating ligands, one of which was chiral, the Ru‐p1, Ru‐p2 and Ru‐p3 conjugates were obtained as diastereoisomeric mixtures: for each complex two diastereoisomers exist defined by the Δ or Λ configuration of the metal center combined with the l, d, or l/d‐ chirality of the peptide (Figure **1**a). All six diastereoisomers Δ‐[**1**]^2+^, Λ‐[**1**]^2+^, Δ‐[**2**]^2+^, Λ‐[**2**]^2+^, Δ‐[**3**]^2+^, and Λ‐[**3**]^2+^ were isolated as their chloride salts using high‐performance liquid chromatography (HPLC, Figure 1b). Different Δ/Λ diastereomeric ratios were obtained for the three Ru‐peptide conjugates, as shown in Figure 1a and Table S1 (Supporting Information). For [**1**]Cl_2_, the Λ‐isomer eluted first, and the ratio of isomers was 1:1 according to HPLC peak integrals and synthetic yields. For [**2**]Cl_2_ the ratio between the two isomers was also ≈1:1, but the Δ‐isomer eluted first from the HPLC. These results are logical: the similar fraction ratio of Δ/Λ isomers from [**1**]Cl_2_ and [**2**]Cl_2_ is consistent with an identical racemic *cis‐*[Ru(Ph_2_phen)_2_Cl_2_] precursor and mirror‐image peptides p1 and p2 (Figure 1e). For [**3**]Cl_2_, the Λ‐isomer eluted at a much later retention time, and a 1:2 ratio of Δ‐[**3**]Cl_2_ and Λ‐[**3**]Cl_2_ was observed, showing that changing the l‐Arg and l‐Asp residues in p1 to d‐analogues in p3 made the Λ configuration of the ruthenium complexes dominant (Table S1, Supporting Information).

Mass spectra (MS) analysis confirmed that the Ru:Ph_2_phen:peptide ratio was 1:2:1 without any other ligand involved, implying the formula of [Ru(Ph_2_phen)_2_(peptide)]^2+^ in all six diastereomers (Figure S3, Supporting Information). The 1D and 2D (COSY, NOESY and HSQC) NMR spectra revealed that upon coordination to ruthenium the thioether methyl peaks of the peptides (e.g., δ = 2.10 ppm in p2) shifted upfield (e.g., δ = 1.79 (N‐terminal)/1.64 (C‐terminal) ppm in Δ‐[**2**]Cl_2_) due to the shielding cone generated by the Ph_2_phen ligands (Figure S1 vs Figure S7, Supporting Information). This observation, together with the mass analysis, proved that the two methionine residues were successfully coordinated to the same ruthenium center, and hence that the conjugates were cyclic.

The chirality of the different isomers of the ruthenium‐peptide conjugates was unambiguously established by a combination of circular dichroism (CD) and proton nuclear magnetic resonance (^1^H NMR, Figure 1c,d). Before coordination to Ru, the CD spectra of the three diastereomeric peptides showed a main peak ≈228 nm with identical but either negative (p1) or positive (p2 and p3) ellipticity (Figure S2a, Supporting Information), originating from n→π* transitions in the amide bonds.^[^ ^52^ ^]^ p1 and p2 showed mirror CD spectra but identical ^1^H‐NMR spectra (Figure S1, Supporting Information) because they are enantiomers. For the CD spectra of the complexes [**1**]Cl_2_‐[**3**]Cl_2_ the higher‐energy UV region (between 200 and 230 nm) is a combination of the CD signal of the peptide itself and of the Ru polypyridyl center, thus the distinction of the spatial configuration of the peptides in [**1**]Cl_2_–[**3**]Cl_2_ was not feasible in this region (Figure S2a vs S2b–d, Supporting Information). However, the Δ/Λ configuration of the ruthenium center could clearly be established by the CD bands in the low‐energy UV region of the CD spectrum. Δ isomers of octahedral Ru(II) complexes are known to be characterized by a positive band at 270 nm and a deep negative ellipticity at 287 nm, while Λ isomers have opposite ellipticities at these wavelengths (Figure 1c).^[^ ^53^, ^54^, ^55^ ^]^ This analysis was confirmed by ^1^H NMR and HPLC retention times. Identical ^1^H NMR spectra were found for Δ‐[**1**]^2+^ and Λ‐[**2**]^2+^ on the one hand, and for Λ‐[**1**]^2+^ and Δ‐[**2**]^2+^ on the other hand (Figure 1d), proving that the reaction with enantiomer peptides p1 and p2 afforded, after HPLC separation, the expected pairs of enantiomers of the ruthenium‐peptide conjugates (Figure 1e). This enantiomeric relationship was confirmed by their identical retention times (Figure 1b; Table S1, Supporting Information). For pairs of diastereoisomers such as Δ‐[**1**]^2+^ and Λ‐[**1**]^2+^, the CD peaks were *almost* opposite due to the dominant ruthenium‐based transitions, but not exact due to the contribution of the peptide. The differences were also clear from their non‐identical NMR and retention times (Figure 1d). Overall, CD, NMR, and HPLC analysis afforded unambiguous structural characterization of the six isomers Δ‐[**1**]^2+^, Λ‐[**1**]^2+^, Δ‐[**2**]^2+^, Λ‐[**2**]^2+^, Δ‐[**3**]^2+^, and Λ‐[**3**]^2+^ as two pairs of enantiomers and five pairs of diastereomers.

### 2.2 Photochemistry Studies

**Figure 2:**
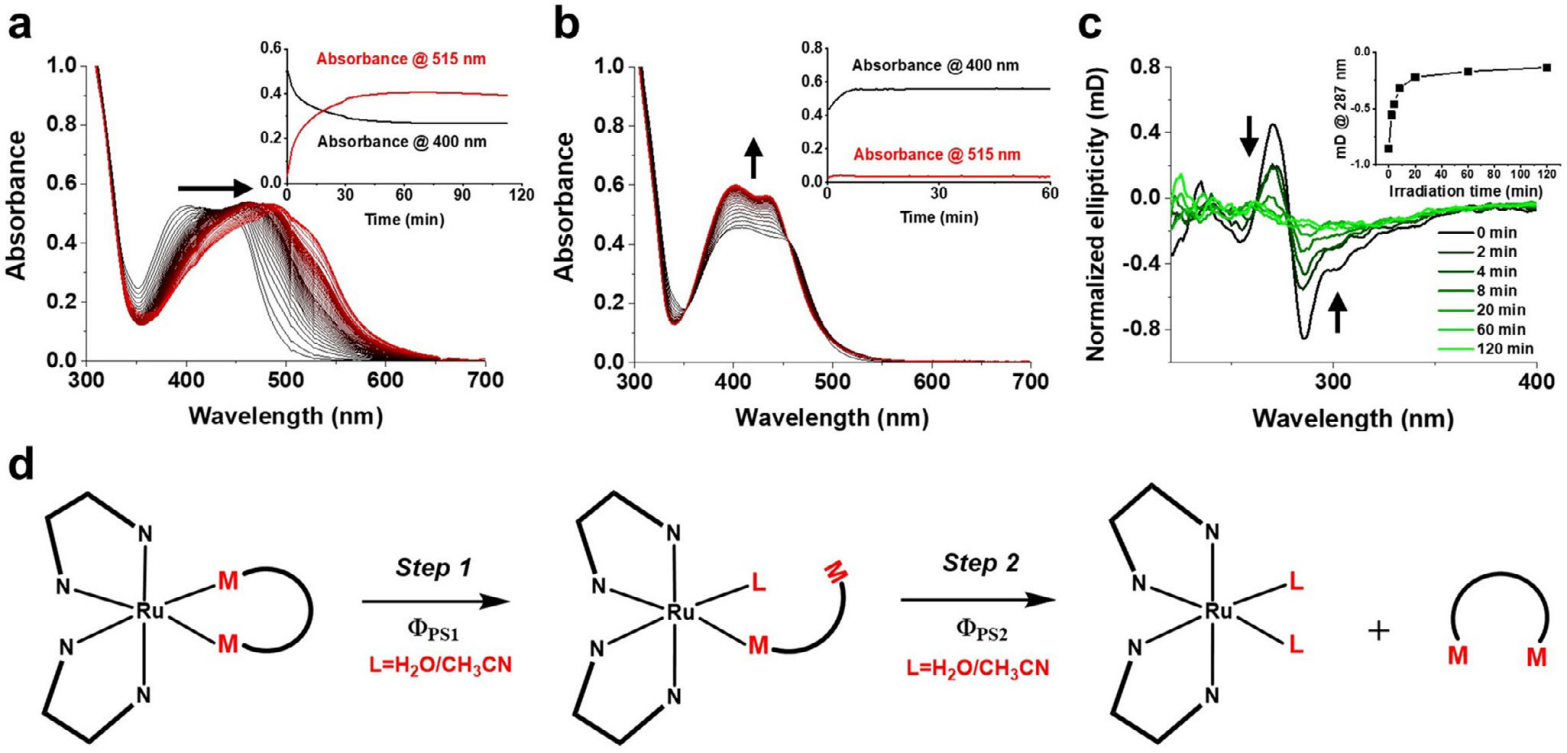
Time evolution of the absorbance spectrum of Δ‐[**1**]^2+^ (50 µm) in a) H_2_O and b) 1:1 v/v H_2_O:CH_3_CN during 120 min green light irradiation. Inset: time evolution of the absorbance at 400 or 515 nm versus irradiation time. c) Time evolution of the CD spectra of Δ‐[**1**]^2+^ (50 µm) in H_2_O under green light irradiation. Inset: normalized ellipticity at 287 nm versus irradiation time. d) Simplified scheme of the two‐step photosubstitution process when the Ru‐peptide conjugate was irradiated with green light in either H_2_O (ligand (L) = H_2_O) or H_2_O/MeCN mixture (ligand (L) = CH_3_CN); p1, p2, and p3 were simplified by M‐M. Light source: 515 nm light, 4 mW cm^−2^.

A prodrug candidate for PACT should be not only photoactive but also thermally stable in aqueous solution when kept in the dark, which can be monitored with UV–vis spectroscopy. The thermal stability of the six complexes was tested in MilliQ H_2_O and cell culture medium by monitoring their UV–vis spectra in the dark over time. No changes were observed for at least 60 h in water and 100 h in cell culture medium (see representative dataset for [**2**]Cl_2_ (1:1 mixture of Λ and Δ‐[**2**]Cl_2_) in Figure S15, Supporting Information), showing that the complexes are thermally stable in these conditions. The absorption spectra of the six compounds in water were comparable, as all six complexes showed a single broad singlet metal‐to‐ligand charge transfer (^1^MLCT) absorption band in the visible region between 400 and 500 nm, with an absorption maximum located ≈405 nm (Figure **2**; Figure S16, Supporting Information). The molar extinction coefficients (ε_max_ at 405 nm) of [**1**]Cl_2_–[**3**]Cl_2_ in water were also very similar (Table 2).

To determine the kinetics of light activation for the six isolated isomers, the time evolution of their absorbance spectra was monitored under green light activation (515 nm, 4 mW cm^−2^) in two solutions, i.e., H_2_O or 1:1 v/v H_2_O:MeCN. Representative spectra for the photosubstitution reaction of Δ‐[1]Cl_2_ are presented in Figure 2. The spectra for the other five isomers are shown in Figure S16 (Supporting Information). A redshift of the broad absorbance peak was observed upon irradiation of the compound in pure H_2_O. The mass spectra after irradiation showed the presence of the bis‐aqua photoproduct [Ru(Ph_2_phen)_2_(H_2_O)_2_]^2+^ (found 268.2, calcd *m/z* 267.7 for [M + H]^3+^, Figure S20a, Supporting Information). Irradiation of solutions containing acetonitrile resulted in an increase of the absorbance of the low‐energy ^1^MLCT band, with barely any shift of the absorption maximum, and a much faster photoreaction seemed to occur (inset in Figure 2b). In addition to monitoring the absorbance spectrum, the photosubstitution process was further monitored by CD spectroscopy. Using Δ‐[**1**]Cl_2_ as a representative example, continuous irradiation of its aqueous solution resulted in a rapid decrease in the ellipticity of the positive band at 270 nm and the negative band at 287 nm (Figure 2c), demonstrating that photoinduced isomerization of the chiral metal center was taking place in parallel to the ligand photosubstitution. Mass spectra after light irradiation in 1:1 v/v H_2_O:MeCN showed the photoproduct to be [Ru(Ph_2_phen)_2_(CH_3_CN)_2_]^2+^ (found 424.1, calcd *m/z* 424.1, Figure S20b, Supporting Information). The released free peptide Ac‐MRGDM‐NH_2_ was also detected by mass spectrometry with a peak at *m/z* = 650.4 (calcd. *m/z* 650.3 for [M + H]^+^) in both conditions. The five other diastereomers showed similar evolution of their absorbance and mass spectrometry spectra. When light irradiation was realized in MilliQ water containing 0.5 vol% DMSO, which corresponds to the maximum amount of DMSO that will be used in cytotoxicity studies in vitro, minor amounts of DMSO‐bound activated ruthenium complexes were also observed by HPLC/MS analysis (Figure S20c, Supporting Information). However, most of the activated molecules found by HPLC were Ru complexes bound to H_2_O or to MeCN from the solvent used in the HPLC system. These results suggested that the primary photoproduct in a water solution containing less than 0.5% DMSO was essentially bis‐aqua complexes, and that the DMSO‐bound photoproducts were minor impurities that did not significantly account for the biological properties of these compounds. Overall, all six isomers can be activated by green light irradiation to release the free peptide and the bis‐solvated ruthenium complex through photosubstitution.

Peptide photodissociation is a two‐step photoreaction, where the first methionine is substituted by one solvent molecule upon absorption of a first photon (quantum yield **Φ_PS1_**), after which the second methionine may be photosubstituted upon absorption of a second photon, usually with lower quantum yield **Φ_PS2_** (Figure 2d). In principle, good PACT compounds should have reasonable (>0.001) photosubstitution quantum yields. The quantum efficiencies **Φ_PS1_** and **Φ_PS2_** of the photosubstitution reaction in [**1**]Cl_2_–[**3**]Cl_2_ were quantified both in H_2_O and in H_2_O/MeCN mixtures (Table **1**; Figures S17–S19, Supporting Information). On average, the **Φ_PS1_** values were higher than the **Φ_PS2_** values in both conditions. Interestingly, however, **Φ_PS1_** in H_2_O was as high (>0.1) as the value in 1:1 v/v H_2_O:CH_3_CN. The fact that the first methionine dissociation was found similarly efficient in water, where a weakly coordinating aqua ligand comes in the first coordination sphere, and in water: acetonitrile mixtures, where the much better ligand CH_3_CN coordinates to ruthenium, suggested that these ruthenium‐peptide cyclic conjugates might be sterically strained. By contrast, for the second step, photosubstitution by CH_3_CN was found to be up to one order of magnitude more efficient than photosubstitution by H_2_O, which suggested that once the strain of the metallacycle has been released by the first photosubstitution step, the nature of the incoming ligand plays a more important role in the rate of the second photosubstitution step.^[^ ^56^, ^57^ ^]^ Comparable **Φ_PS1_** and **Φ_PS2_** values were found for the six diastereomers, although in the presence of MeCN, the two isomers of [**3**]Cl_2_ were found slightly more photolabile (Δ or Λ: **Φ_PS1_** = 0.20 or 0.21 and **Φ_PS2_** = 0.0067 or 0.0072, respectively).

**Table 1:** Photochemical properties of isolated Δ and Λ diastereoisomers of [**1**]Cl_2_–[**3**]Cl_2_ including molar extinction coefficients (ε, in m ^−1^ cm^−1^) at absorption maximum wavelength (405 nm), photosubstitution quantum yields for step 1 (Φ_PS1_) and step 2 (Φ_PS2_) in H_2_O or 50% MeCN in H_2_O under green light irradiation, and singlet oxygen quantum yields of each complexes in CD_3_OD.

Complex	*ε* × 10^4^ [m ^−1^ cm^−1^] at 405 nm^a)^	Φ_PS1_ in H_2_O ^b)^	Φ_PS2_ in H_2_O ^b)^	Φ_PS1_ in 1:1 H_2_O: CH_3_CN	Φ_PS2_ in 1:1 H_2_O: CH_3_CN	Φ_Δ_ (^1^O_2_)
Δ‐[**1**]Cl_2_	1.21	0.15	0.0059	0.17	0.043	0.008
Λ‐[**1**]Cl_2_	1.18	0.19	0.0057	0.14	0.030	0.009
Δ‐[**2**]Cl_2_	1.16	0.26	0.0052	0.11	0.032	0.009
Λ‐[**2**]Cl_2_	1.27	0.22	0.0050	0.17	0.057	0.014
Δ‐[**3**]Cl_2_	1.05	0.17	0.0092	0.21	0.067	0.011
Λ‐[**3**]Cl_2_	1.12	0.15	0.0073	0.20	0.072	0.005

^a)^ For ^1^O_2_ measurements [Ru(bpy)_3_]Cl_2_ was used as a reference compound, with Φ_Δ_ = 0.73 ± 0.12 in air‐saturated CD_3_OD under blue light irrqadiation (450 nm).^[^ ^58^ ^]^

^b)^ Light source: 515 nm (green light).

To evaluate the efficiency of these ruthenium‐peptide conjugates [**1**]Cl_2_–[**3**]Cl_2_ as potential PDT type II agents, the quantum yields (**Φ_Δ_)** of singlet oxygen generation were further measured by direct spectroscopic detection of ^1^O_2_ emission at 1275 nm in air‐saturated CD_3_OD (Table 1; Figure S21, Supporting Information). The **Φ_Δ_** value for a mixture of the Δ‐ and Λ‐ isomers of [**1**]Cl_2_ was already reported in our previous work (0.013 ± 0.005)^[^ ^51^ ^]^; within experimental errors, the values found for the isolated isomers were identical (0.008 ± 0.005 and 0.009 ± 0.005), and, like for the isomers of [**2**]Cl_2_ and [**3**]Cl_2_, very low (<0.015). Overall, all six isolated compounds are not likely to be good sensitizers for PDT type II. Upon green light activation, they open their metallacycle with high efficiency by photosubstitution of a first methionine ligand to release, after a slightly slower second photosubstitution step, the free peptide and (in aqueous solution) an activated bis‐aqua ruthenium complex. From this analysis, these compounds seem to be excellent prodrugs for PACT.

### 2.3 Integrin Expression and Cellular Uptake

**Figure 3:**
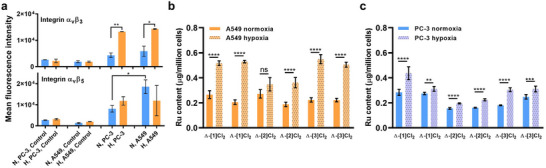
a) Expression of integrin a_V_β_3_ and a_V_β_5_ in A549 and PC‐3 human cancer cell lines under normoxic (21% O_2_, blue bars) or hypoxic (1% O_2_, orange bars) conditions. *Y*‐axis (N: normoxia, H: hypoxia) shows the fluorescence intensity of cells incubated with either anti‐integrin α_V_β_3_ or anti‐integrin α_V_β_5_ primary monoclonal antibodies, followed by secondary antibody conjugated to Alexa‐Fluor 488. Control indicates cells that were incubated with secondary antibody only and hence should not be emissive. Error bars show standard deviations (SD) from duplicate (*n* = 2) experiments. b) A549 and c) PC‐3 cells incubated with one of the six diastereomers of [**1**]Cl_2_–[**3**]Cl_2_ (10 µm, 24 h), as determined by ICP‐MS. Ru content (µg Ru per million cells) of normoxic and hypoxic cells. Error bars indicate the standard deviation from six experiments. Unpaired t‐test was used to determine the significance of the comparisons of data (**p < 0.01; ***p < 0.001; ****p < 0.0001).

Biologically speaking, the ruthenium‐peptide conjugates [**1**]Cl_2_–[**3**]Cl_2_ were designed to target specifically integrin proteins. For their biological study, two human cancer cell lines were chosen that were reported to have different integrin expression levels: A549 (human adenocarcinoma alveolar basal epithelial cells) and PC‐3 (human prostate cancer cells). The relative expression levels of the two integrin heterodimers α_v_β_3_ and α_v_β_5_ in both cell lines have been reported previously using a double‐immunofluorescence protocol.^[^ ^59^ ^]^ As discussed by our group,^[^ ^60^ ^]^ besides cell types, lower O_2_ concentrations might lead to activation of the HIF1A gene and consequently up‐regulation of the integrin expression.^[^ ^61^, ^62^, ^63^ ^]^ Thus, A549 and PC‐3 cells were cultured both in normoxic (21% O_2_) and hypoxic (1% O_2_) incubators, and their integrin expression levels were quantified by fluorescence‐activated cell sorting analysis (FACS, see Figure **3**a; raw histograms data Figure S22, Supporting Information). For integrin α_v_β_3_, the difference in expression between A549 and PC‐3 was quite limited; however, A549 was found to have significantly higher expression for α_v_β_5_ than PC‐3 in normoxic conditions. Interestingly, for PC‐3 and A549, higher expression of α_v_β_3_ was observed in hypoxia compared with normoxia, but the difference was not significant for α_v_β_5_. The observed up‐regulation of α_v_β_3_ in hypoxia could make this protein a possible target for the treatment of hypoxic regions of tumors with PACT compounds [**1**]Cl_2_–[**3**]Cl_2_.^[^ ^64^, ^65^ ^]^

To test these hypotheses, the cellular uptake of the different diastereomers in the two cell lines and two dioxygen concentrations was investigated. The amount of ruthenium in a cell can be measured with inductively coupled plasma mass spectrometry (ICP‐MS), as there is no ruthenium in a normal cell. A549 and PC‐3 cells in either normoxia or hypoxia were incubated for 24 h with the Δ or Λ isomers of [**1**]Cl_2_–[**3**]Cl_2_ (10 µm), then washed with ruthenium‐free medium, and finally digested with nitric acid before determining intracellular ruthenium concentrations with ICP‐MS. The uptake results quantified as µg Ru per million cells are shown in Figure 3b,c. The differences in uptake between A549 and PC‐3 cells, as well as between the six diastereomers, were quite limited in normoxia. However, the cellular uptake in hypoxic cells increased significantly for A549 for 11/12 groups that were studied, as well as for PC‐3 cells. These observations fit better with the relative expression levels of integrin α_v_β_3_ in normoxic and hypoxic cells than with the expression levels of α_v_β_5_. Overall, a higher uptake was found in hypoxic cells, which is consistent with the higher α_v_β_3_ expression observed in this cell line. Overall, from these data, it seems that these compounds target α_v_β_3_ better than α_v_β_5_.

### 2.4 Anticancer Efficacy in 2D Cell Monolayers and 3D Tumor Spheroids

Considering the potential of the six diastereomers for PACT, and their better targeting of α_v_β_3_ in hypoxic A549 cells, compared with PC‐3, their cytotoxicity was determined in 2D monolayers of normoxic and hypoxic A549 cells using a sulforhodamine B (SRB) cell quantification end point assay^[^ ^66^ ^]^ in the dark (light dose 0 J cm^−2^) or upon green light activation (light dose 13.1 J cm^−2^). Half‐maximal effective concentrations (EC_50_ in µm) were measured as an evaluation of the toxicity of the compounds. The photoindex values (PI), defined as EC_50,dark_/ EC_50,light_, were calculated to quantify the extent of light activation in cancer cells. The dose‐response curves and the corresponding EC_50_ values are shown in Figures S23–S26 (Supporting Information) and Table **2**, respectively. In a first approach, to prove that the Ru‐RGD conjugates were photoactivated, A549 cells were treated with purified Δ or Λ isomers of [**1**]Cl_2_–[**3**]Cl_2_ (24 h), and activated by light without refreshing the medium before light activation. In such a protocol, compounds that did not bind to the integrin target remain in solution after light activation, which maximizes their chances to be taken up and to kill the cells *after* light activation, i.e., irrespective of their integrin‐binding properties. EC_50,light_ values in normoxic A549 cells were between 1.9 and 3.0 µm for the six diastereomers, resulting in promising PI values between 11 and 17. In hypoxic A549 cells, the EC_50,light_ increased by a factor 2 (5.0–7.0 µm) compared with normoxia. However, as the EC_50,dark_ values became lower than those in normoxia, the PI values in hypoxia were reduced to 3.0–4.5, i.e., by a factor ≈4 compared to normoxia. The lower EC_50,dark_ values observed under hypoxia were unexpected; possibly this may be explained by the higher cellular uptake of the complexes (Figure 3b), resulting from the up‐regulation of integrins in hypoxia. On the other hand, increased integrin expression should also lead to increased toxicity *after* light activation, which we did not observe with our protocol. A hypothesis for this result may be that hypoxic cells are more resistant to the photoproduct of activation (i.e., [Ru(Ph_2_phen)_2_(H_2_O)_2_]^2+^) than to non‐activated conjugates. The cytotoxicity of [Ru(Ph_2_phen)_2_(H_2_O)_2_](PF_6_)_2_ in A549 normoxic cells was measured independently and found surprisingly low (EC_50_ > 100 µm, see Figure S27, Supporting Information). Probably, during incubation in cell media, the aqua ligands are replaced by biological ligands, which may either prevent the molecule from entering the cell or bring them to different cell compartments than those where the [**1**]^2+^–[**3**]^2+^ molecules are taken up. We believe that measuring the cytotoxicity of [Ru(Ph_2_phen)_2_(H_2_O)_2_]^2+^ by adding the compound outside the cells is not a good way to model the biological effects of the same molecule when it is generated inside the cell by light irradiation of [**1**]^2+^–[**3**]^2+^. Overall, this series of diastereomers showed promising PACT properties in both normoxic and hypoxic conditions, but the absence of a washing step before light activation made proper analysis of the in vitro cytotoxicity data confusing.

**Table 2:** Half‐maximal effective concentrations (EC_50_ in µM) with 95% confidence intervals (±CI in µm) and photoindexes (PI) for [**1**]Cl_2_–[**3**]Cl_2_ in the dark or upon green light irradiation, in 2D monolayers of A549 or PC‐3 cell lines and in 3D A549 tumor spheroids grown under normoxic conditions.

Cells^a)^, ^b)^	Condition		Light Dose J cm^−2^	Δ‐[1]Cl_2_			Λ‐[1]Cl_2_			Δ‐[2]Cl_2_			Λ‐[2]Cl_2_			Δ‐[3]Cl_2_			Λ‐[3]Cl_2_		
				EC_50_ [µm]	±CI	PI	EC_50_ [µm]	±CI	PI	EC_50_ [µm]	±CI	PI	EC_50_ [µm]	±CI	PI	EC_50_ [µm]	±CI	PI	EC_50_ [µm]	±CI	PI
A549 2D	Normoxia *no wash*		0	33	+17	**12**	49	+35	**16**	27	+9.9	**11**	31	+8.6	**12**	32^c)^	+13	**17**	49	+39	**16**
					−9.9			−10			−7.1			−6.3			−8.7			−10	
			13.1	2.7	+0.9		3.0	+0.8		2.4	+0.8		2.5	+0.84		1.9^c)^	+0.9		3.0	+1.2	
					−0.7			−0.7			−0.7			−0.71			−0.7			−0.97	
	Hypoxia *no wash*		0	21.1		**3.7**	18.3		**3.0**	23.6		**4.5**	29.4	+8.6	**4.4**	n.d. ^d)^			24.6		**4.0**
					−4.1			−3.9													
			13.1	5.7	+0.6		6.0	+0.9		5.3	+0.3		6.7	+0.7					6.2	+0.7	
					−0.5			−0.7			−0.3			−0.6						0.6	
	Normoxia *wash*		0	>50		**>3.8**	>50		**>6.3**	>50		**>6.9**	>50		**>5.1**	36	+16	**7.2**	21		**7.8**
																	−9.2			−2.3	
			13.1	13	+2.3		8.0	+2.8		7.2	+1.5		9.9	+2		5.0	+2.6		2.7	+0.4	
					−1.9			−2.2			−1.2			−1.6			−2.1			−0.4	
	Hypoxia *wash*		0	>50		**>1.6**	>50		**>1.3**	>50		**>3.1**	>50		**>1.9**	31	+3	**2.2**	25	+5.7	**3.3**
																	−2.9			−5.3	
			13.1	31	+6.9		34	+22		16	+3.2		27	+14		14	+3.7		7.5	+1.1	
					−6.3			−11.7			−2.7			−8.1						−1	
A549 3D		Normoxia *no wash*	0	>100	‐	**>5**	>100	‐	**>4**	>100	‐	**>3**	>100	‐	**>5**	n.d. ^d)^					
			13.1	17	4.5		23	4.8		27	5.9		18	3.4							
					−3.6			−3.9			−4.8			−2.9							

^a)^ PI = EC_50, dark_/EC_50, light_.

^b)^ Irradiation condition: normoxia 520 nm, 10.9 mW cm^−2^, 13.1 J cm^−2^, 20 min; hypoxia 520 nm, 7.22 mW cm^−2^, 13.1 J cm^−2^, 30 min.

^c)^ *n* = 2 instead of *n* = 3 due to limited compound stock.

^d)^ n.d. = not determined, Drugs [**3**]Cl_2_ were not included in this study due to insufficient reserves of these compounds. The medium was not refreshed to prevent the disintegration of the spheroids.

To solve this problem and better compare the targeting performance of the different diastereomers, a washing step was introduced just before light irradiation. This additional step should allow us to relate cell death to the uptake efficiency of compounds in the cells and to stronger binding to integrins before light irradiation. The cytotoxicity data obtained with this new protocol are shown in Table 2. Overall, the cytotoxicity with this new *wash* protocol was found to be lower, both before and after light activation, than in the *non‐wash* protocol due to the shorter contact between the cells and the prodrug in the *wash* protocol. Among the six diastereomers, differences between [**1**]Cl_2_, [**2**]Cl_2_, and [**3**]Cl_2_ were statistically non‐significant. It thus be concluded that the replacement of l‐amino acids with d‐amino acids did not decrease or increase the cytotoxicity of the ruthenium conjugates in this cell line.

A three‐dimensional (3D) tumor spheroids model (A549) under normoxia was further used to assess the efficacy of these ruthenium‐peptide conjugates, to better simulate the biological environment of physiological tumors (Table 2). Compared to 2D cell monolayers, 3D spheroids form a better in vitro model for the penetration of the prodrug, of light, and of dioxygen into the tumor.^[^ ^67^ ^]^ The treatment protocol was similar to the *non‐wash* protocol used in 2D, considering the necessity to avoid disturbing the physical structure of the spheroids. The EC_50_ values were determined by an ATP quantification end‐point assay called Cell Titer Glo 3D (Table 2; Figure S29, Supporting Information). In parallel, the morphology of the A549 spheroids was captured by bright‐field microscopy (Figure S28, Supporting Information). The EC_50,dark_ values were all higher than 100 µm, which is more than twice higher than those observed in normoxic A549 2D monolayers, highlighting the low toxicity of the ruthenium PACT prodrugs before light activation. Importantly, the high PI values observed in 2D were retained in 3D (PI>5). In fact, the EC_50,light_ values were found to be between 17 and 27 µM, which is 5–10 times higher than those measured in A549 monolayers. Possibly, the photoproduct [Ru(Ph_2_phen)_2_(H_2_O)_2_]^2+^ penetrated less easily into the 3D structure of the spheroids than in 2D. The spheroid size in our experiments was larger than usual to be closer to the tumor sizes in vivo (Figure S28, diameter of the non‐treated spheroids in this work on capture day ≈1400 µm vs 700 µm in previous work, Supporting Information),^[^ ^51^, ^68^ ^]^ which may contribute to the decreased EC_50_ values of the drugs after light activation. Spheroids with a diameter >400 µm can already develop a hypoxic core, in which the hypoxia‐signaling pathway is also activated, leading to possible resistance.^[^ ^69^ ^]^ Overall, the photoactivated toxicity of the six diastereomers in large 3D spheroids was promising (with PI up to >5), suggesting a high potential for PACT treatment in vivo without revealing important differences between the different isomers.

To determine the pathway by which the compounds cause cell death, a ROS generation assay was performed for Δ and Λ isomers of [1]Cl_2_–[3]Cl_2_ in A549 cells using the unspecific molecular probe CellROX Deep Red. This assay was used to quantify the formation of singlet oxygen, superoxide, and hydroxide radicals, both in the dark or upon green light irradiation (515 nm, 13.1 J cm^−2^). The cells treated with medium, *Tert*‐butyl hydroperoxide (tBHP), cisplatin, and Rose Bengal were used as vehicle control, positive control, negative control, and green light‐activated PDT type II control, respectively. These experiments were conducted in normoxic and hypoxic cells at the same time. The mean fluorescence intensity in each cell, which was quantified by FACS and normalized to that of controls, is a relative measure of intracellular ROS production (see Table **3**; raw data in Figures S30 and S31, Supporting Information). At the end of the assay, the absolute ratio R, defined as the ratio of the ROS probe emission intensity in the light group, divided by that in the dark group, was used as quantification of light‐induced ROS production.

**Table 3:** Normalized intracellular ROS generation in A549 cells according to FACS analysis using CellROX Deep Red Reagent as probe, after treatment with Δ‐ or Λ‐[**1**]Cl_2_–[**3**]Cl_2_, cisplatin, Rose Bengal (15 µm, 24 h), in the dark and after light irradiation (515 nm, 13.1 J cm^−2^).

Complex^a^ ^),^ ^b^ ^)^	Normoxia (21%)			Hypoxia (1%)		
	dark	Light	R	Dark	Light	R
tBHP	14.00			4.36		
Vehicle control	1.00	2.06	2.06	1.00	1.55	1.55
Δ‐[**1**]Cl_2_	0.63	3.88	6.18	0.78	1.04	1.32
Λ‐[**1**]Cl_2_	0.57	2.61	4.58	1.03	1.70	1.65
Δ‐[**2**]Cl_2_	1.10	4.80	4.37	1.12	1.18	1.04
Λ‐[**2**]Cl_2_	1.24	2.20	1.77	1.22	1.83	1.50
Δ‐[**3**]Cl_2_	1.01	2.97	2.95	1.07	1.92	1.78
Λ‐[**3**]Cl_2_	1.05	3.55	3.39	0.91	1.99	2.20
Cisplatin	1.28	2.23	1.74	1.00	1.99	2.00
Rose Bengal	0.90	4.71	5.21	0.84	2.56	3.05

^a)^ ROS amounts were quantified by normalized mean fluorescence intensity (Figures S30 and S31, Supporting Information) to dark control (normoxia: 18 194; hypoxia: 16 637) in corresponding groups.

^b)^ tBHP (250 µm, 1 h) was used as a positive control for oxidative radical production.

When cells were cultured with any of the six ruthenium complexes and left in the dark, ROS generation was not observed (R = 0.57–1.24, comparable with vehicle control). When light was applied in the presence of one of the ruthenium complexes, R values higher than 1 were observed in normoxia (R = 1.8–6.2); some of the compounds generated as much ROS as the PDT agent Rose Bengal (R = 5.2), which is counter‐intuitive considering the low **Φ_Δ_** value of singlet oxygen generation found for all complexes. Significant variations in ROS production were observed, however, for the different isomers, from R = 1.8 with Λ‐[**2**]Cl_2_, which is identical to the control with light only, to R = 6.2 for its enantiomer Δ‐[**1**]Cl_2_, which was even higher than with Rose Bengal. In hypoxia, the R values after light activation were found to be low for all ruthenium complexes (R = 1.0–2.2), and comparable to that of the negative control cisplatin. Thus, no significant ROS generation occurred with or without light activation at 1% O_2_. Considering the phototoxicity observed in hypoxia in vitro, we conclude that phototoxicity for this series of Ru‐peptide conjugates in hypoxia is not based on the generation of ROS, while in normoxia, ROS generation plays a significant role in their phototoxicity.

**Figure 4:**
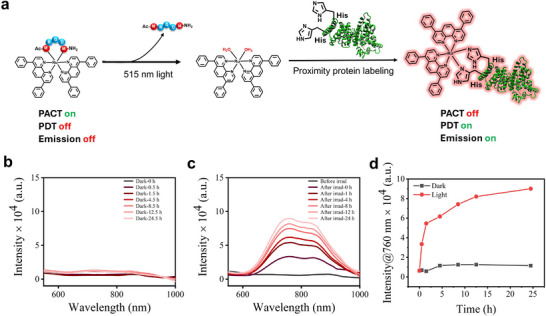
a) Schematic illustration of the protein binding behaviors of the series complexes after light irradiation. b) Emission intensities of Λ‐[**1**]Cl_2_ (λ_ex_ = 480 nm, 10 µm) in the presence of bovine serum albumin (BSA, 50 µm) under dark conditions in PBS. c) Emission intensities of Λ‐[**1**]Cl_2_ (λ_ex_ = 480 nm, 10 µm) in the presence of BSA (50 µm) before and after light irradiation (515 nm, 10 mW cm^−2^, 30 min). Emission spectra were collected before irradiation and *t* = 0, 1, 4, 8, 12, and 24 h after irradiation. d) Emission intensity at 760 nm versus time plotted from b and c.

To explore the counter‐intuitive, O_2_‐dependent ROS generation in normoxia in a series of complexes that do not generate significant amounts of ^1^O_2_, we decided to explore protein binding in the presence of bovine serum albumin (BSA) following light irradiation. We previously suggested that the bis‐aqua photosubstitution product [Ru(Ph_2_Phen)_2_(H_2_O)_2_]^2+^ may be able to interact with histidine residues in endogenous proteins, to form ruthenium‐histidine secondary photoproducts capable of producing ROS (Figure **4**a).^[^ ^51^ ^]^ To check this hypothesis, one of the complexes (e.g., Λ‐[**1**]Cl_2_) was mixed with an excess of BSA (5 fold) in a PBS buffer solution, and left in the dark or irradiated by green light. Here, BSA was considered as a prototypical histidine donor as each molecule of BSA contains 17 histidine residues. As shown in Figure 4b–d, when this mixture was kept in the dark, no significant red emission was observed up to 24 h after preparing the sample. By contrast, when the solution was irradiated with green light (515 nm, 10 mW cm^−2^, 30 min), red emission between 600 and 1000 nm was quickly detected, suggesting the formation of secondary photoproducts between the activated PACT ruthenium complex and the BSA protein. Histidine is a classical biometal‐binding residue, and previous work has reported that iridium and ruthenium complexes may undergo ligand exchange specifically with histidine as well, to generate red phosphorescence.^[^ ^70^, ^71^, ^72^ ^]^ Our experiment clearly proved that such a reaction took place only following light activation, to generate an emissive secondary photoadduct between [Ru(Ph_2_Phen)_2_(H_2_O)_2_]^2+^ and histidine‐containing proteins such as BSA, which might be observable in vivo.

### 2.5 Antitumor Study In Vivo

**Figure 5:**
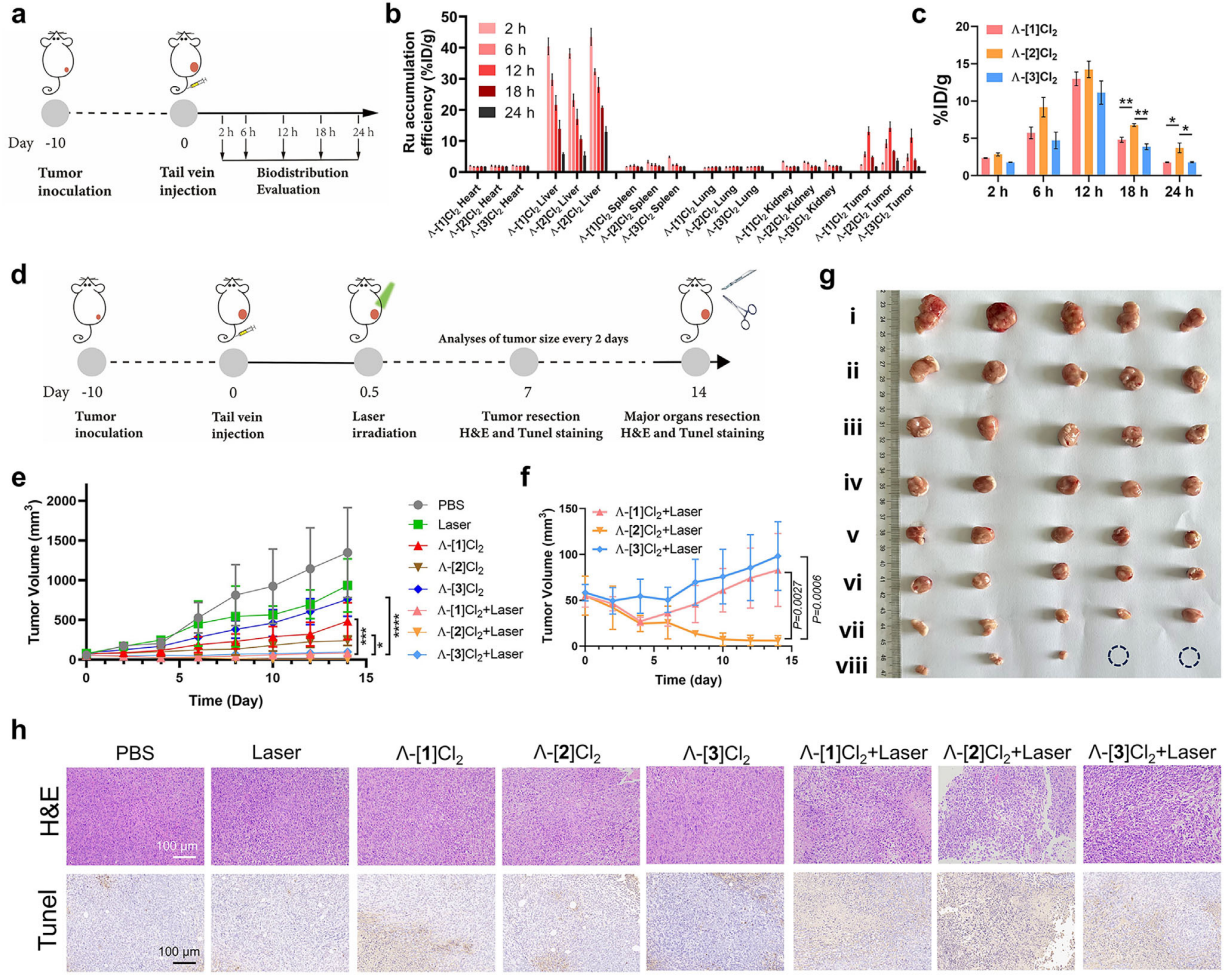
Antitumor efficacy in vivo. a) Scheme of the schedule of the biodistribution study. b,c) Biodistribution of Ru delivery efficacy (% ID g^−1^, *n* = 3) in major organs (b) and in the tumor (c) at different time points following intravenous injection of Λ‐[**1**]Cl_2_, Λ‐[**2**]Cl_2_ or Λ‐[**3**]Cl_2_ (7.7 mg kg^−1^) in A549 tumor‐bearing BALB/c nude mice. % ID g^−1^ = Ru content (mg) /tissue (g) compared to total injection of Ru (µg) determined by ICP‐OES. Errors represented standard error (SE) over three independent experiments (*n* = 3). d) Scheme of the schedule of the antitumor efficacy study. e) Time evolution for 14 days of the tumor volume following treatments using: PBS (i), Laser (ii, 520 nm, light dose = 60 J cm^−2^), Λ‐[**3**]Cl_2_ (iii), Λ‐[**1**]Cl_2_ (iv), Λ‐[**2**]Cl_2_ (v), Λ‐[**3**]Cl_2_ + Laser (vi), Λ‐[**1**]Cl_2_ + Laser (vii), Λ‐[**2**]Cl_2_ +Laser (viii). The last three groups: Λ‐[**1**]Cl_2_ + Laser, Λ‐[**2**]Cl_2_ + Laser, Λ‐[**3**]Cl_2_ +Laser were zoomed in f). Errors represented the standard deviation (SD) over five independent experiments (*n* = 5). g) Photographs of the tumor captured on day 14. h) H&E and TUNEL‐stained images of tumor slices after treatment with PBS, Laser, Λ‐[**1**]Cl_2_, Λ‐[**2**]Cl_2_, Λ‐[**3**]Cl_2_, Λ‐[**1**]Cl_2_ + Laser, Λ‐[**2**]Cl_2_ + Laser, and Λ‐[**3**]Cl_2_ + Laser on day 7. Two‐way ANOVA was used to determine the significance of the comparisons of data (*p < 0.05; **p < 0.01; ***p < 0.001; ****p < 0.0001) in this figure.

As discussed in the introduction, the presence of d‐amino acids in peptide conjugates such as [**2**]Cl_2_ or [**3**]Cl_2_ may result in different stability and target binding in physiological conditions. Since in vitro experiments cannot fully mimick the influence of proteases and the complex microenvironment of solid tumors, an A549 xenograft tumor mouse model was used to study the biodistribution and antitumor efficacy of the series of complexes [**1**]Cl_2_‐[**3**]Cl_2_ in vivo. Considering the massive work necessary to study six different complexes in both dark and light conditions and the minimum difference observed in vitro between the Λ and Δ isomers (Tables 2, 3), only the Λ‐isomers of [**1**]Cl_2_, [**2**]Cl_2_, and [**3**]Cl_2_ were included in the following in vivo study. A biodistribution study was first conducted to evaluate the influence of peptide chirality on the accumulation of the conjugates in A549 tumor‐bearing mice. Identical molar amounts of Λ‐[**1**]Cl_2_, Λ‐[**2**]Cl_2_, or Λ‐[**3**]Cl_2_ (7.7 mg kg^−1^) were injected intravenously in the tail of subcutaneous tumor‐bearing nude mice on day 10 after implantation of the A549 cells. Inductively coupled plasma optical emission spectroscopy (ICP‐OES) was then used to quantitatively evaluate the concentration of Ru at five different time points (2, 6, 12, 18, and 24 h) in different organs and the resected tumor (Figure **5**a). The relative drug delivery efficiency was then calculated in percent injected drug per gram tissue (%ID g^−1^). The liver was the major organ for microphase Ru uptake, and the three compounds showed similar hepatic pharmacokinetics within the first 24 h post‐injection (Figure 5b). Maximum accumulation of the compounds in the tumor was found at 12 h post‐injection (> 10% ID g^−1^), suggesting that t = 12 h is the ideal Drug‐to‐Light Interval (DLI) time point for these compounds (Figure 5c). Overall, all three isomers behaved similarly up to 12 h, but at longer times (18, 24 h) post‐injection Λ‐[**2**]Cl_2_ was retained better in the tumor area than Λ‐[**1**]Cl_2_ and Λ‐[**3**]Cl_2_, highlighting the influence of peptide chirality on tumor retention.

To further test how differences in chirality may influence antitumor efficacy, an antitumor efficacy study of these three compounds in a subcutaneous A549 tumor‐bearing Balb/c mouse model was performed. All mice were randomly divided into eight groups (*n* = 5) and received various treatments named PBS, Laser, Λ‐[**1**]Cl_2_, Λ‐[**2**]Cl_2_, Λ‐[**3**]Cl_2_, Λ‐[**1**]Cl_2_ + Laser, Λ‐[**2**]Cl_2_ + Laser, or Λ‐[**3**]Cl_2_ +Laser. Compound injection in the tail vein was performed once (7.7 mg kg^−1^) at day 0 and light activation after a DLI of 12 h was performed using a green laser (520 nm, light dose = 60 J cm^−2^). The tumor volume and body weight of each mouse were recorded every 2 days for 14 days, after which the mice were sacrificed for histological analysis of the major organs (Figure S33, Supporting Information). During these 14 days, the body weights of the mice barely changed, highlighting the low toxicity of these compounds even following light activation (Figure S32, Supporting Information). According to the time evolution of the tumor volumes (Figure 5e), all three compounds were found to have better antitumor performance in the light group than in the non‐irradiated group. The *p* value comparison between the tumor volume of the Λ‐[**1**]Cl_2_ versus Λ‐[**1**]Cl_2_ + Laser, Λ‐[**2**]Cl_2_ versus Λ‐[**2**]Cl_2_ + Laser, and Λ‐[**3**]Cl_2_ versus Λ‐[**3**]Cl_2_ + Laser groups at day 14, calculated by 2 way‐ANOVA, were <0.0001, 0.0079, and <0.0001, respectively. A TUNEL staining assay, realized on day 7, demonstrated that all three complexes induced outstanding cell death when combined with laser irradiation (Figure 5h). Most interestingly, among all the laser groups, Λ‐[**2**]Cl_2_ showed better antitumor performance than [**1**]Cl_2_ and [**3**]Cl_2_ specifically after day 7. Though the tumors in the Λ‐[**1**]Cl_2_ +Laser and Λ‐[**3**]Cl_2_ +Laser groups slowly grew from ≈50 to ≈100 mm^3^ over 14 days, for the Λ‐[**2**]Cl_2_ +Laser group, the average tumor volume was almost zero at the same time point, and 2 mice were even cured! At this stage, the difference in efficacy between Λ‐[**2**]Cl_2_ on the one hand and Λ‐[**1**]Cl_2_ and Λ‐[**3**]Cl_2_ on the other hand is unclear. In particular, no significant difference in intra‐tumoral Ru concentration was observed at the time of irradiation (12 h post‐injection) for Λ‐[**1**]Cl_2_, Λ‐[**2**]Cl_2_ and Λ‐[**3**]Cl_2_, However, from the biodistribution study two different observation can be mentioned: first, Λ‐[**2**]Cl_2_ was retained longer in the tumor than the other two complexes (Figure 5c), and hence it has more time to exert its antitumor efficacy. Second, the group treated with Λ‐[**2**]Cl_2_ but non‐irradiated with a laser showed a lower tumor growth in the dark (*p* value<0.0001) compared with the groups treated with Λ‐[**1**]Cl_2_ or Λ‐[**3**]Cl_2_. Overall, whether the d‐peptide‐containing conjugate Λ‐[**2**]Cl_2_ shows a higher stability toward endogenous peptidases or binds better to the integrin target than the analogues with l‐amino acids or a mixture of l‐ and d‐amino acid residues, the former was more efficiently retained in the tumor than the latter, and it showed exquisite antitumor efficacy.

**Figure 6:**
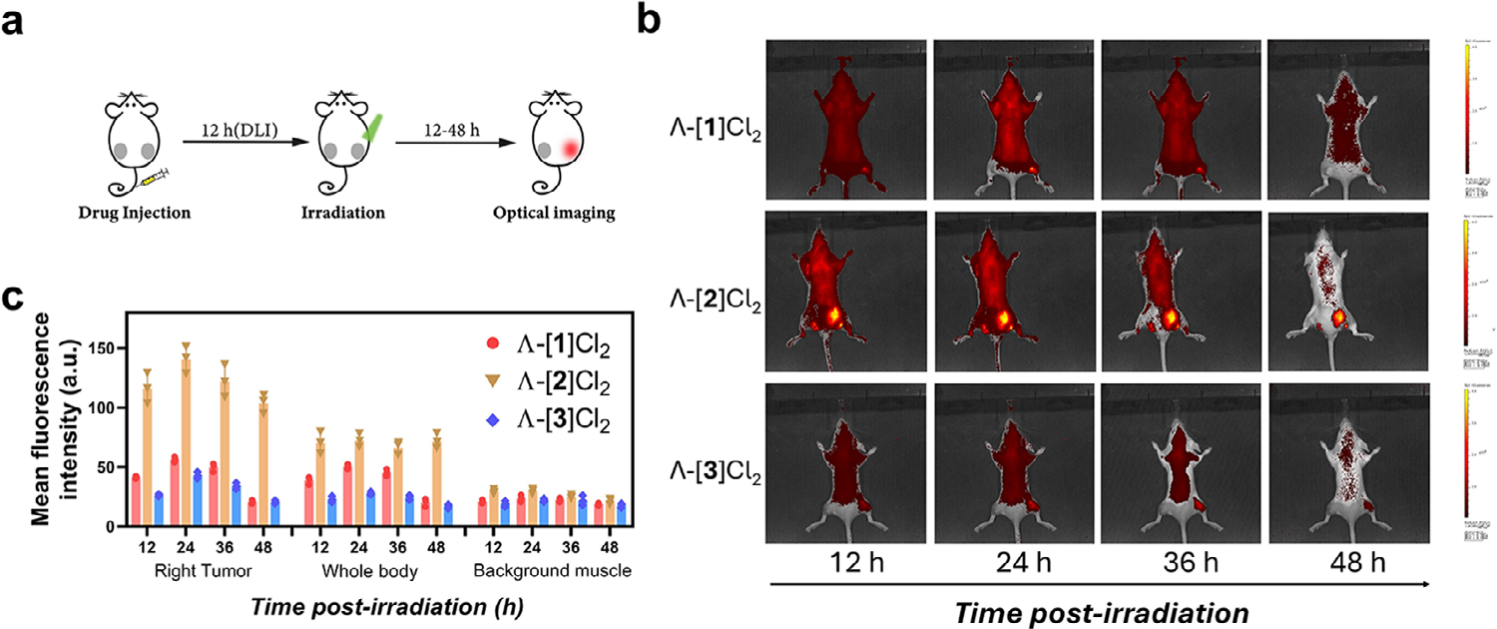
Optical fluorescence imaging of the post‐irradiated mice in vivo. a) Scheme of the simplified schedule of the imaging process. b) Optical images (Ex = 488 nm, 600 nm long‐pass filter) of the A549 tumor‐bearing BALB/c nude mice at different times (12, 24, 36, and 48 h) after injection with Λ‐[**1**]Cl_2_, Λ‐[**2**]Cl_2_ or Λ‐[**3**]Cl_2_ (7.7 mg kg^−1^) following irradiation (right tumor) or not (left tumor). 12 h post‐injection green light irradiation was conducted (520 nm, 100 mW cm^−2^, twice 5 min with a 5 min interval, total light dose 60 J cm^−2^) to activate the PACT prodrug. c) Mean fluorescence intensity of different parts of the mice corresponding to (b), errors represented standard deviation (SD) over three independent mice (*n* = 3).

As the in vitro BSA‐binding experiments confirmed the formation of red‐emissive secondary photoproducts, optical fluorescence images of the treated mice were recorded at 12, 24, 36, and 48 h after green laser irradiation. In this new study, A549 cells were implanted on both sides of the hind limbs, and after 10 days and following injection with Λ‐[**1**]Cl_2_, Λ‐[**2**]Cl_2_ or Λ‐[**3**]Cl_2_, the right limb of the tumor was irradiated with green laser (520 nm, light dose = 60 J cm^−2^), while the left side of each mouse was kept in the dark (Figure **6**a). As shown in Figure 6b, red emissive signals were clearly detected in the irradiated tumor area (λ_ex_ = 488 nm, 600 nm long‐pass filter) for all groups, and the non‐irradiated sides were barely emissive, with a similar intensity as the background. Among all three groups, Λ‐[**2**]Cl_2_ showed the highest red emission intensities both in the right tumor (the one that had been irradiated) and in the whole body (Figure 6c). To our knowledge, these are the first images of a ruthenium‐based light‐activated PACT prodrug in vivo after irradiation treatments. Even 48 h post‐irradiation, clear red emission was still detectable in the irradiated tumor, revealing the enhanced retention of ruthenium, while residual emission in the rest of the body was clearly decreasing. However, red emission in the tumor was much stronger for Λ‐[**2**]Cl_2_ than for Λ‐[**1**]Cl_2_ and Λ‐[**3**]Cl_2_, while residual emission in the rest of the body was stronger for Λ‐[**1**]Cl_2_ and almost absent for Λ‐[**2**]Cl_2_ and Λ‐[**3**]Cl_2_. These results confirmed the better Ru retention in the tumor measured by ICP‐OES for Λ‐[**2**]Cl_2_, and suggested that improved tumor retention might contribute to the better antitumor properties of Λ‐[**2**]Cl_2_ in this compound series. Our current hypothesis is that photoactivated peptide disassociation releases [Ru(Ph_2_phen)_2_(L)_2_]^2+^ species with L = OH_2_, Cl^−^, or other coordinating small biomolecules or ions, that upon reaction with nearby histidine‐containing proteins such as serum albumin, generate a red‐emissive bis‐histidine photoproduct [Ru(Ph_2_phen)_2_(His)_2_]^2+^. This secondary photoproduct is both a PDT‐active molecule capable of generating ROS upon further light irradiation and cytotoxic in the dark. This hypothesis is corroborated both by the experimental results shown in Figures 4 and 5 and by our recent report on bis‐histidine cyclic ruthenium‐peptide analogue [Ru(Ph_2_phen)_2_(HRGDH)]^2+^ that was shown to have good photodynamic properties.^[^ ^51^ ^]^ It does remain unclear, however, how better tumor retention and better clearance from the rest of the body can be linked to the reversed d‐chirality of the mrGdm peptide in the prodrug conjugate Λ‐[**2**]Cl_2_. Indeed, after light activation, the pentapeptide is supposed to be cleaved off from the ruthenium center, and peptides with different chirality should not influence the properties of the Ru‐containing product of light activation. On the other hand, different peptide chirality may influence how the prodrug localizes before light activation, which, after light activation, may lead to different biological properties for the three isomers. Another hypothesis is that in the conditions of our in vivo experiment, only a fraction of the molecules fully lose the pentapeptide to produce the red‐emissive photoproduct, while a second fraction of semi‐activated molecules bound to only one of the methionine residues of the peptide remains. For this second fraction of semi‐cleaved photoproduct, which may contribute to the observed antitumor properties of the activated drug, chiral information is still present.

## 3 Conclusion

In this study, six isomers of the [Ru(Ph_2_phen)_2_(**pi**)]^2+^ conjugates (i = 1, 2, 3) were synthesized, isolated, and characterized, where **p1**, **p2**, and **p3** are bis‐methionine MRGDM pentapeptides with different chirality on the side chain carbon atom. A combination of physical, chemical, and biological studies suggested that the compounds behave primarily as typical PACT prodrugs, characterized by high photosubstitution quantum efficiencies, poor ^1^O_2_ generation properties, and photoindex values that remain high in hypoxic A549 lung cancer cells. Once activated by light in living cells, all compounds were capable of releasing the free peptide. The resulting primary ruthenium photoproduct could subsequently coordinate with nearby proteins, leading to the formation of secondary photoproducts probably bound to two histidine ligands. These secondary products are photodynamically active and may explain part of the observed phototoxicity, but they may also contribute to cell death by light‐independent antitumor properties in vivo. In vitro, the use of d‐amino acids in the peptide chain did not result in a significant change in the PACT efficiency of the Ru‐peptide conjugates. In vivo, however, striking differences in tumor retention and antitumor properties were observed for the different PACT compounds bearing peptides with different chirality. Following intravenous injection of Λ‐[**1**]Cl_2_, Λ‐[**2**]Cl_2_ and Λ‐[**3**]Cl_2_ into a mice lung tumor model (A549), Λ‐[**2**]Cl_2_ was found to have the highest accumulation in the tumor after 12 h post‐injection, and also the best antitumor efficacy in the irradiated groups, even curing two of the tumor‐bearing mice. Interestingly, light activation led to the formation of a secondary red‐emissive Ru‐based photoproduct, allowing for following in situ monitoring of the activated prodrug in the tumor and in the mouse body. Such red‐emissive species are the result of the binding of the Ru‐based photoproduct to histidine‐containing proteins such as albumin. It is the first time one can see a light‐activated ruthenium‐based PACT product in vivo. Histidine labeling is a technology considered significantly important for the construction of protein‐based biomaterials and the analysis of protein sequencing.^[^ ^73^ ^]^ We are convinced that the surprising observation reported here may offer new perspectives for using PACT ruthenium complexes such as [**1**]Cl_2_, [**2**]Cl_2,_ or [**3**]Cl_2_ as photoactivated proximity protein labeling reagents in the future. Overall, this work offers promising perspectives for the development of ruthenium complexes for PACT treatment of tumors, and a clear demonstration that differences in peptide chirality can lead to different tumor retention and antitumor activities in vivo.

## 4 Experimental Section

### General

The photochemistry studies by UV–vis, and integrin expression were carried out according to the methods described in the previous work.^[^ ^51^ ^]^

### Compounds Preparation

The peptides were purchased from Biomatik or ChinaPeptides and applied in synthesis without any further purification. *cis*‐[Ru(Ph_2_phen)_2_Cl_2_] was synthesized according to existing procedures.^[^ ^16^ ^]^ In general, RuCl_3_∙3H_2_O (196 mg, 0.75 mmol, 1 eq), 4,7‐diphenyl‐1,10‐phenanthroline (500 mg, 1.5 mmol, 2 eq), LiCl (952 mg, 22.5 mmol, 30 eq) and hydroquinone (215 mg, 1.95 mmol, 2.6 eq) were added to a dried 50 mL round bottom flask first, vacuum and N_2_ were alternately used to degas flask by three times, then 10 mL degassed deoxygenate dry DMF was injected into flask. The mixture solution was then refluxed for 8 h under N_2_. After the reaction was completed, the flask was then cooled to room temperature and then poured into 50 mL acetone, and placed in the freezer overnight (−30 °C). The solution was then filtered and washed with cold H_2_O and diethyl ether three times. A dark purple powder was obtained after drying under vacuum (350 mg, yield 56%). The precursor was then used directly in the following Ru‐peptide conjugate synthesis.

The general synthesis procedure of Ru‐peptide conjugates was as follows: *cis*‐[Ru(Ph_2_phen)_2_Cl_2_] (0.10 mmol, 83.6 mg, 1 eq) was added to a 50 mL two‐neck flask and purged with N_2_ three times. The peptide p1, p2, or p3 (0.10 mmol, 65.0 mg, 1 eq) was dissolved in water (8 mL) and the pH adjusted to 7 by adding 1 m NaOH. After adding deoxygenated ethanol (8 mL) to the reaction flask, the peptide aqueous solution was deoxygenated by N_2_ bubbling for 10 min and then injected into the reaction flask. The reaction mixture was then refluxed for 7 days at 80 °C under N_2_. Then, ethanol was rotary evaporated, and the remaining aqueous mixture was filtered (RC 60 Membrane Filters, cytiva) under vacuum and washed with MilliQ water (≈20 mL × 3 times). The combined filtrate was freeze‐dried and stored (50–60 mg) until purification by high‐performance liquid chromatography (HPLC).

The HPLC purification was realized on a 250 × 21.2 mm Jupiter 4 µm Proteo 90 Å C12 column using the Thermo Scientific UHPLC system. The gradient was controlled by four pumps. The mobile phase consisted of water containing 0.1% v/v formic acid (phase A) and acetonitrile containing 0.1% v/v formic acid (phase B). The gradient for the preparative separation of [**1**]Cl_2_‐[**3**]Cl_2_ was 30−40% phase B/phase A for 15 min with a flow rate 14 mL min^−1^. The fractions were monitored by four UV detectors (set at 214, 290, 350, and 450 nm) and collected based on the trace of the UV detector (290 nm). After purification, the Δ/Λ stereochemistry of each compound was assigned by CD measurements. The characterization details for the six diastereomers are shown below and in the Supporting Information file.

[Ru(Ph_2_phen)_2_(Ac‐MRGDM‐NH_2_)]Cl_2_ ([1]Cl_2_)


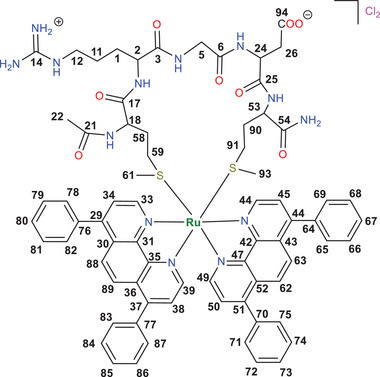


**Δ‐**[**1**]Cl_2_ (6.41 mg, 4.3 µmol, 5%). **HPLC** R_T_: 10.6 min (during purification). ^1^H NMR (600 MHz, δ in CD_3_OD, 293 K): δ 9.95 (d, J = 5.4 Hz, 1H, 39), 9.88 (d, J = 5.4 Hz, 1H, 49), 8.41 – 8.39 (m, J = 9.4 Hz, 3H, 33,38,46), 8.33 (d, J = 5.3 Hz, 1H, 50), 8.25 (t, J = 9.5 Hz, 2H, 34,45), 7.99 (d, J = 5.6 Hz, 1H, 62), 7.96 – 7.93 (m, 1H, 89), 7.92 – 7.88 (m, 4H, 65,69,78,82), 7.75 (td, J = 7.5 Hz, 4H, 66,68,79,81), 7.70 (dt, J = 7.2, 3.8 Hz, 2H, 67,80), 7.57 (td, J = 7.1, 6.0, 2.6 Hz, 12H, 63,71‐75,83‐88), 4.62 (t, 1H), 4.53 (t, 1H), 4.23 (dd, 1H), 4.13 – 4.07 (m, 1H), 4.05 – 3.96 (m, 2H), 3.44 (d, 1H), 3.14 (ddd, 3H), 2.73 – 2.60 (m, 2H), 2.40 (d, 1H), 2.25 – 2.17 (m, 2H), 2.05 – 1.92 (m, 3H), 1.87 (s, 3H), 1.84 – 1.79 (m, 2H), 1.75 (dd, 2H), 1.55 (s, 6H). **UV−vis (H_2_O)**: λ_max_ in nm (*ε* in M^−1^ cm^−1^) = 405 nm (1.21 × 10^4^). **HR‐MS** in CH_3_CN *m/z* experimental (calcd): 707.71692 (707.71769 for [M‐2Cl]^2+^, [C_72_H_75_N_13_O_8_RuS_2_]^2+^), 472.14719 (472.14755 for [M‐2Cl+H]^3+^, [C_72_H_76_N_13_O_8_RuS_2_]^3+^). Elemental analysis calcd (%) for [C_72_H_75_Cl_2_N_13_O_8_RuS_2_ +3 H_2_O +3 NaCOOH]: C, 51.63; H, 4.85; N, 10.44. Found: C, 51.70; H, 4.29; N, 10.01. Notably, the low pure yield was attributed to an incomplete reaction and conservative HPLC purification strategy, which was also applicable to the rest isomers.

**Λ‐**[**1**]Cl_2_ (8.03 mg, 5.4 µmol, 6%). **HPLC** R_T_: 9.96 min (during purification). **^1^H NMR** (850 MHz, δ in CD_3_OD, 293 K): δ 9.99 (dd, *J* = 5.3 Hz, 2H, 39,49), 8.43 – 8.36 (m, 2H, 33,38,46), 8.34 (d, *J* = 5.3 Hz, 1H, 50), 8.25 (dd, *J* = 9.4, 4.1 Hz, 2H, 34,45), 7.96 (t, *J* = 5.5 Hz, 2H, 62,89), 7.91 (t, *J* = 8.2 Hz, 4H, 68,69,78,82), 7.74 (t, *J* = 7.5 Hz, 4H, 68,68,79,81), 7.69 (t, *J* = 7.5 Hz, 2H, 67,80), 7.60 (dd, *J* = 8.6, 5.6 Hz, 2H, 63,88), 7.57 (tt, *J* = 7.7, 3.4 Hz, 10H, 71–75,83‐87), 4.65 (s, 1H, 2), 4.47 (d, 1H, 18), 4.39 (s, 1H, 53), 4.02 (s, 2H, 5), 3.86 (d,  1H, 24), 3.20 (dt, 2H, 12), 2.97 (d, 2H, 1), 2.76 (dd, 2H, 26), 2.29 – 2.01 (m, 5H, 11,59,91), 1.94 (d, 4H, 58,90), 1.79 (s, 3H, 22), 1.77 (s, 3H, 61), 1.64 (s, 3H, 93). **UV−vis (H_2_O)**: λ_max_ in nm (*ε* in M^−1^ cm^−1^) = 405 nm (1.18 × 10^4^). **HR‐MS** in CH_3_CN *m/z* experimental (calcd): 707.71689 (707.71769 for [M‐2Cl]^2+^, [C_72_H_75_N_13_O_8_RuS_2_]^2+^), 472.14706 (472.14755 for [M‐2Cl+H]^3+^, [C_72_H_76_N_13_O_8_RuS_2_]^3+^). Elemental analysis calcd (%) for [C_72_H_75_Cl_2_N_13_O_8_RuS_2_ +8 H_2_O +4 NaCOOH+3 C_2_H_5_OH]: C, 48.26; H, 5.58; N, 8.92. Found: C, 48.46; H, 5.70; N, 8.77.

[Ru(Ph_2_phen)_2_(Ac‐mrGdm‐NH_2_)]Cl_2_ ([2]Cl_2_)

**Δ‐**[**2**]Cl_2_ (10.5 mg, 7.1 µmol, 7%). **HPLC** R_T_: 10.0 min (during purification). ^1^H NMR (850 MHz, δ in CD_3_OD, 293 K): δ 9.99 (dd, *J* = 5.2 Hz, 2H, 39,49), 8.39 (m, 3H, 33,38,46), 8.34 (d, *J* = 5.1 Hz, 1H, 50), 8.25 (dd, *J* = 9.4, 5.0 Hz, 2H, 34,45), 7.96 (dd, *J* = 5.6, 2.6 Hz, 2H, 62,89), 7.90 (dd, *J* = 9.8, 7.4 Hz, 4H, 68,69,78,82), 7.75 (t, *J* = 7.5 Hz, 4H, 66,68,79,81), 7.69 (t, *J* = 7.5 Hz, 2H, 67,80), 7.61 (dd, *J* = 5.6 Hz, 2H, 63,88), 7.57 (qt, *J* = 5.8, 3.1 Hz, 10H, 71–75,83‐87), 4.66 (s, 1H, 2), 4.48 (s, 1H, 18), 4.39 (s, 1H, 53), 4.04 (d, 1H, 5), 3.99 (s, 1H, 5), 3.86 (d, 1H, 24), 3.22 – 3.17 (m, 2H, 12), 3.01 (dd, 2H, 1), 2.75 (dd, 2H, 26), 2.20 (dt, 2H, 11), 2.12 – 2.07 (m, 4H, 59,91), 2.06 – 2.01 (m, 2H, 58,90), 1.91 – 1.87 (m, 3H, 22), 1.79 (s, 3H, 61), 1.64 (s, 3H, 93). ^13^C NMR (214 MHz, δ in CD_3_OD, 293 K) δ 174.22, 172.73, 172.56, 171.97, 171.66, 169.62 (C = O, 3,6,14, 21,25,54,94), 157.17 (Cq), 153.83, 151.77, 151.42, 151.32 (CH_2_), 150.68, 150.63, 150.55, 150.50, 148.80, 148.77, 147.81, 147.71, 135.68, 135.68, 135.33, 135.27 (Cq, arom), 129.99, 129.94, 129.74, 129.63, 129.61, 129.57, 129.02, 129.01, 128.94, 128.84, 128.82, 127.10, 126.91, 126.34, 126.32, 126.01 (CH, arom), 129.69, 129.59, 129.52, 129.47, 128.97, 128.91 (Cq, arom), 52.79, 51.51, 50.46, 48.08, 47,98 (CH, 2,18,24,53), 41.57, 40.47, 35.30, 32.65, 32.16, 29.90, 24.79 (CH_2_, 1,11,12,26,58,59,90,91), 20.94 (CH_3_, 22), 15.97 (CH_3_, 61), 14.76 (CH_3_, 93). **UV−vis (H_2_O)**: λ_max_ in nm (*ε* in M^−1^ cm^−1^) = 405 nm (1.16 × 10^4^). **HR‐MS** in CH_3_CN *m/z* experimental (calcd): 707.71631 (707.71769 for [M‐2Cl]^2+^, [C_72_H_75_N_13_O_8_RuS_2_]^2+^), 472.14682 (472.14755 for [M‐2Cl+H]^3+^, [C_72_H_76_N_13_O_8_RuS_2_]^3+^). Elemental analysis calcd (%) for [C_72_H_75_Cl_2_N_13_O_8_RuS_2_ +5 H_2_O +1 NaCOOH+3 C_2_H_5_OH]: C, 53.22; H, 5.88; N, 10.21. Found: C, 53.20; H, 5.83; N, 9.21.

**Λ‐**[**2**]Cl_2_ (12.9 mg, 8.7 µmol, 9%). HPLC R_T_: 10.6 min (during purification). ^1^H NMR (850 MHz, δ in CD_3_OD, 293 K): δ 9.97 (d, *J* = 5.3 Hz, 1H, 39), 9.86 (d, *J* = 5.3 Hz, 1H, 49), 8.39 (dd, *J* = 9.4 Hz, 3H, 33,38,46), 8.34 (dd, *J* = 5.2 Hz, 1H, 50), 8.25 (t, *J* = 9.9 Hz, 1H, 34,45), 8.01 (d, *J* = 5.7 Hz, 2H, 62,89), 7.91 (dd, *J* = 7.0 Hz, 4H, 65,69,78,82), 7.74 (q, *J* = 7.4 Hz, 4H, 66,68,79,81), 7.69 (dt, *J* = 7.5 Hz, 2H, 67,80), 7.58 (m, 12H, 63,71‐75,83‐88), 4.51 (d, 1H, 2), 4.67 (s, 1H, 18), 4.19 (dd, 1H, 53), 4.02 (t, 2H, 5), 3.93 (d, 1H, 24), 3.19 (m, 2H, 12), 3.10 – 3.05 (m, 2H, 1), 2.79 (s, 2H, 26), 2.29 (d, 2H, 11), 2.14 – 2.10 (m, 4H, 59,91), 2.10 – 2.05 (m, 4H, 58,90), 1.92 (s, 3H, 22), 1.55 (s, 3H, 61), 1.52 (s, 3H, 93). ^13^C NMR (214 MHz, δ in CD_3_OD, 293 K) δ 173.96, 172.94, 172.81, 172.34, 170.96 (C = O 3,6,14, 21,25,54,94), 157.37, 157.32 (Cq), 154.06, 153.71, 153.64, 151.40, 151.32 (CH), 150.73, 150.55, 150.51, 150.38, 148.78, 148.75, 147.78, 147.77, 135.68, 135.67, 135.30, 135.27 (Cq), 130.00, 129.98, 129.87, 129.81, 129.72, 129.66, 129.61, 129.01, 128.86, 126.32, 126.06, 126.00 (CH, arom), 129.51, 129.46, 128.90, 128.88 (Cq, arom), 55.08, 53.18, 52.75, 47.91 (CH, 2,18,24,53), 46.44, 43.83, 40.56, 31.20, 25.57 (CH_2_, 1,11,12,26,58,59,90,91), 21.16 (CH_3_, 22), 14.60 (CH_3_, 61), 14.35 (CH_3_, 93). **UV−vis (H_2_O)**: λ_max_ in nm (*ε* in M^−1^ cm^−1^) = 405 nm (1.27 × 10^4^). **HR‐MS** in CH_3_CN *m/z* experimental (calcd): 707.71662 (707.71769 for [M‐2Cl]^2+^, [C_72_H_75_N_13_O_8_RuS_2_]^2+^), 472.14711 (472.14755 for [M‐2Cl+H]^3+^, [C_72_H_76_N_13_O_8_RuS_2_]^3+^). Elemental analysis calcd (%) for [C_72_H_75_Cl_2_N_13_O_8_RuS_2_ +2 H_2_O +1 NaCOOH]: C, 55.12; H, 5.07; N, 11.45. Found: C, 55.53; H, 5.07; N, 11.23.

[Ru(Ph_2_phen)_2_(Ac‐MrGdM‐NH_2_)]Cl_2_ ([3]Cl_2_)

**Δ‐**[**3**]Cl_2_. (1.58 mg, 1.1 µmol, 2%). **HPLC** R_T_: 10.2 min (during purification). **^1^H NMR** (850 MHz, δ in CD_3_OD, 293 K): δ 9.94 (d, *J* = 5.1 Hz, 1H, 39), 9.83 (d, *J* = 5.4 Hz, 1H, 49), 8.51 (d, *J* = 5.3 Hz, 1H, 50), 8.36 (dt, *J* = 8.0 Hz, 3H, 33,38,46), 8.24 (t, *J* = 9.7 Hz, 2H, 34,45), 7.94 – 7.87 (m, 6H, 62,65,68,79,81,89), 7.74 (dd, *J* = 7.6 Hz, 4H, 66,68,79,81), 7.72 – 7.66 (m, 2H, 67,80), 7.56 (m, 10H, 71–75, 83–87), 4.23 (s, 1H, 18), 4.20 (s, 1H, 53), 4.08 (d, 2H, 5), 3.93 (d, 2H, 2,24), 2.99 (d, 1H, 12), 2.96 (s, 1H, 12), 2.71 – 2.67 (m, 2H, 1), 2.63 (s, 2H, 26), 2.47 (s, 2H, 11), 2.11 (s, 4H, 58,59,90,91), 1.71 (s, 3H, 22), 1.53 (s, 6H, 61,93). ^13^C NMR (214 MHz, δ in CD_3_OD, 293 K) δ 172.31, 172.14, 172.08, 171.78, 168.58 (C = O, 3,6,14, 21,25,54,94), 156.34 (Cq) 153.08, 150.38 (CH), 149.87, 149.82, 149.57, 149.52, 147.92, 146.89, 146.78, 134.75, 134.72, 134.42, 134.37 (Cq, arom), 129.18, 129.01, 128.88, 128.74, 128.70, 128.65, 128.13, 128.02, 127.95, 126.58, 126.10, 125.44, 125.41, 125.15, 125.01 (CH, arom), 53.69, 52.91, 52.68, 49.05 (CH, 2,18,24,53), 42.76, 39.63, 32.13, 30.14, 28.17, 28.07, 25.14 (CH_2_, 1,11,12,26,58,59,90,91), 20.56 (CH_3_, 22), 14.90 (CH_3_, 61), 14.27 (CH_3_, 93). **UV−vis (H_2_O)**: λ_max_ in nm (*ε* in M^−1^ cm^−1^) = 405 nm (1.05 × 10^4^). **HR‐MS** in CH_3_CN *m/z* experimental (calcd): 707.71711 (707.71769 for [M‐2Cl]^2+^, [C_72_H_75_N_13_O_8_RuS_2_]^2+^), 472.14722 (472.14755 for [M‐2Cl+H]^3+^, [C_72_H_76_N_13_O_8_RuS_2_]^3+^). Elemental analysis calcd (%) for [C_72_H_75_Cl_2_N_13_O_8_RuS_2_ +6 H_2_O +5 HCOOH]: C, 50.68; H, 5.36; N, 9.98. Found: C, 50.85; H, 5.81; N, 9.53.

**Λ‐**[**3**]Cl_2_ (3.18 mg, 2.1 µmol, 4%). **HPLC** R_T_: 11.4 min (during purification). ^1^H NMR (850 MHz, δ in CD_3_OD, 293 K):^1^H NMR (850 MHz, δ in CD_3_OD, 293 K) δ 9.94 (d, *J* = 5.3 Hz, 1H, 39), 9.85 (d, *J* = 5.3 Hz, 1H, 49), 8.41 (dd, *J* = 9.4, 5.1 Hz, 2H, 33,46), 8.33 (d, *J* = 5.3 Hz, 1H, 50), 8.27 (dd, *J* = 9.4, 2.9 Hz, 2H, 34,35), 7.99 (d, *J* = 5.6 Hz, 1H, 62), 7.95 (d, *J* = 5.6 Hz, 1H, 89), 7.92 (dd, *J* = 7.4 Hz, 4H, 65,69,78,82), 7.76 (td, *J* = 7.7, 1.9 Hz, 4H, 66,68,79,81), 7.74 – 7.72 (m, 2H, 67–80), 7.71 – 7.65 (m, 2H, 63,88), 7.62 – 7.54 (m, 10H, 71–75,83‐87), 4.51 (t, *J* = 4.2 Hz, 1H, 2), 4.24 (dd, 1H, 18), 4.13 (s, 1H, 53), 4.05‐4.01 (dd, 2H, 5), 3.95 (t, 1H, 24), 3.22 – 3.18 (m, 2H, 12), 3.02 (dd, 1H, 1), 2.59 (dd, 1H, 26), 2.42 (s, 1H), 2.23 (dt, 2H, 11), 2.12 (s, 4H, 59,91), 2.00 (s, 4H, 58,90), 1.75 (s, 3H, 22), 1.58 (ds, 6H, 61,93). ^13^C NMR (214 MHz, MeOD) δ 177.09, 174.40, 173.15, 172.95, 172.13, 170.25 (C = O, 3,6,14, 21,25,54,94), 157.47 (Cq), 151.04, 151.23 (CH), 150.77, 150.69, 150.55, 150.48, 148.80, 148.75, 147.78, 147.74 (Cq, arom), 135.70, 135.66, 135.28, 135.28 (Cq, arom), 129.81, 129.67, 129.59, 129.50, 129.48, 129.14, 128.90, 127.04, 126.30, 125.97 (CH, arom), 54.74, 53.81, 52.95, 50.42 (CH, 2,18,24,53) 40.87, 37.44, 33.28, 32.15, 29.83, 29.25, 25.22 (CH_2_, 1,11,12,26,58,59,90,91), 20.79 (CH_3_, 22), 15.17 (CH_3_, 61,93). **UV−vis (H_2_O)**: λ_max_ in nm (*ε* in M^−1^ cm^−1^) = 405 nm (1.12 × 10^4^). **HR‐MS** in CH_3_CN *m/z* experimental (calcd): 707.71668 (707.71769 for [M‐2Cl]^2+^, [C_72_H_75_N_13_O_8_RuS_2_]^2+^), 472.14702 (472.14755 for [M‐2Cl+H]^3+^, [C_72_H_76_N_13_O_8_RuS_2_]^3+^). Elemental analysis calcd (%) for [C_72_H_75_Cl_2_N_13_O_8_RuS_2_ +1 H_2_O +1 HCOOH]: C, 56.55; H, 5.14; N, 11.74. Found: C, 56.93; H, 5.11; N, 11.62.

### Photosubstitution Quantum Yields Calculation

The obtained time‐dependent absorbance matrix A = f(t, λ) was globally fitted using the Glotaran software (graphical interface to R‐package TIMP),^[^ ^74^, ^75^ ^]^ which afforded the species‐associated spectra of [**1**]^2+^‐[**3**]^2+^ (Ru‐S, S called R for reactant), Ru‐S, OH_2_ or Ru‐S, CH_3_CN (called I for intermediate), and Ru‐(OH_2_)_2_ or Ru‐(NCCH_3_)_2_ (called P for product) species, and their relative concentration profiles. The concentration of reactants at t = 0 in H_2_O/MeCN was derived from molar absorption coefficient (*ε*) measurements for the different reagents R. The *ε* values of the intermediates I and products P were calculated from the data fitted by Glotaran. From the *ε* values and relative concentration evolution of the three species, the number of mol *n* **_R_**, *n* **_I_**, and *n* **_P_** was derived in Microsoft Excel 365. The number of photons absorbed by the reactant (*Q* **_R_**) and by the intermediate I (*Q* **_I_**) were calculated as well. Finally, the slope of a plot of *n* **_R_** versus *Q* **_R_** and of *n* **_I_** versus *Q* **_I_** gave the quantum yield for the first and second photosubstitution step, respectively.^[^ ^76^ ^]^

### Cellular Uptake

The intracellular ruthenium uptake by 2D A549 and PC‐3 cell monolayers was assessed by ICP‐MS. Normoxic and hypoxic cells from both cell lines were seeded in 100 µL Opti‐MEM at a density of 5 × 10^3^ (A549) or 6 × 10^3^ (PC‐3) cells in black 96‐well plates (PerkinElmer 6005182). After 24 h, 100 µL of 10 µm drug solution in OptiMEM was added in sextuplicate to A549 cells as well as to PC‐3 cells. 12 wells per plate were filled with 100 µL Opti‐MEM for the control. After another 24 h incubation in the dark, the drug‐containing medium was removed, cells were washed once (150 µL for each well) with phosphate‐buffered saline (PBS), and stained with 50 µL Nuclear Blue (2 drops per mL OptiMEM, Fischer Scientific R37605) for 30 min. Subsequently, the excess dye was removed, fresh medium was added, and each well was imaged using a Nikon TiE2000 confocal laser microscope. Nuclei numbers were calculated by Image‐Pro Analyzer 7.0, resulting in the cell number per well.^[^ ^74^, ^75^ ^]^ After imaging, all medium was removed, and the cells were lysed by adding 100 µL 65% HNO_3_ (1.00441.1000, Sigma) per well. The lysate was diluted by adding 900 µL MilliQ water (10 × dilution) in a deep well plate (Eppendorf, E951033502), and the ruthenium content of every 1 mL well was determined by ICP‐MS (PerkinElmer NexION 2000) in ppb (µg L^−1^). In Microsoft Excel 365, the ruthenium uptake values were calculated in µg Ru per million cells by dividing the determined Ru content per well in µg by the number of counted cells per well. Mean values (*n* = 6) and standard deviation were reported in Figure 3a. Significance between data sets was calculated in Microsoft Excel 365 by a two‐sample unequal variance Student's *t*‐test using a two‐tailed distribution.

### 2D Cytotoxicity Assay

A549 cells (5000) or PC‐3 cells (6000) were seeded in 96‐well plates (Sarstedt, 83.3924), each well contained 100 µL Opti‐MEM (Gibco complete medium 11058‐021, supplemented with 2.5% v/v fetal calf serum (FCS), 0.2% v/v penicillin/streptomycin (P/S), and 1% v/v glutamine) either in normoxic (21% O_2_) or hypoxic (1.0% O_2_) incubator. 24 h later, different concentrations (0.5, 2.5, 5, 10, 25, 50 µm) of Δ‐[**1**]Cl_2_, Λ‐[**1**]Cl_2_, Δ‐[**2**]Cl_2_, Λ‐[**2**]Cl_2_, Δ‐[**3**]Cl_2_ or Λ‐[**3**]Cl_2_ dissolved in Opti‐MEM (100 µL) were added to the wells in triplicate. For each complex, 1 dark and 1 light plate were prepared. Each plate was further incubated in the dark for 24 h (DLI). After that, the light plate was irradiated with green light (520 nm) for 20 min at 37 °C for normoxia (power density = 10.9 mW cm^−2^, dose = 13.1 J cm^−2^) or 30 min for hypoxia (power density = 7.22 mW cm^−2^, dose = 13.0 J cm^−2^), while the other plate was kept in the dark. The cells were further incubated for another 2 days in normoxic or hypoxic dark conditions, respectively. Finally, 100 µL of cold trichloroacetic acid (10% w/v) was added to each well to fix the cells, and all plates were then transferred to a 4 °C refrigerator for 48 h before performing an SRB cell quantification endpoint assay (see below).^[^ ^66^ ^]^ All experiments were conducted in biologically independent triplicate. Half‐maximal effective concentrations (EC_50_) for 3D tumor spheroid growth inhibition were calculated by GraphPad Prism 5 using the dose‐response two‐parameter Hill slope equation (Equation 1).^77^ Errors were calculated utilizing 95% confidence intervals (±CI in µm).

(1) $$100/\left(1+10^{\left(\left(\mathrm{lo}g_{10}EC_{50}-X\right)\times \mathrm{Hill}\;\mathrm{Slope}\right)}\right)$$

### 2D Cytotoxicity Assay—In Vitro Hypoxic Cell Models

All cells were incubated and passaged in a dark hypoxic incubator (1% O_2_) for at least two weeks before all hypoxia studies. Adding chemicals to the cells had to be performed in air, but the cell‐growing medium was kept in the hypoxia incubator for at least 2 days before addition to hypoxic cells, and all hypoxic light irradiations^[^ ^75^ ^]^ were performed inside the hypoxic incubator set at 1.0% O_2_.

### 2D Cytotoxicity Assay—SRB Staining Protocol

Trichloroacetic acid was first removed, and plates were gently washed with demi water for 3–5 times. After each well was dried in the air, 100 µL 0.6% SRB solution (0.6% w/v in 1% v/v acetic acid/H_2_O solution) was added into the wells and allowed to stain the cells for 30 min. Then each plate was washed 3–5 times using acetic acid solution (1% v/v), which removed dead cells, but also all unbound ruthenium complexes and the rest of the medium; the plates were then allowed to dry overnight. After that, 200 µL 10 mm Tris base buffer was added to the wells, and the plates were allowed to sit on an orbital shaker for 0.5–16 h. After mixing well, the absorbance of each well was determined by M1000 Tecan Reader, reading at 510 nm.

### Viability Assay of 3D Tumor Spheroids

A549 cells (700) were added to a 96‐well round‐bottomed Corning spheroid plate (Catalogue CLS4520) microplate and incubated under normoxia (21% O_2_) for 4 days to generate 3D tumor spheroids. Spheroids were grown in 100 µL Opti‐MEM (Gibco complete medium 11058‐021, supplemented with 2.5% v/v fetal calf serum (FCS), 0.2% v/v penicillin/streptomycin (P/S), and 1% v/v glutamine). 1 dark and 1 light plate were included in each group. After that, 100 µL of different concentrations of [**1]**Cl_2_ **‐[2]**Cl_2_ dissolved in Opti‐MEM were added to each well in triplicate to reach final concentrations in the wells of 0, 1, 2.5, 5, 10, 25, 50, and 100 µm. The spheroids were incubated further under normoxia for 24 h. Then, without a washing step, the light plate was irradiated with the green light for 30 min (dose = 13.1 J cm^−2^, wavelength = 520 nm, intensity = 7.22 mW cm^−2^), and the other plate was left in the dark. The cells were further incubated under normoxia in the dark for 2 days. A CellTiter Glo 3D solution (100 µL per well, Cat. G9683, Promega, no further dilution) was added to each well (to 300 µL final volume) to stain the 3D tumor spheroids. After 30 min of shaking on an IKA Vibrax shaker at 500 rpm at room temperature, the luminescence (560 nm) in each well was measured with a Tecan microplate reader. Similar to 2D cell cultures, half‐maximal effective concentrations (EC_50_) for 3D tumor spheroid growth inhibition were calculated by GraphPad Prism 5 using the dose‐response two‐parameter Hill slope equation (Equation 1). All experiments were conducted in biologically independent triplicate.

### Measurement of Intracellular ROS

The generation of ROS (reactive oxygen species) in A549 cells was measured using the ROS deep red fluorescence indicator (Abcam, ab186029). A549 (1 × 10^5^, 1 mL) were seeded into 12‐well plates (Sarstedt, 83.3921) and incubated for 24 h in the dark under normoxia. The cells were then treated with [**1]**Cl_2_ **‐[3]**Cl_2_, cisplatin, Rose Bengal or **[Ru(Ph_2_phen)_2_Cl_2_]** (15 µm). There were two groups for each drug (dark and light). After 24 h of incubation under normoxia, the plate was washed with cold PBS once, and cells were trypsinized, harvested, and then resuspended in 150 µL PBS. The cell suspension from the centrifuge tubes was transferred to 96‐round bottom well plates (Thermo Scientific, 268200), and the plates were kept in the dark or irradiated with 520 nm light (dose = 13.1 J cm^−2^). After which, the Cellular ROS Deep Red dye was added with 1000× dilution, and the cells were further stained for 1 h. The levels of intracellular ROS were then determined using the CytoFLEX flow cytometer using the APC‐A (638 nm excitation, 660/10 nm emission) channel. All flow cytometry data were processed using FlowLogic 8.5 software.

### Photoactivated Protein Binding

The mixture of Λ‐[**1**]Cl_2_ (10 µm) and BSA (50 µm) was prepared in PBS buffer, and divided into 2 groups: named dark and light. The light group was irradiated with green light (515 nm, 10 mW cm^−2^, 30 min), and emission spectra (λ_em_ = 480 nm, collection window: 550–1000 nm) were collected before and after light irradiation. After that, the solution was kept in the dark, and the spectra were collected at 1, 4, 8, 12, and 24 h after irradiation. In the meantime, the spectra of the dark group solution were recorded at the same time point.

### Subcutaneous Solid Tumor Model Construction

All animal studies were approved by the Institutional Animal Care and Use Committee of the US National Institutes of Health and were performed according to the relevant guidelines (8th edition, 2011. License number: IACUC‐FPH‐SL‐20231020[0183). Female BALB/c nude mice (6 weeks old) were purchased from Shanghai SLAC Laboratory Animal Co. LTD (Shanghai, China). Tumor‐bearing mice were obtained by subcutaneously injecting A549 tumor cells (2 × 10^6^ cells per mouse, dispersed in 100 µL of FBS (ZETA, Z7185FBS‐500) into the right hind limb and waiting for 10 days until the tumor volume reached 50–100 mm^3^.

### Biodistribution Evaluation

The BALB/c nude mice bearing A549 tumors were randomly assigned to 3 groups (*N*  = 15) and received an intravenous injection of Λ‐[**1**]Cl_2_ Λ‐[**2**]Cl_2_ or Λ‐[**3**]Cl_2_  (7.7 mg kg^−1^) at the same molar dose. Then, the mice were sacrificed (cervical dislocation after the process of isoflurane anesthesia) at 2, 6, 12, 18, and 24 h post‐injection (each time point contained 3 mice). The main organs (heart, liver, spleen, kidney, lung) and tumor tissue were dissected. Then, ≈1 g of organs and tumors was lysed in a 7 mL mixture solution containing 5 mL of 65% HNO_3_ and 2 mL of 30% (w/w) H_2_O_2_ at 100 °C. After 12 h, all solutions had evaporated, and 3 mL of aqueous solution containing 2% HNO_3_ was added. The Ru content in all samples was measured by ICP‐OES (JY‐Horiba ICP‐OES Ultima 2).

### In Vivo Tumor Inhibition Studies

The mice bearing A549 solid tumors were first randomly divided into 8 groups (*N*  = 5): PBS, Laser, Λ‐[**1**]Cl_2_, Λ‐[**2**]Cl_2_, Λ‐[**3**]Cl_2_, Λ‐[**1**]Cl_2_ + Laser, Λ‐[**2**]Cl_2_ + Laser and Λ‐[**3**]Cl_2_ + Laser. The injectable solution of Λ‐[**1**]Cl_2_‐[**3**]Cl_2_ was obtained by diluting the stock solution of the compound in DMSO (10 mg mL^−1^) ten times by RPMI‐1640 medium containing 10% FBS, 100 units mL^−1^ penicillin, and 100 µg mL^−1^ streptomycin. The injection doses of the complexes were set as 7.7 mg kg^−1^ (injection volume: ≈170 µL in 5 s determined by the weight of each mouse, the concentration of working solution: 1 mg mL^−1^, the average weight of mice was ≈22 g). In groups 2, 6, 7, and 8, laser irradiation (520 nm, 100 mW cm^−2^, 5 min) was carried out twice at 12 h post‐injection, with a 5 min interval. Accordingly, the total laser dose for each illumination was 100 mW cm^−2^, 10 min, 60 J cm^−2^. Treatments in all groups were carried out only once on day 0. Digital photos of tumor‐bearing mice, the tumor size (length and width, measured with a caliper), and mouse body weight in all groups were recorded every third day. The tumor volume was calculated according to the standard formula: 0.5 × length × width.^2^ Both the average tumor volume and body weight were followed for 14 days. On day 7, one mouse from each group was sacrificed, and then the tumor tissue was dissected and fixed with 10% paraformaldehyde. The tumor cell damage and apoptosis, and necrosis conditions were evaluated by H&E or TUNEL‐stained protocols. On day 14 (the end of the tumor treatment period), all nude mice were humanely sacrificed, and the main organs (heart, liver, spleen, kidney, and lung) and tumors were resected. Digital photographs of tumors in each group were immediately obtained. All normal tissues were fixed with 10% paraformaldehyde and further analyzed in accordance with the H&E staining protocol to estimate their off‐target side effect after various treatments.

### In Vivo Optical Fluorescence Imaging

A bilateral primary (left side) and distant (right side) lung tumors model was obtained by subcutaneously injecting A549 tumor cells (2 × 10^6^ cells per side, dispersed in 100 µL of FBS) into the left and right hind limbs and waiting for 10 days until the tumor volumes reached 50–100 mm^3^. Then, above BALB /c nude mice bearing bilateral A549 tumors were randomly assigned to 3 groups (*N*  = 3) and received an intravenous injection of Λ‐[**1**]Cl_2_ Λ‐[**2**]Cl_2_, or Λ‐[**3**]Cl_2_ (7.7 mg kg^−1^) at the same molar dose. Then all distant tumors on the right side were irradiated by a laser (520 nm, 100 mW cm^−2^, 5 min) twice at 12 h post‐injection with a 5 min interval. A IVIS Lumina II In Vivo Imaging System (Ex = 488 nm, 600 nm long‐pass filter) was applied in animal fluorescence imaging at 12, 24, 36, and 48 h post‐illumination.

## Conflict of Interest

The authors declare no conflict of interest.

## Supporting information



Supporting Information

## Data Availability

The data that support the findings of this study are available from the corresponding author upon reasonable request.
