# Supplementary material for: Photoactivated Proximity Protein Labeling Reveals Enhanced Tumor Retention of a D‐Peptide‐Ruthenium Prodrug Conjugate
Source: Adv Healthc Mater. 2025 Oct 20;15(5):e02174. doi: 10.1002/adhm.202502174 (PMC12864594; doi:10.1002/adhm.202502174)
Supplement: Supplementary file 1 — Supporting Information [file ADHM-15-0-s001.docx]

**Photoactivated proximity protein labeling reveals enhanced tumor retention of a D-peptide-ruthenium prodrug conjugate**

*Liyan Zhang ^a^, Peiyuan Wang ^b, c^, Hildert Bronkhorst ^a^,* *Yurii Husiev ^a^,* *Ludovic Bretin ^a^, Maarten N. van Ginkel ^a^, Wen Sun ^b*^ & Sylvestre Bonnet ^a*^*

^a^ Leiden Institute of Chemistry, Universiteit Leiden, Einsteinweg 55, 2333 CC, Leiden, Netherlands

^b^ State Key Laboratory of Fine Chemicals, Dalian University of Technology, 2 Linggong Road, Dalian 116024, P. R. China.

^c^ Key Laboratory of Design and Assembly of Functional Nanostructures, Fujian Institute of Research on the Structure of Matter, Chinese Academy of Sciences, Fuzhou 350002, P. R. China.

Corresponding author email addresses: [bonnet@chem.leidenuniv.nl](mailto:bonnet@chem.leidenuniv.nl), [sunwen@dlut.edu.cn](mailto:sunwen@dlut.edu.cn)

**General information**

All reagents were purchased from commercial suppliers. The reactants and solvents were used without further purification. Electrospray ionization mass spectra were recorded by using a MSQ Plus Spectrometer in the positive ionization mode. ^1^H NMR was obtained on a Bruker DMX-400, 600 or 850 spectrometers. HPLC was accomplished by Thermo Scientific Dionex Ultimate 300 system equipped with a 250 x 21.2 mm Jupiter® 4 µm Proteo 90 Å C_12_ column. UV-vis spectra were recorded on a Cary 60 spectrometer from Varian. The emission spectra were measured via an F900 Spectrometer from Edinburgh Instruments Ltd. Absorbance measurements for analysis of 96-well plates were done by a M1000 Tecan Plate Reader. Flow cytometry was conducted by CytoFLEX flow cytometer. Human cancer cell lines A549 (adenocarcinoma alveolar basal epithelial cells) and PC-3 (prostate cancer cells) were distributed by the European Collection of Cell Cultures (ECACC) and purchased from Sigma Aldrich. Dulbecco’s Modified Eagle Medium (DMEM, D6546), Glutamine-S (GM; 200 mm), penicillin/streptomycin (P/S), tris(hydroxylmethyl)aminomethane (Tris base), trichloroacetic acid (TCA), glacial acetic acid, and sulforhodamine B (SRB) were purchased from Sigma-Aldrich. Opti-MEM Reduced Serum Media without phenol red was obtained from Gibco (11058-021). Rose Bengal and Ru(bpy)_3_Cl_2_ were ordered from Sigma-Aldrich. Cellular ROS Assay Kit (Deep Red, tBHP (tert-Butyl hydroperoxide) included) was purchased from Abcam (ab186029). Anti-integrin α_V_β_3_ antibody for integrin expression study was purchased from Merck (MAB1976), Anti-Integrin α_V_β_5_ antibody [P1F6] was from Abcam (ab177004). The Alexa Fluor™ 488 conjugated goat anti-mouse IgG (H+L) cross-adsorbed secondary antibody, was ordered from Invitrogen by Thermo Fisher Scientific (A-11001).


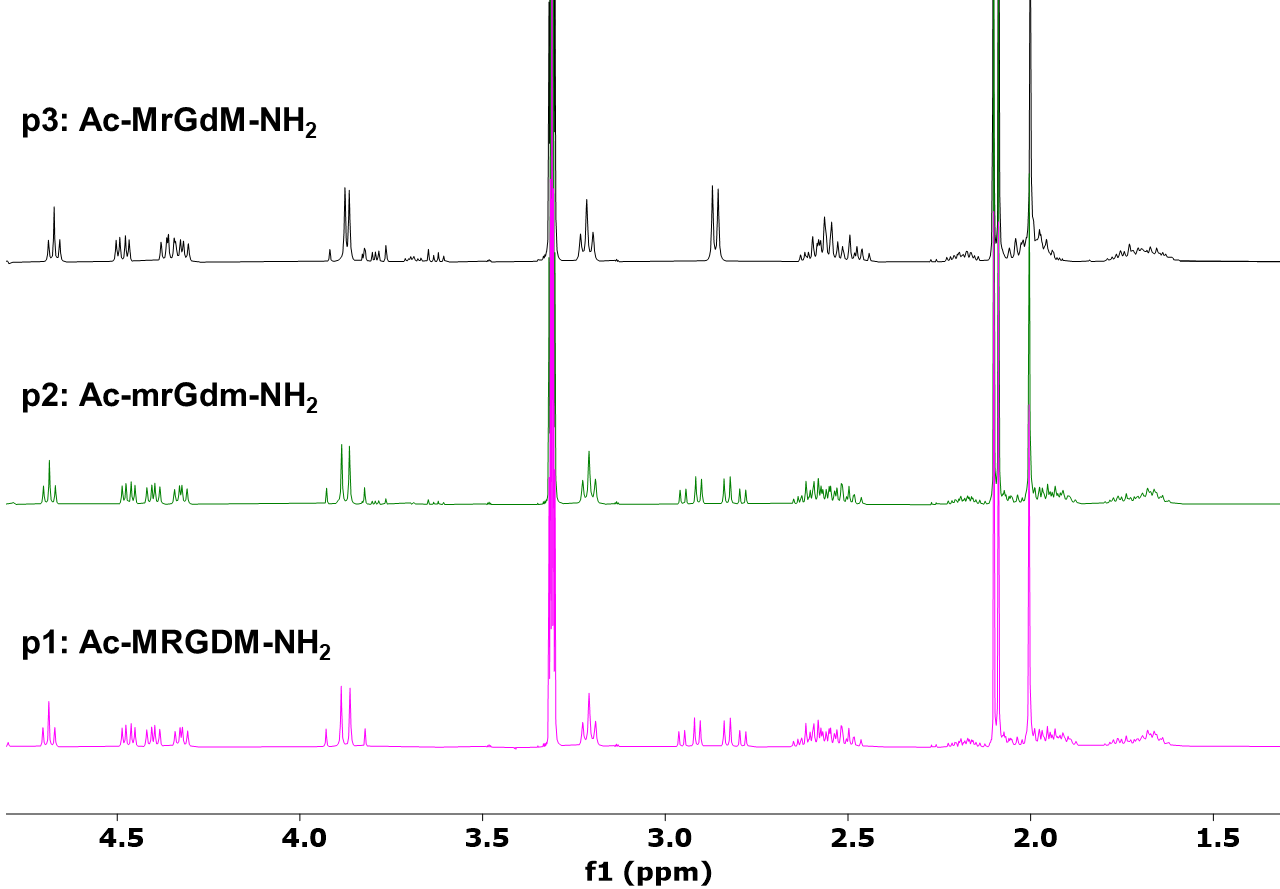


**Figure S1.** ^1^H NMR spectra (400 MHz, CD_3_OD, 293 K) of Ac-MRGDM-NH_2_ (p1), Ac-mrGdm-NH_2_ (p2), Ac-MrGdM-NH_2_ (p3).


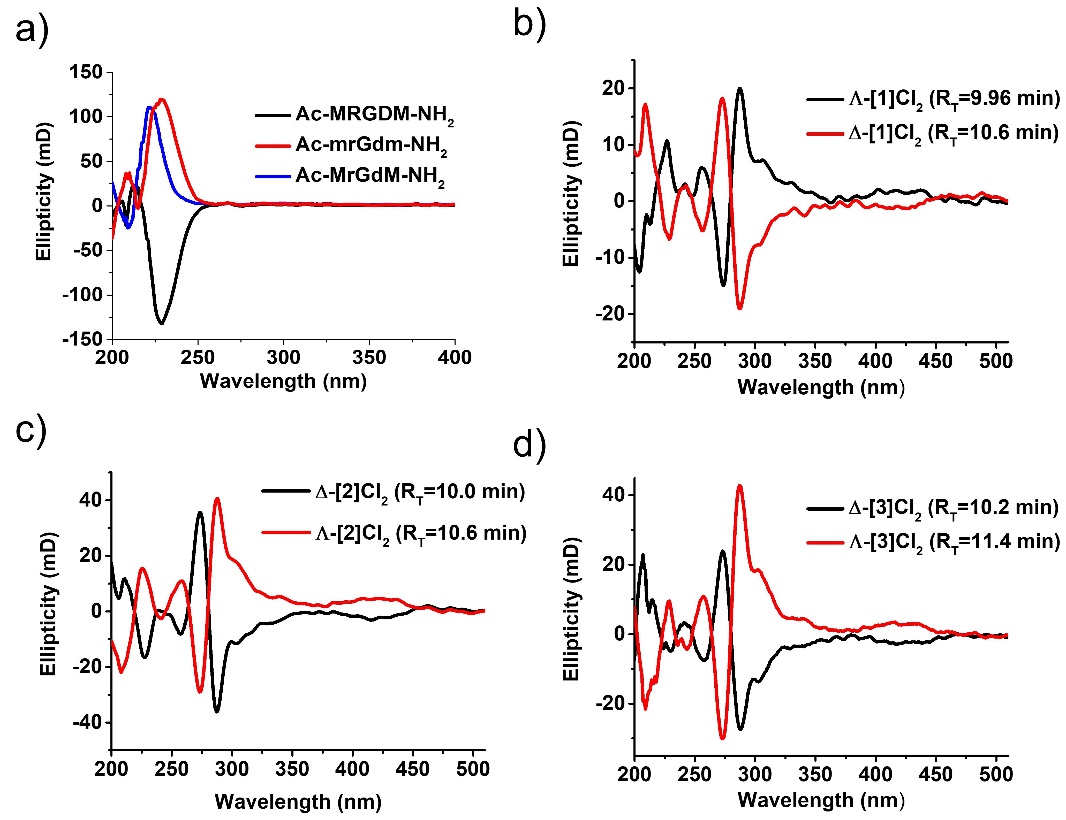


**Figure S2.** (a) CD spectra of the three free peptides Ac-MRGDM-NH_2_ (p1), Ac-mrGdm-NH_2_ (p2) and Ac-MrGdM-NH_2_ (p3). (b)-(d) CD spectra of the Ru-peptide conjugates [**1**]Cl_2_ (b), [**2**]Cl_2_ (c), and [**3**]Cl_2_ (d) after HPLC purification. No CD signal was detected at wavelengths longer than 500 nm.


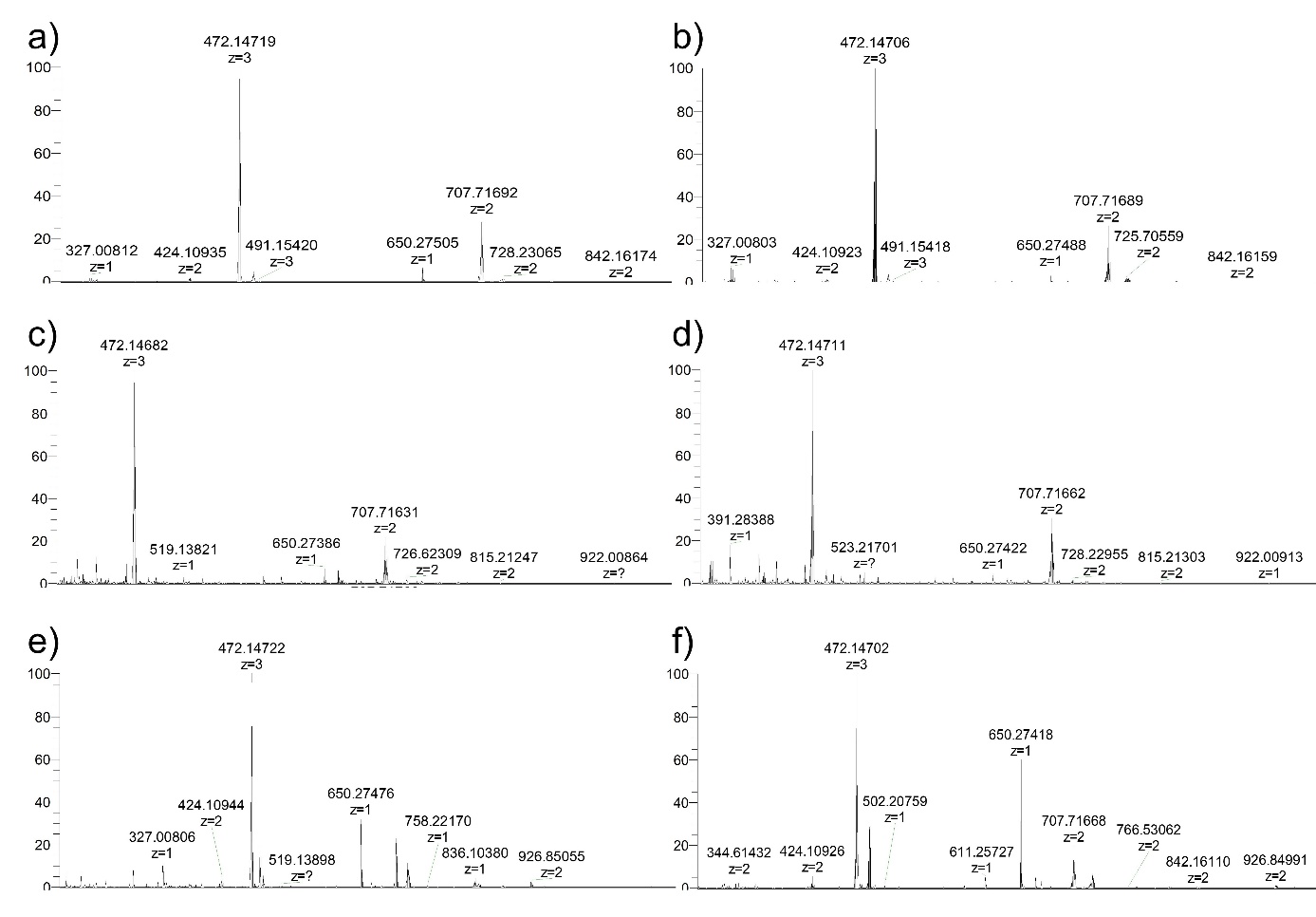


**Figure S3.** High resolution mass spectra of Δ-[**1**]^2+^ (a), Λ-[**1**]^2+^(b), Δ-[**2**]^2+^ (c), Λ-[**2**]^2+^(d), Δ-[**3**]^2+^(e) and Λ-[**3**]^2+^(f). Calculated compound peaks are at 707.7176 m/z ([M – 2Cl]^2+^) and 472.1475 m/z ([M – 2Cl + H]^3+^).


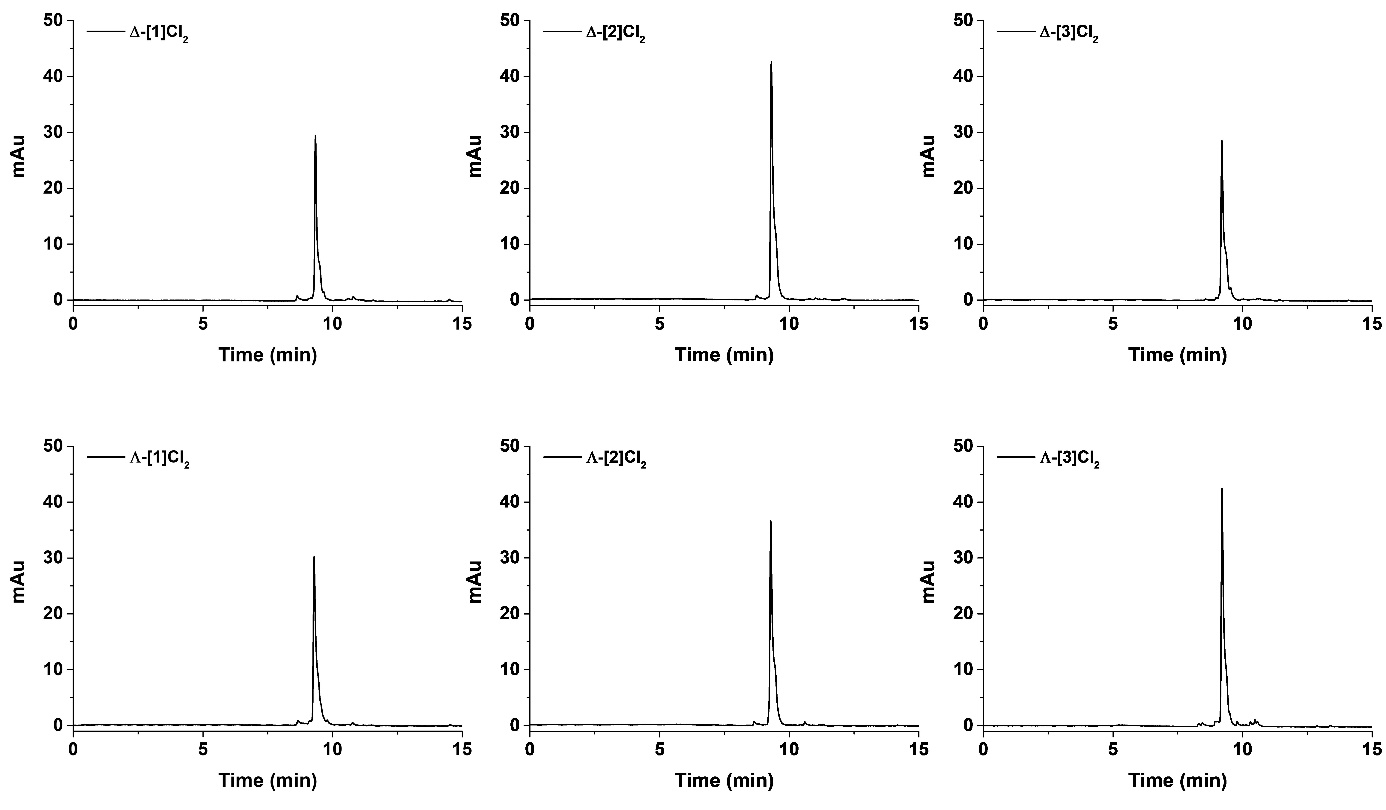


**Figure S4.** HPLC trace of [**1**]Cl_2_-[**3**]Cl_2_ after purification. Gradient: 10-90% phase B/phase A, 15 min, detector UV channel=280 nm.


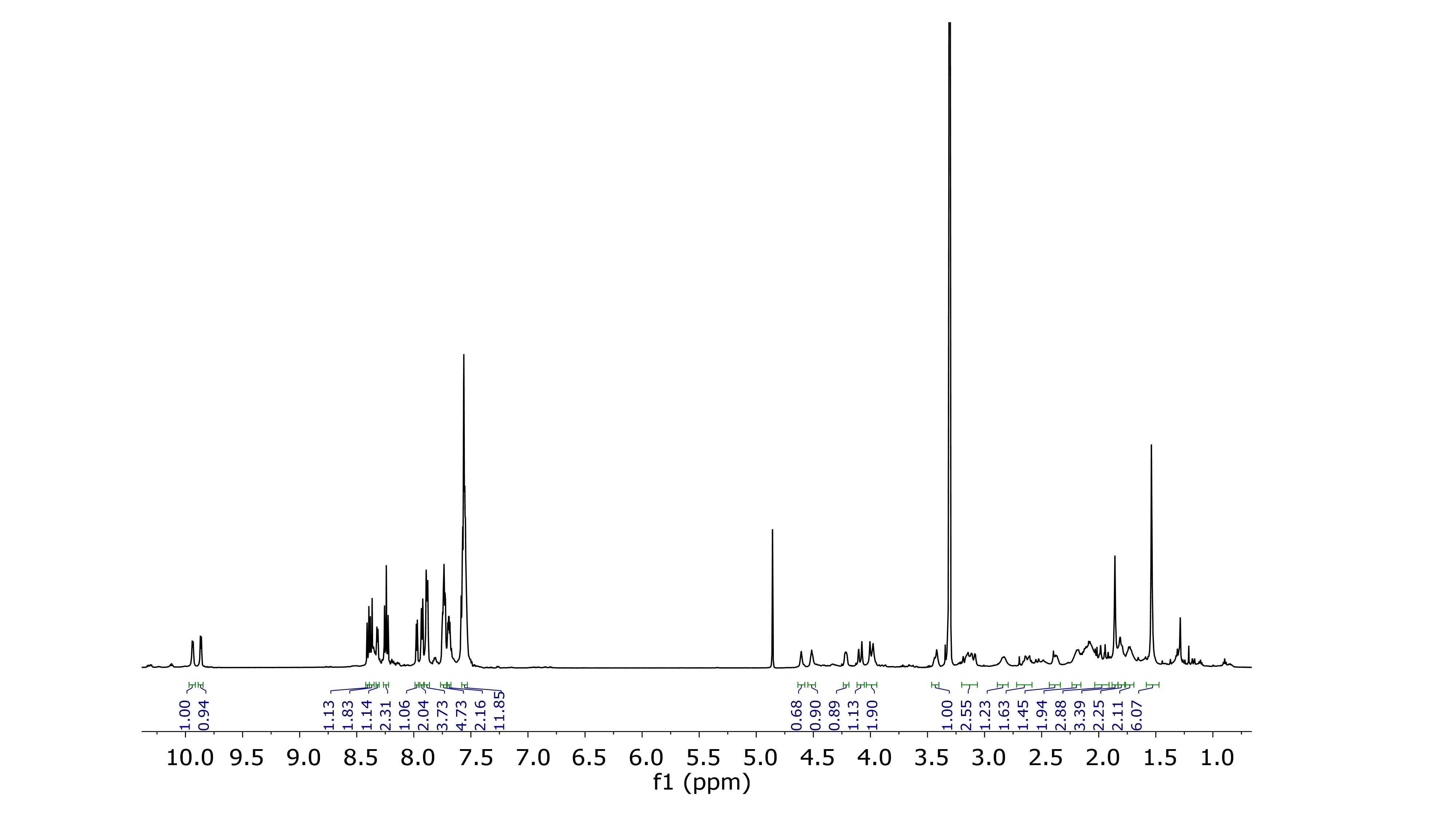


**Figure S5.** 600 MHz ^1^H NMR spectrum of **Δ**-[**1**]Cl_2_ in CD_3_OD at 293 K.


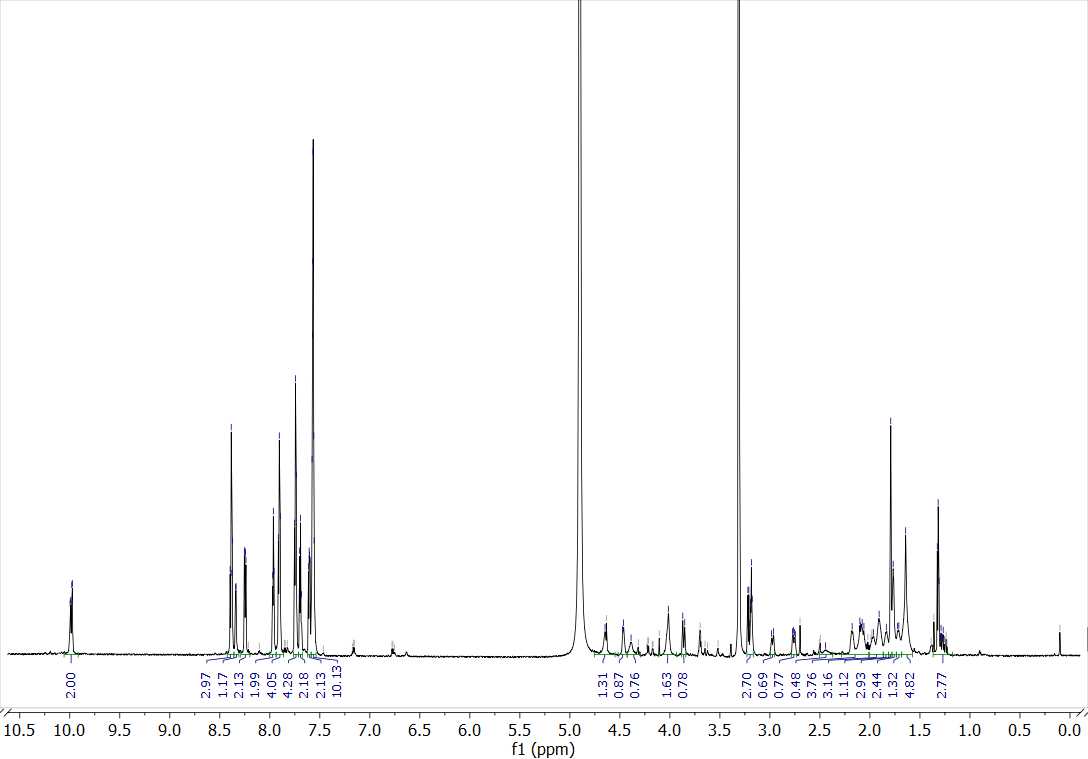


**Figure S6.** 850 MHz ^1^H NMR spectrum of Λ-[**1**]Cl_2_ in CD_3_OD at 293 K.


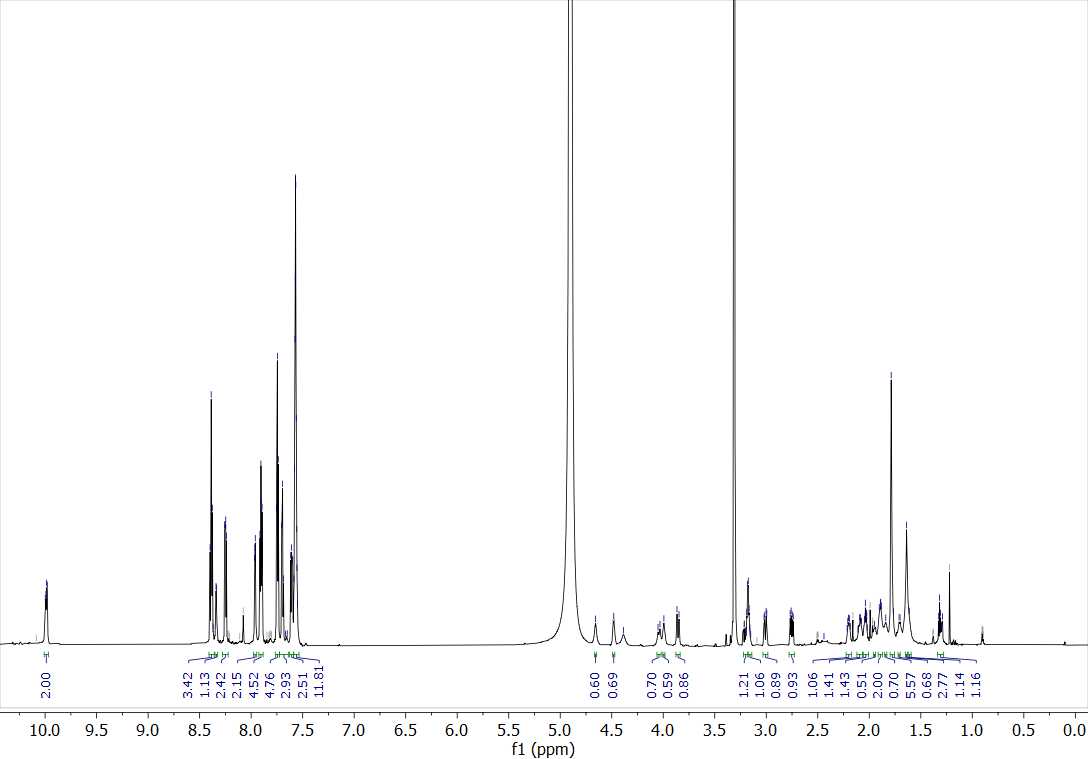


**Figure S7.** 850 MHz ^1^H NMR spectrum of Δ-[**2**]Cl_2_ in CD_3_OD at 293 K.


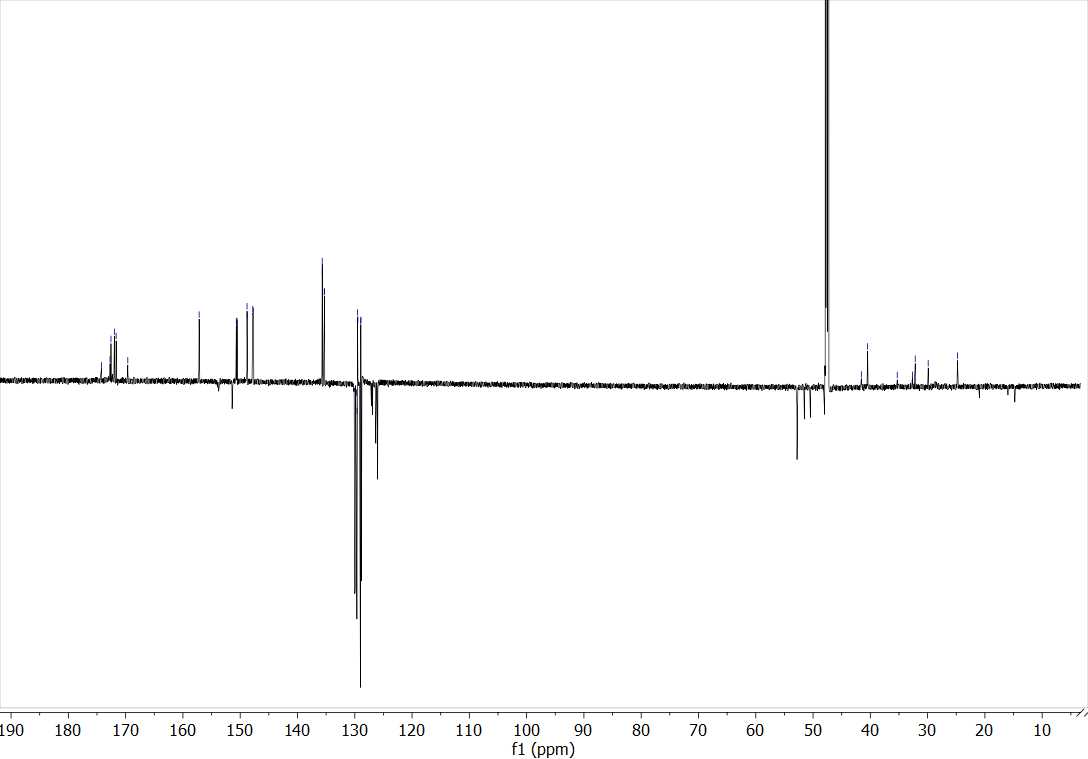


**Figure S8.** 214 MHz ^13^C-APT NMR spectrum of Δ-[**2**]Cl_2_ in CD_3_OD at 293 K.

**Figure S9.** 850 MHz ^1^H COSY NMR spectrum of Δ-[**2**]Cl_2_ in CD_3_OD at 293 K.

**Figure S10.** 850-214 MHz ^1^H-^13^C HSQC NMR spectrum of Δ-[**2**]Cl_2_ in CD_3_OD at 293 K.


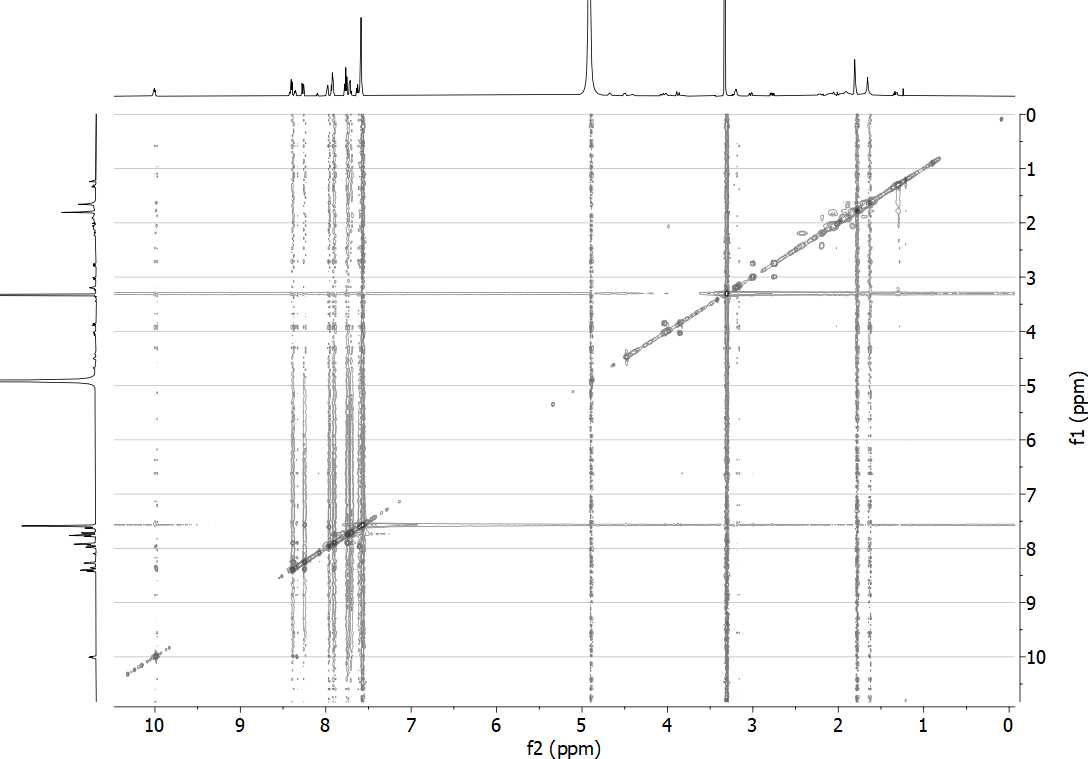


**Figure S11.** 850 MHz ^1^H NOESY NMR spectrum of Δ-[**2**]Cl_2_ in *CD_3_*OD at 293 K.


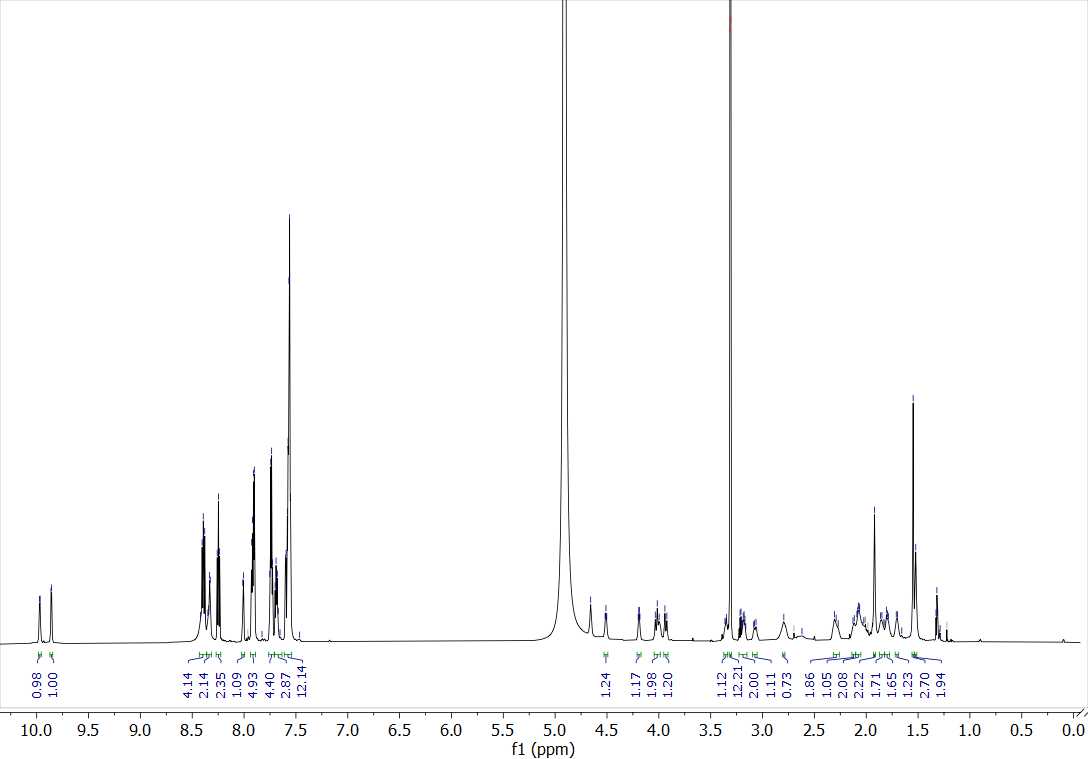


**Figure S12.** 850 MHz ^1^H NMR spectrum of Λ-[**2**]Cl_2_ in CD_3_OD at 293 K.


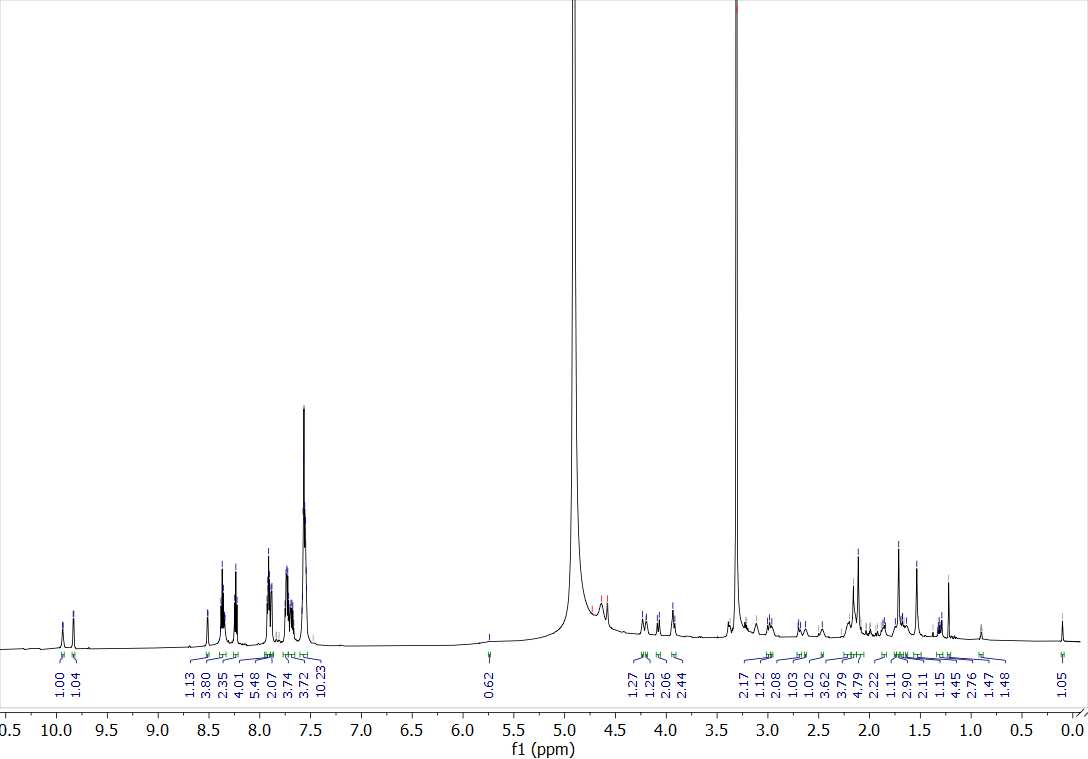
 **Figure S13.** 850 MHz ^1^H NMR spectrum of Δ-[**3**]Cl_2_ in CD_3_OD at 293 K.


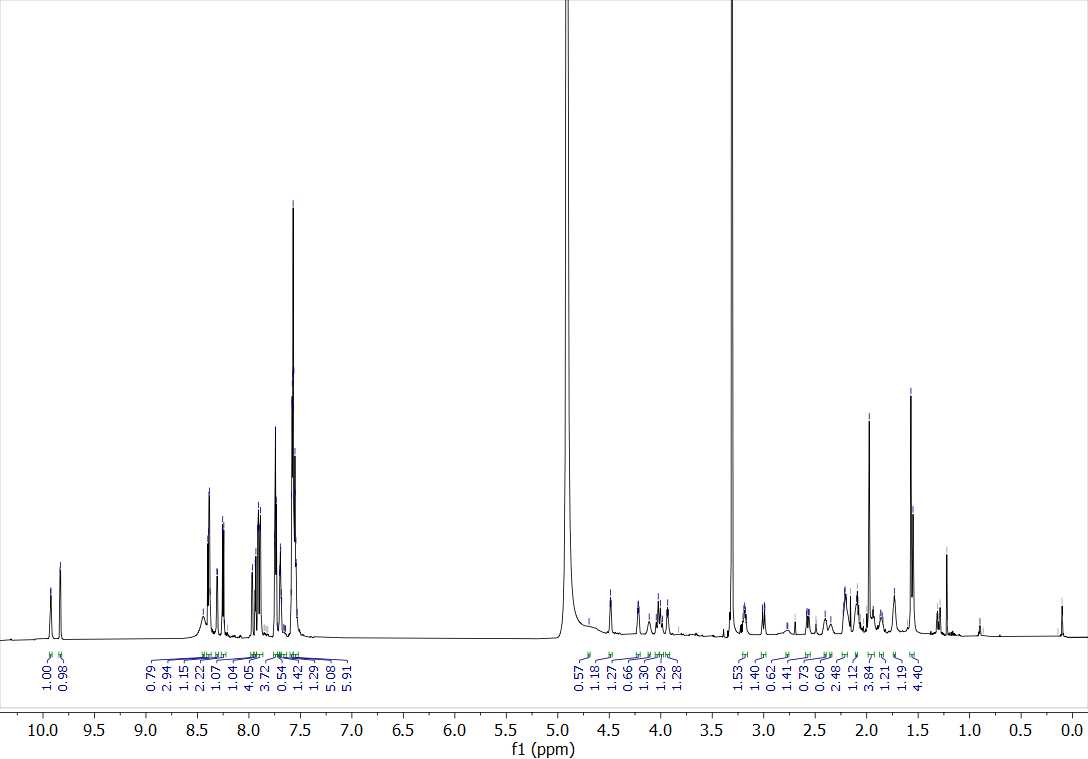
 **Figure S14.** 850 MHz ^1^H NMR spectrum of Λ-[**3**]Cl_2_ in CD_3_OD at 293 K.

**Table S1.** Retention times, wavelength of the main CD peaks, and integral HPLC area ratio of Λ/Δ isomer in [**1**]Cl_2_, [**1**]Cl_2_ and [**3**]Cl_2_ (amount of Δ-isomer is defined to be 1).

| **Complex** | **R_T_ (min)** | **Main Band in CD** | **Ratio to corresponding Δ-isomer** |
| --- | --- | --- | --- |
| Δ-[**1**]Cl_2_ | 10.6 | +274 nm **/** -287 nm | 1 |
| Λ-[**1**]Cl_2_ | 9.96 | -274 nm / +287 nm | 1 |
| Δ-[**2**]Cl_2_ | 10.0 | +274 nm / -287 nm | 1 |
| Λ-[**2**]Cl_2_ | 10.6 | -274 nm / +287 nm | 1.1 |
| Δ-[**3**]Cl_2_ | 10.2 | +274 nm / -287 nm | 1 |
| Λ-[**3**]Cl_2_ | 11.4 | -274 nm / +287 nm | 2 |


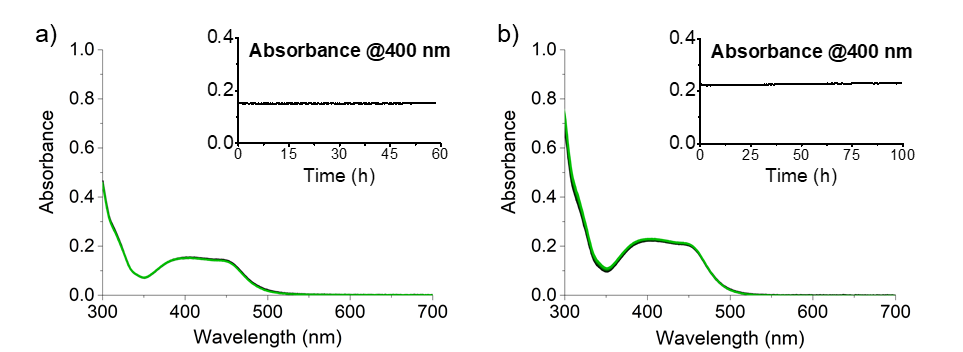


**Figure S15.** Time evolution of the UV-vis spectrum of Δ-[**2**]Cl_2_ under dark conditions in H_2_O (a, 20 µM) and Opti-MEM medium (b, 30 µM) at 298 K. The inserts show the time evolution of the absorbance at 400 nm.


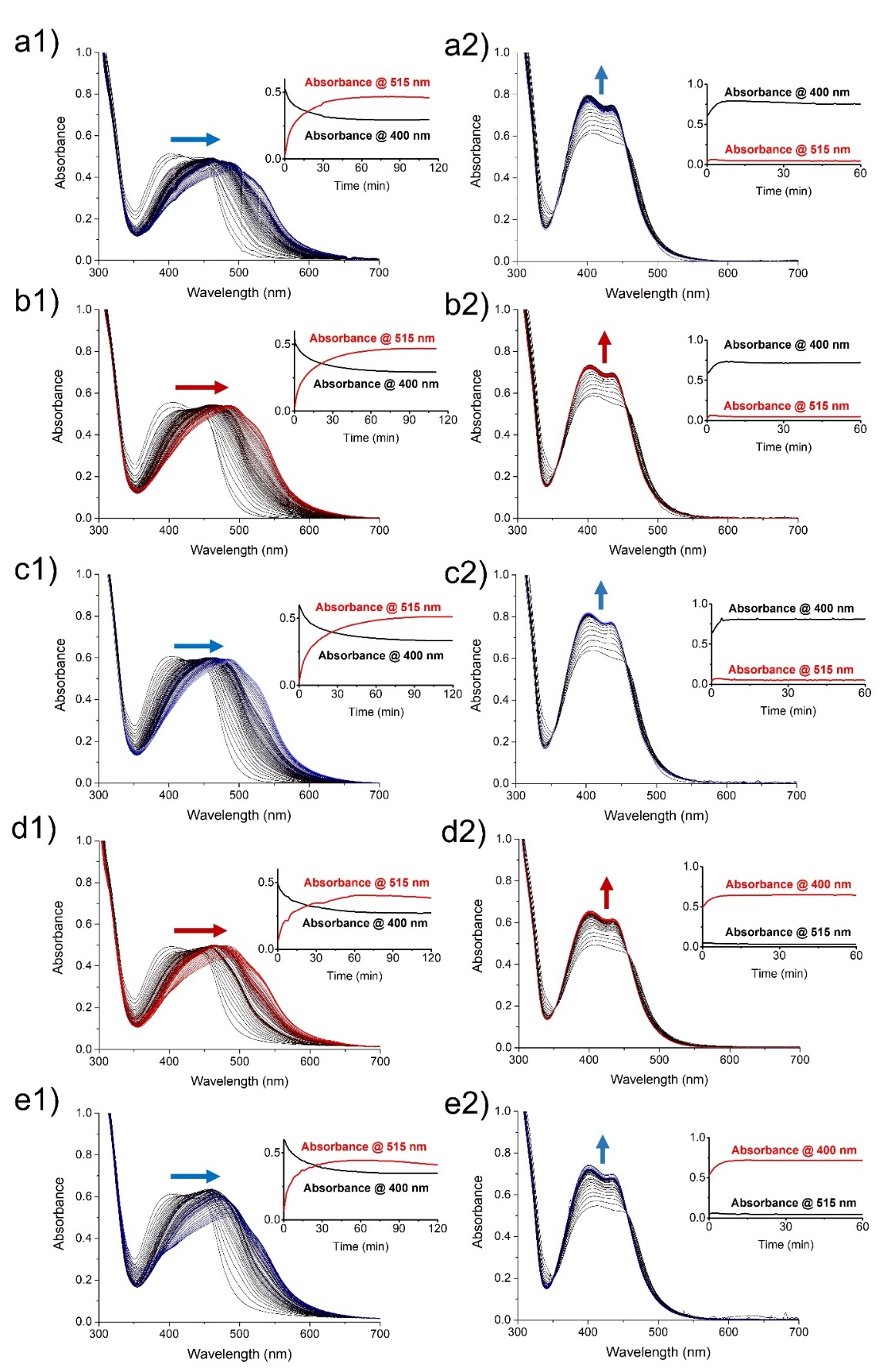


**Figure S16.** Time-dependence of the UV-vis spectra of Λ-[**1**]Cl_2_ (a), Δ-[**2**]Cl_2_ (b), Λ-[**2**]Cl_2_ (c), Δ-[**3**]Cl_2_ (d), Λ-[**3**]Cl_2_ (e) in H_2_O (1) or in H_2_O:CH_3_CN (1:1 v/v) (2) upon green light irradiation (4.1 mW/cm^2^, 515 nm). Y-axis shows absorbance and the evolution of the absorption spectrum is indicated by arrows. The inserts show the absorbance at 400 and 515 nm vs. irradiation time.


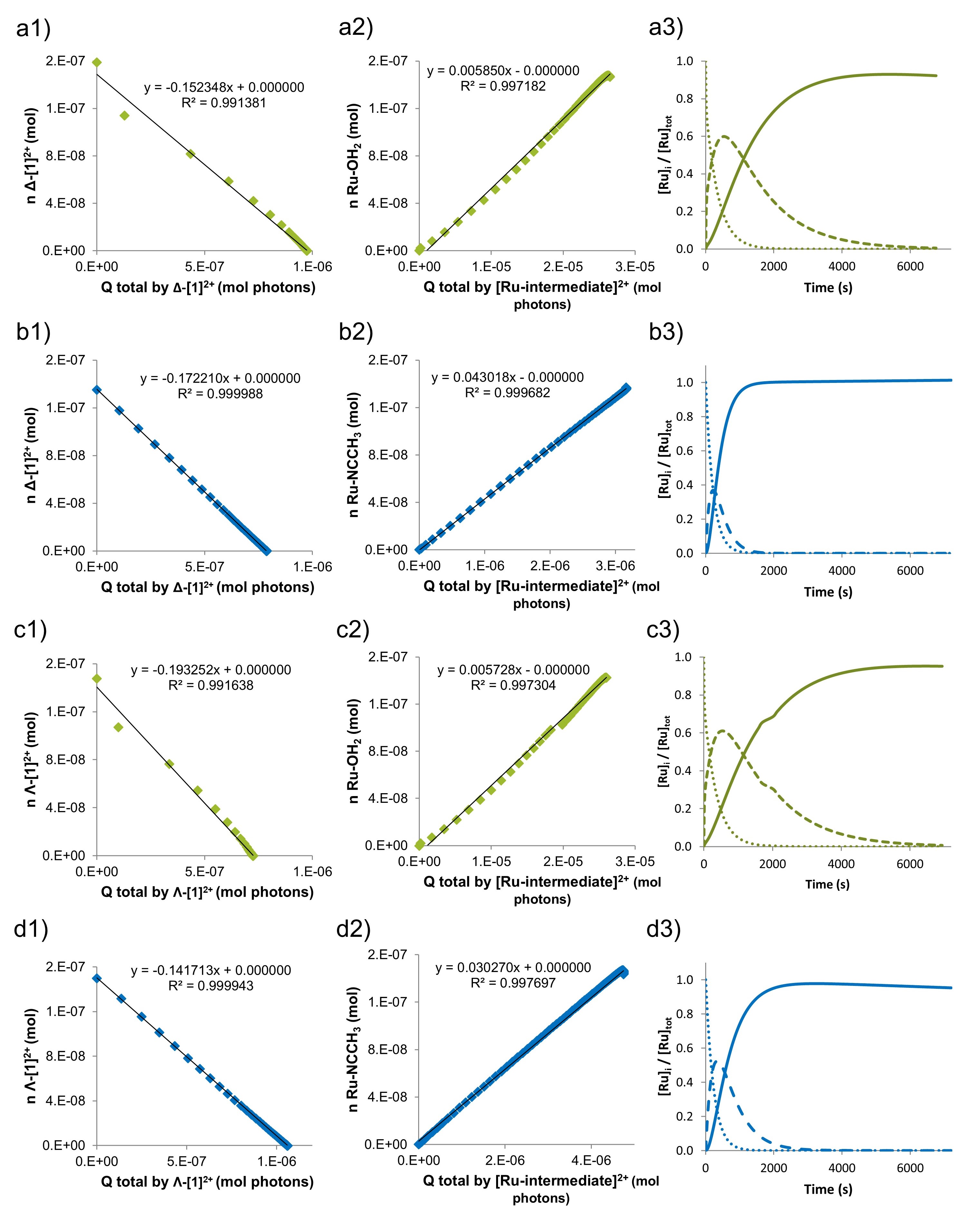


**Figure S17.** Fitting of the photosubstitution UV-vis data of Δ-[**1**]^2+^ in H_2_O (a1-a3), Δ-[**1**]^2+^ in H_2_O:CH_3_CN (1:1 v/v) (b1-b3), Λ-[**1**]^2+^ in H_2_O (c1-c3) and Λ-[**1**]^2+^ in H_2_O:CH_3_CN (1:1 v/v) (d1-d3). (1) Amount of [**1**]^2+^ plotted against the number of absorbed photons by the same species, both in mol. (2) Plot of the number of mol of η^1^ intermediate (substituted once either with H_2_O or with CH_3_CN) vs. the number of mol of absorbed photons since t=0. The absolute value of the slopes of each line equals the photosubstitution quantum yield for this step, **Φ_PS1_** or **Φ_PS2_**. (3) Evolution of the relative concentrations of reactant, intermediate and product according to Glotaran global fitting.


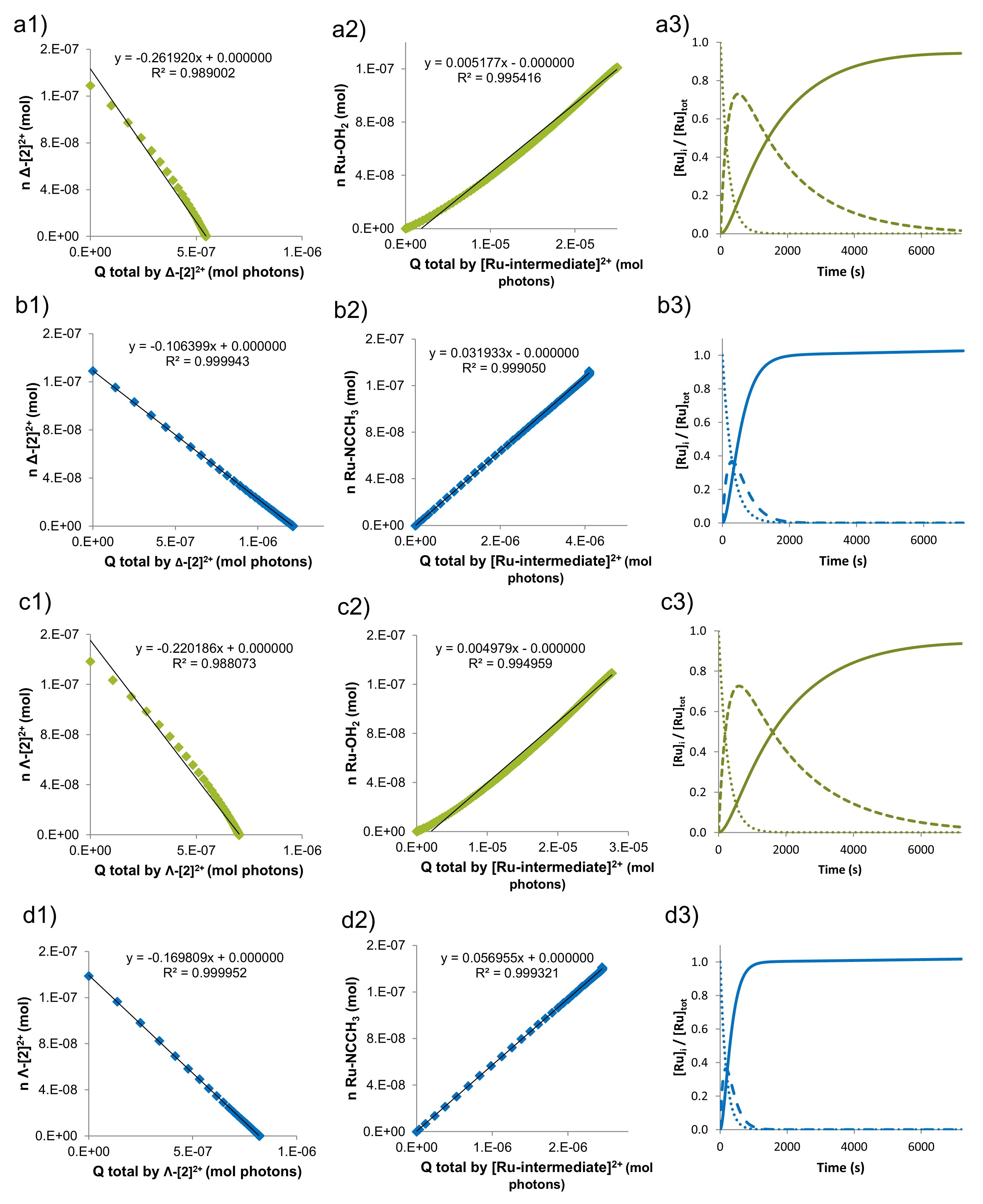


**Figure S18.** Fitting of the photosubstitution UV-vis data of Δ-[**2**]^2+^ in H_2_O (a1-a3), Δ-[**2**]^2+^ in H_2_O:CH_3_CN (1:1 v/v) (b1-b3), Λ-[**2**]^2+^ in H_2_O (c1-c3) and Λ-[**2**]^2+^ in H_2_O:CH_3_CN (1:1 v/v) (d1-d3). (1) Amount of [**2**]^2+^ plotted against the number of absorbed photons by the same species, both in mol. (2) Plot of the number of mol of η^1^ intermediate (substituted once either with H_2_O or with CH_3_CN) vs. the number of mol of absorbed photons since t=0. The absolute value of the slopes of each line equals the photosubstitution quantum yields for this step, **Φ_PS1_** or **Φ_PS2_**. (3) Evolution of the relative concentrations of reactant, intermediate and product according to Glotaran global fitting.


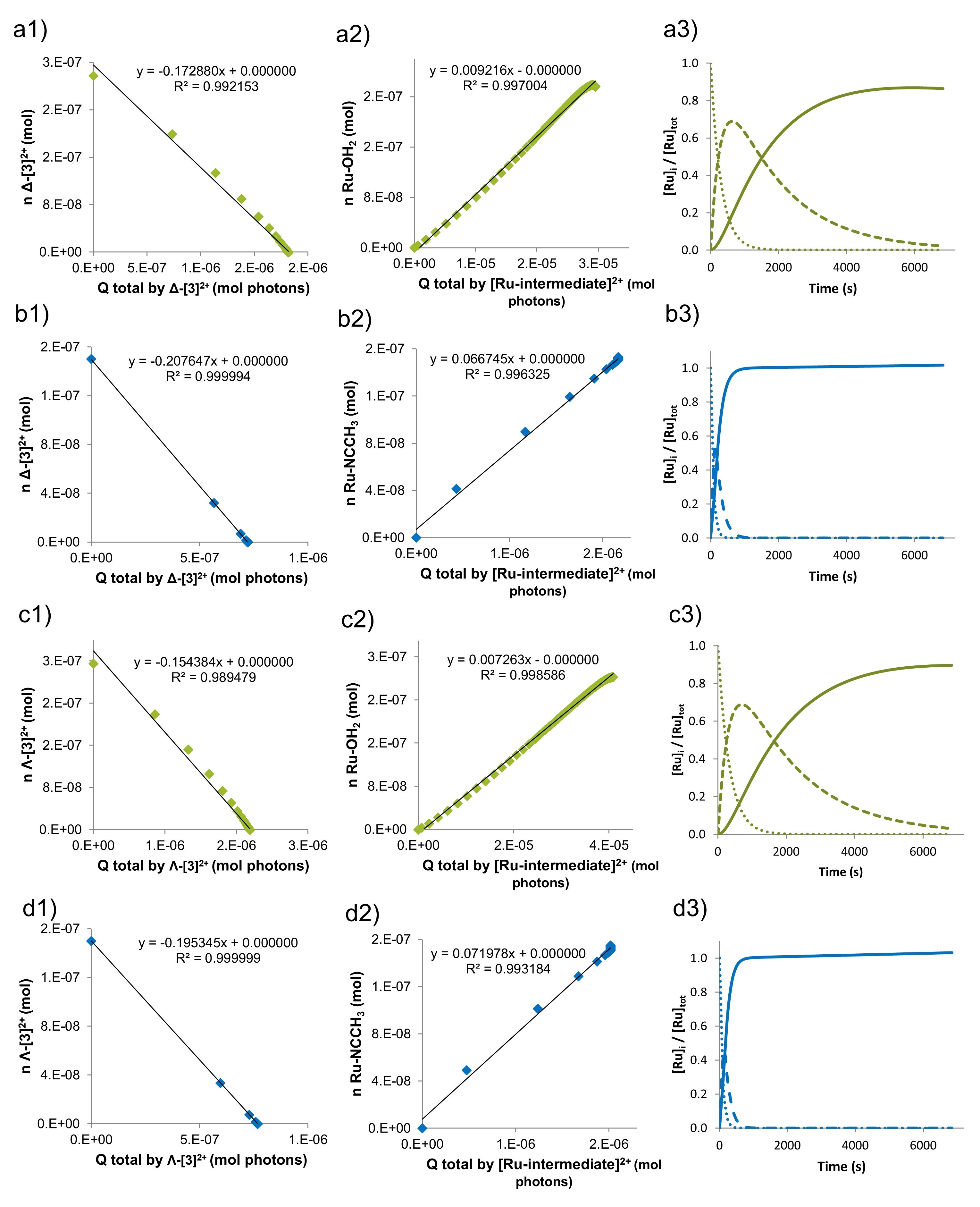


**Figure S19.** Fitting of the photosubstitution UV-vis data of Δ-[**3**]^2+^ in H_2_O (a1-a3), Δ-[**3**]^2+^ in H_2_O:CH_3_CN (1:1 v/v) (b1-b3), Λ-[**3**]^2+^ in H_2_O (c1-c3) and Λ-[**3**]^2+^ in H_2_O:CH_3_CN (1:1 v/v) (d1-d3). (1) Amount of [**3**]^2+^ plotted against the number of absorbed photons by the same species, both in mol. (2) Plot of the number of mol of η^1^ intermediate (substituted once either with H_2_O or with CH_3_CN) vs. the number of mol of absorbed photons since t=0. The absolute value of the slopes of each line equals the photosubstitution quantum yields for this step, **Φ_PS1_** or **Φ_PS2_**. (3) Evolution of the relative concentrations of reactant, intermediate and product according to Glotaran global fitting.


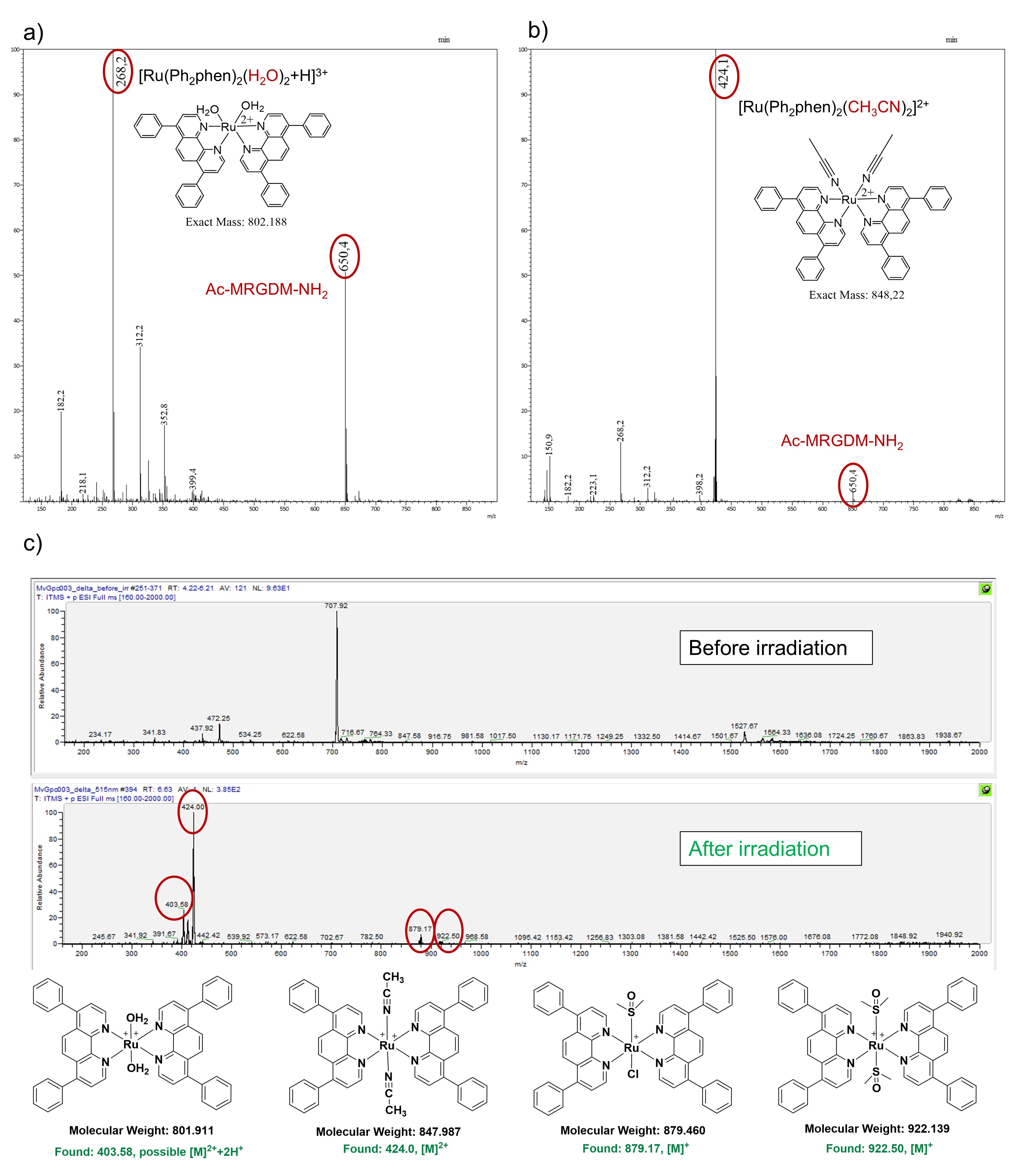


**Figure S20.** Mass spectra of a solution of Δ-[**1**]Cl_2_ irradiated with green light for 120 min in H_2_O (a) or in H_2_O:MeCN 1:1 v/v (b). The photosubstitution products Ac-MRGDM-NH_2_ was detected at 650.4 m/z (cacld m/z 650.27 [M + H]^+^), and Ru(Ph_2_phen)_2_(H_2_O)_2_]^2+^ was found at 268.2 m/z (calcd m/z 267.7: [M + H]^3+^), and [Ru(Ph_2_phen)_2_(CH_3_CN)_2_]^2+^ at m/z 424.1 (calcd m/z 424.1). (c) Mass spectra of Δ-[**1**]Cl_2_ in H_2_O with 0.5% DMSO before and after light irradiation, and the structure of the photoproducts that can be found in the traces.


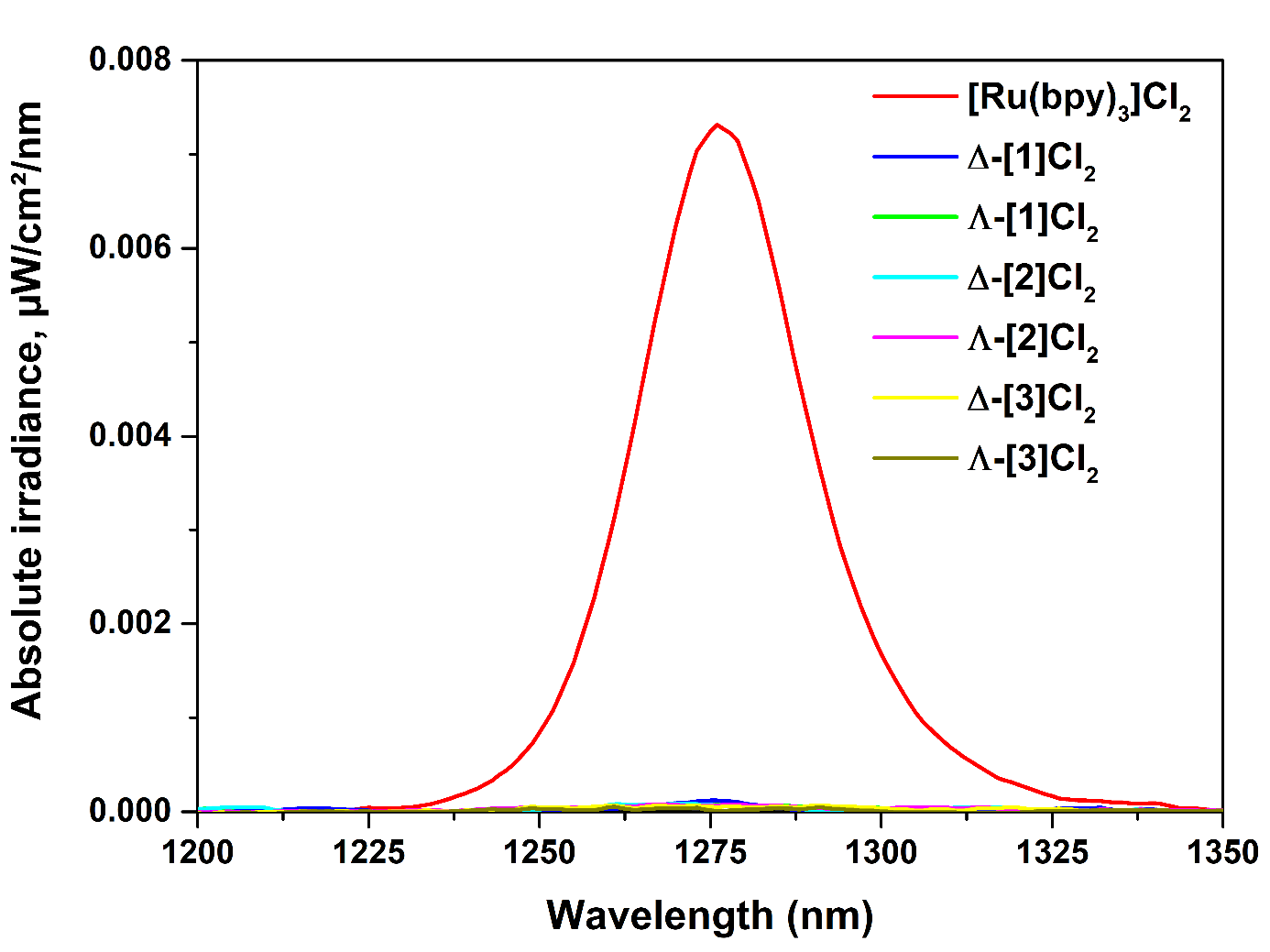


**Figure S21.** Normalized near infrared spectroscopy (NIR) emission from ^1^O_2_ generated by [**1**]Cl_2_-[**3**]Cl_2_ under blue light irradiation (450 nm) in CD_3_OD. [Ru(bpy)_3_]Cl_2_ was used as a reference compound, with Φ_Δ_ = 0.73 ± 0.12 in air-saturated CD_3_OD.


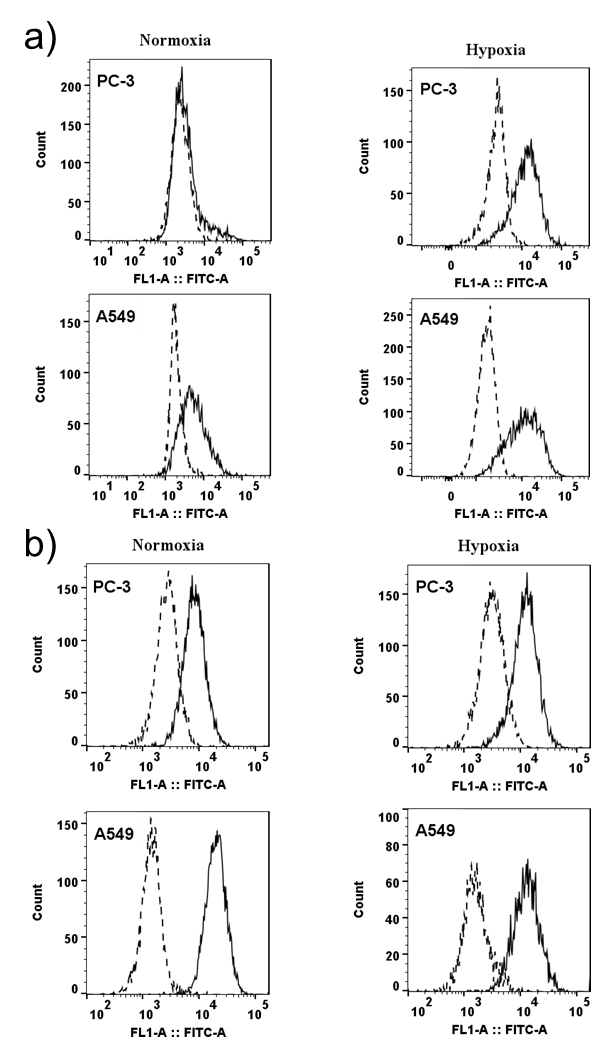


**Figure S22.** A) Representative flow cytometry histogram of integrin a_V_β_3_ (a) and integrin a_V_β_5_ (b) expression of PC-3 and A549 human cancer cell lines cultured either in normoxic (21% O_2_) or hypoxic (1% O_2_) condition. Solid lines represent the fluorescence intensity of the cells after incubation with anti-integrin a_V_β_3_ primary antibody, followed by Alexa Fluor™ 488 conjugated goat anti-mouse IgG secondary antibody. Dotted lines indicate the background signal obtained by staining with only the secondary antibody.


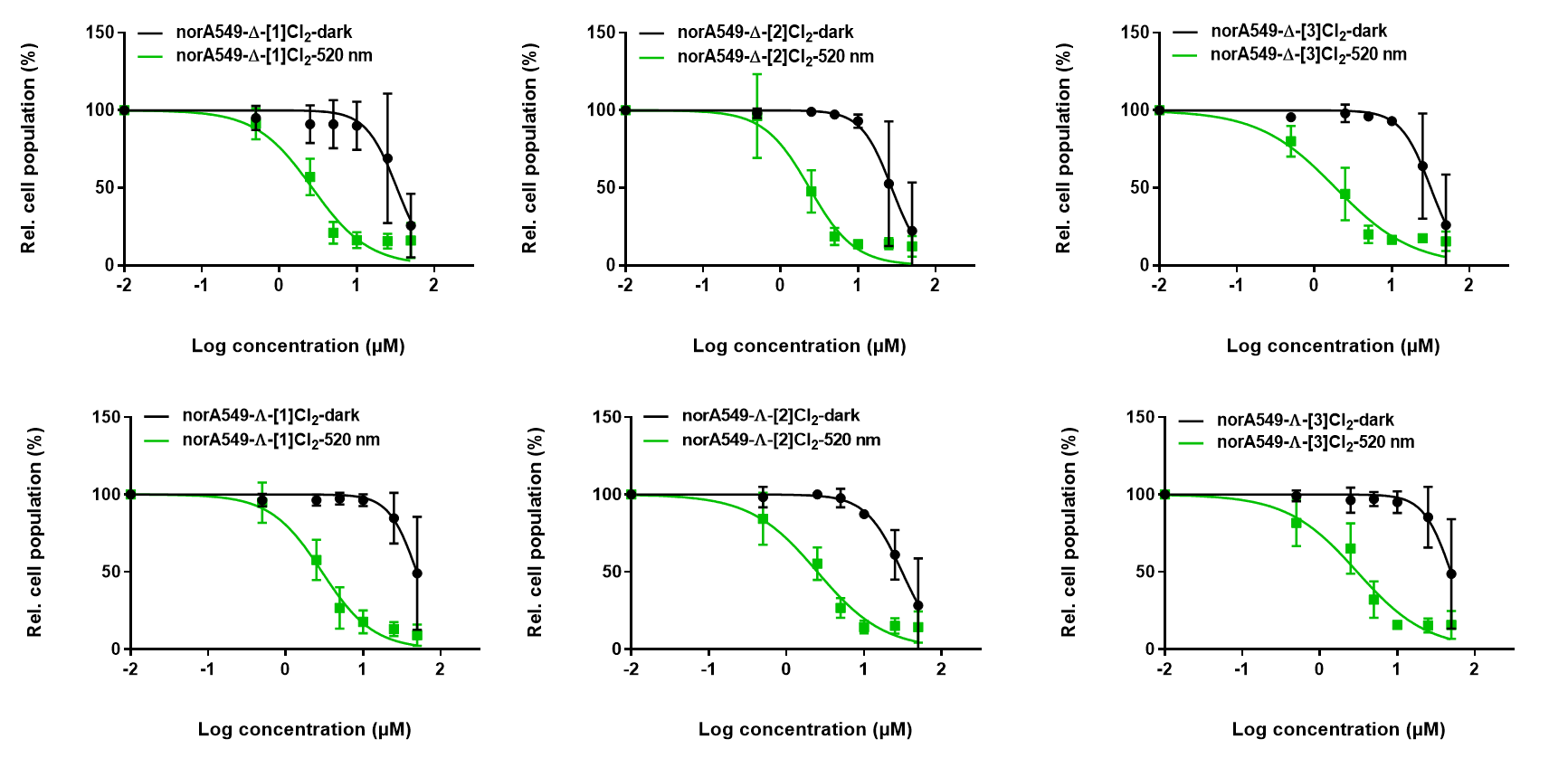


**Figure S23.** Dose-response curves in 2D monolayers of A549 human lung cancer cell lines after treatment with [**1**]Cl_2_–[**3**]Cl_2_ in normoxia (37°C, 21% O_2_ and 5% CO_2_) **without washing** before irradiation. Drug-to-light interval DLI = 24 h; black curve: dark condition; green curve: irradiated with green light (520 nm, 10.9 mW/cm^2^, 13.1 J/cm^2^, 20 min).


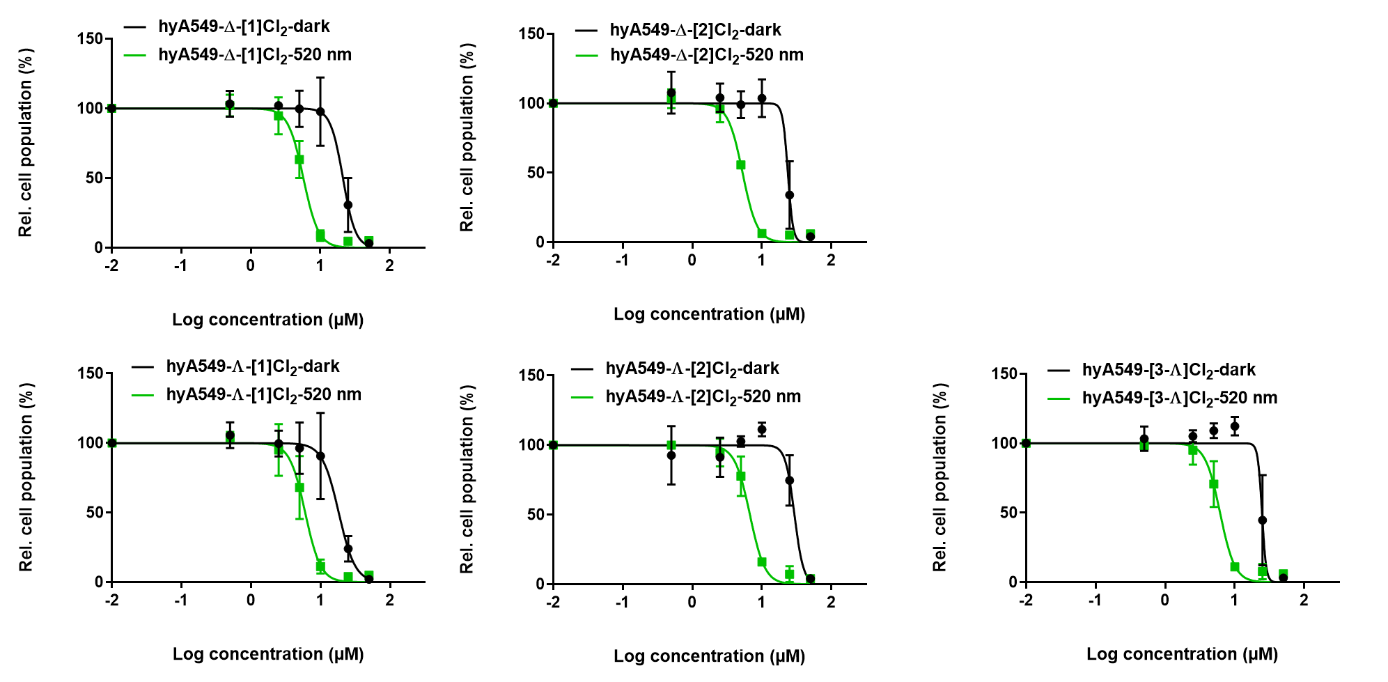


**Figure S24.** Dose-response curves in 2D monolayer of A549 human lung cancer cell lines after treatment with [**1**]Cl_2_–[**3**]Cl_2_ except Δ-[**3**]Cl_2_ in hypoxia (37°C, 1% O_2_ and 5% CO_2_) **without washing** before irradiation; DLI: 24 h; black curve: dark condition; green curve: irradiated with green light (520 nm, 7.22 mW/cm^2^, 13.1 J/cm^2^, 30 min).


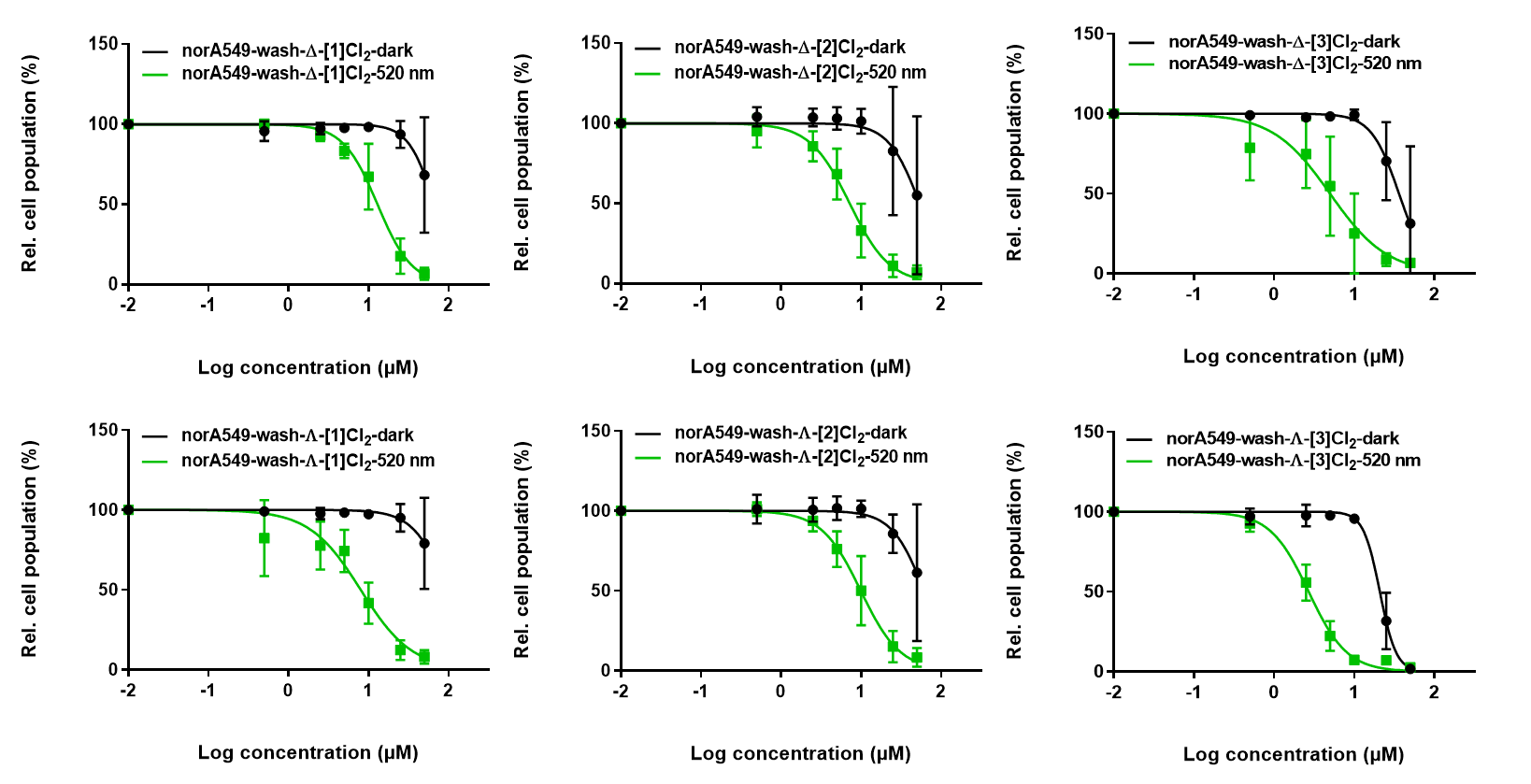


**Figure S25.** Dose-response curves in 2D monolayer of A549 human lung cancer cell lines after treatment with [**1**]Cl_2_–[**3**]Cl_2_ in normoxia (37°C, 21% O_2_ and 5% CO_2_) **with washing** (medium removal followed by addition of drug-free medium) before irradiation. DLI: 24 h; black curve: dark condition; green curve: irradiated with green light (520 nm, 10.9 mW/cm^2^, 13.1 J/cm^2^, 20 min).


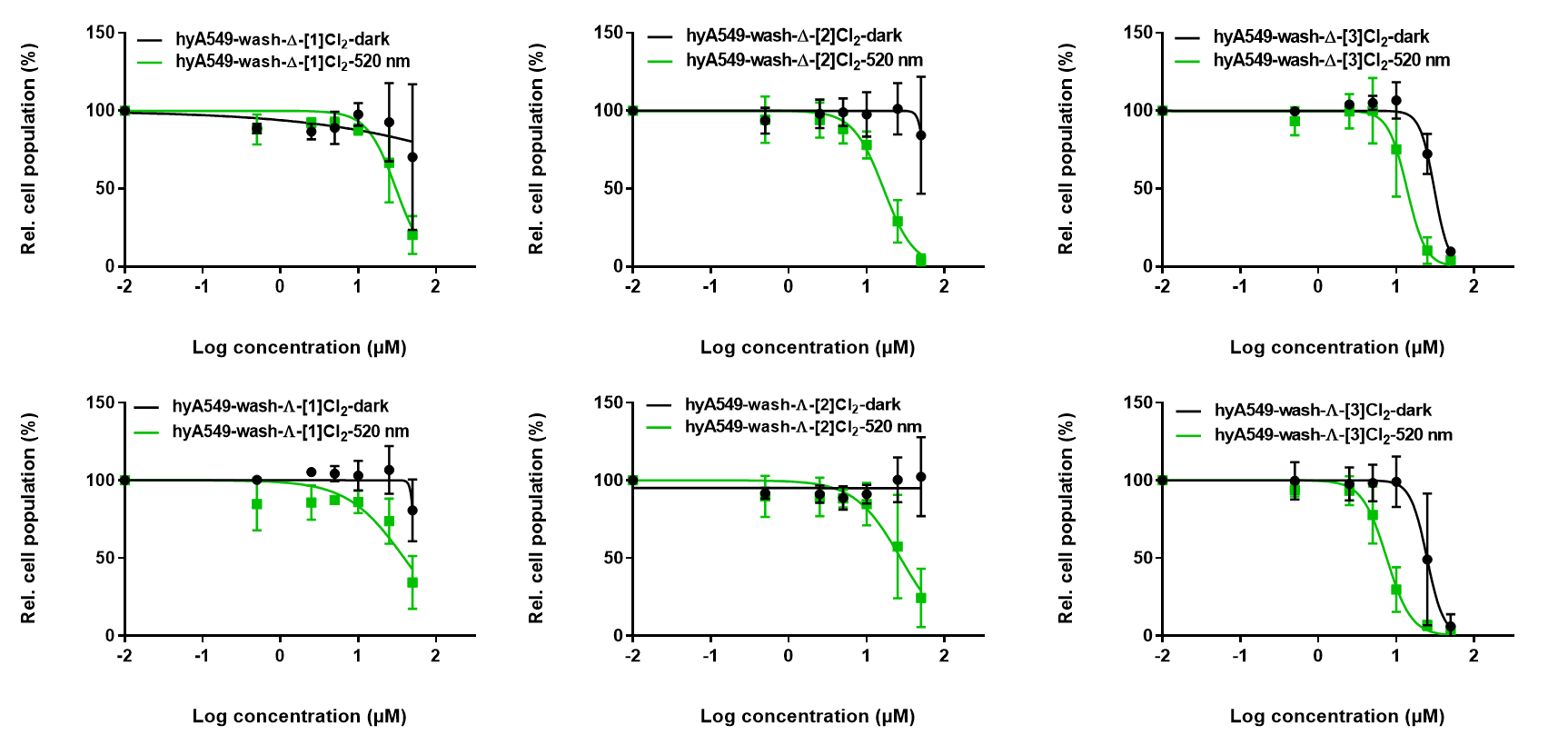


**Figure S26.** Dose-response curves in 2D monolayer of A549 human lung cancer cell lines after treatment with [**1**]Cl_2_–[**3**]Cl_2_ in hypoxia (37°C, 1% O_2_ and 5% CO_2_) **with washing** (medium removal followed by addition of drug-free medium) before irradiation; DLI = 24 h; black curve: dark condition; green curve: irradiated with green light (520 nm, 7.22 mW/cm^2^, 13.1 J/cm^2^, 30 min).


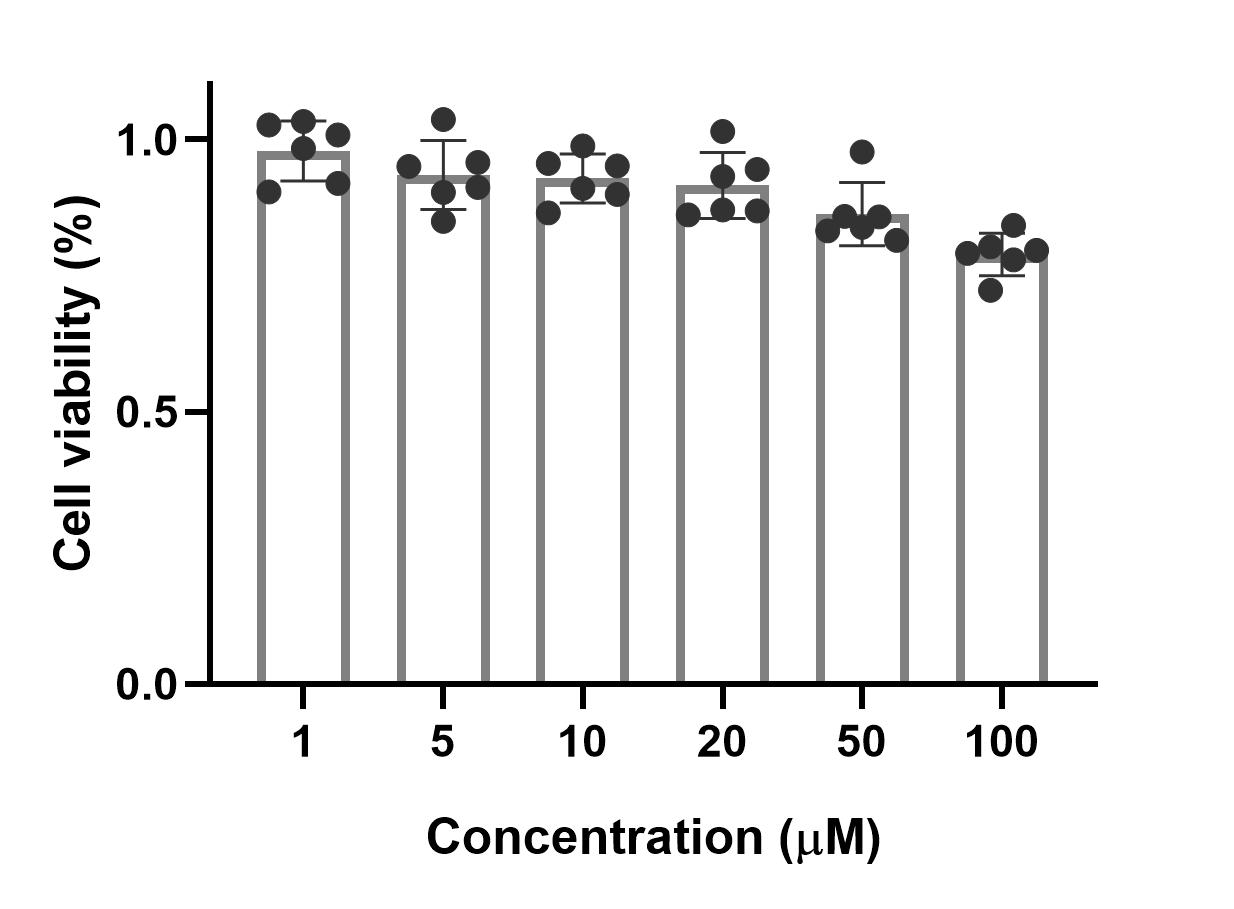


**Figure S27.** Cell viability of 2D monolayer of A549 cells after treated with [Ru(Ph_2_phen)_2_(H_2_O)_2_](PF_6_)_2_ in normoxia (37°C, 21% O_2_ and 5% CO_2_) for 72 h, without washing.


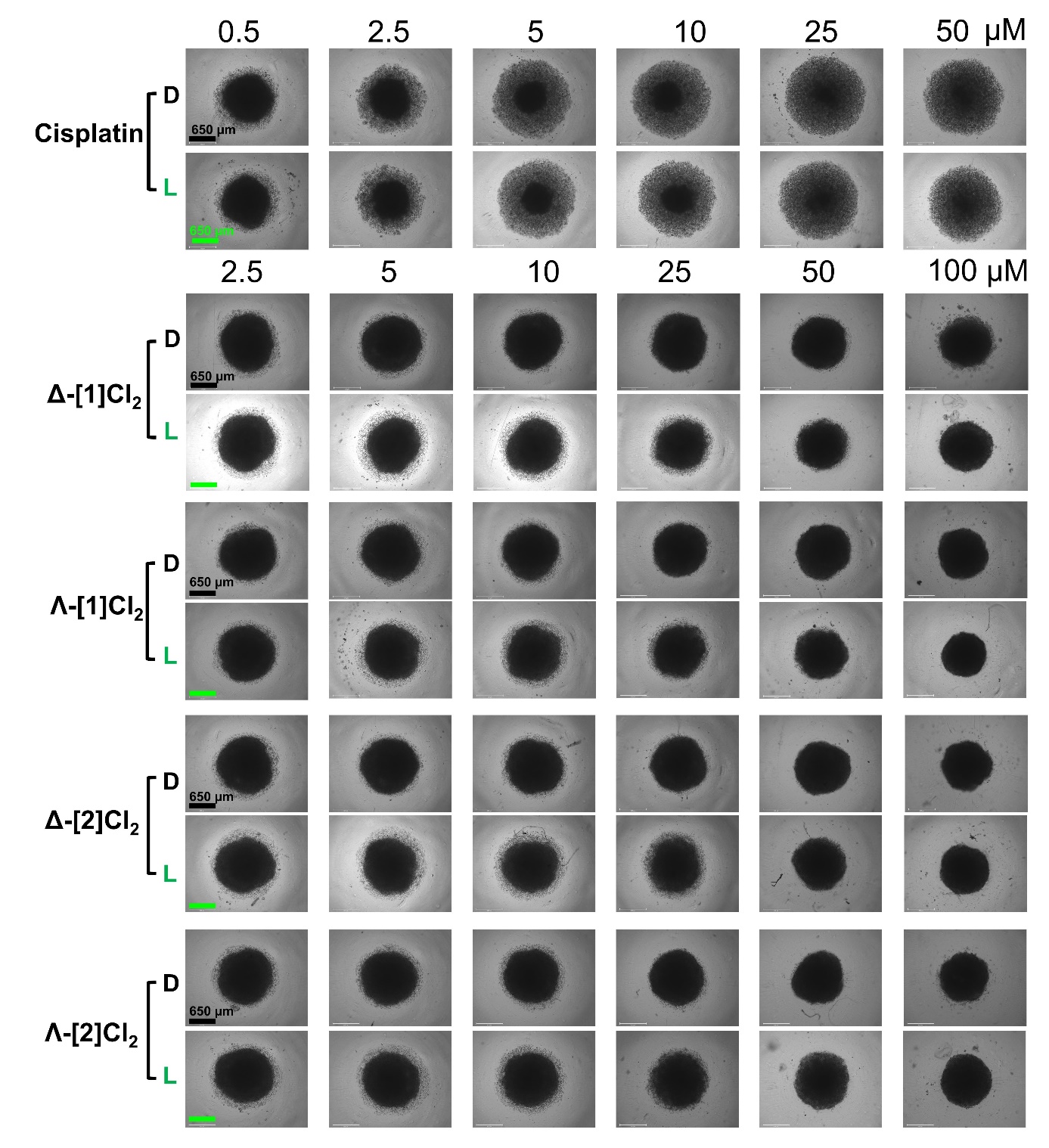


**Figure S28.** Bright field images of A549 3D tumor spheroids cultured under normoxia and treated with different concentrations of cisplatin or [**1**]Cl_2_–[**2**]Cl_2_, left in the dark or irradiated with green light (520 nm, 13.1 J/cm^2^). Scale bar = 650 µm.


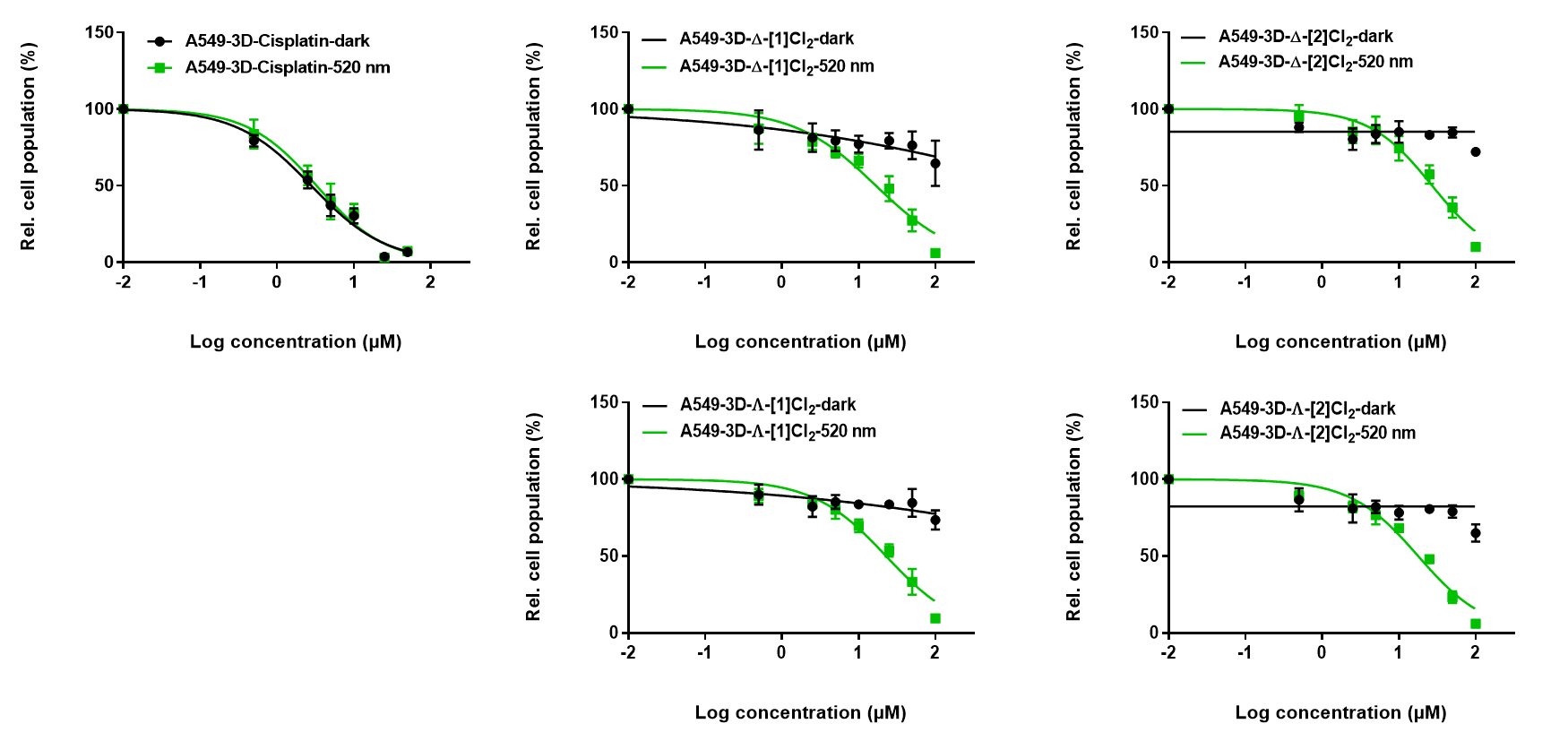


**Figure S29.** Dose-response curves for U87MG 3D tumor spheroids incubated in normoxic conditions with cisplatin or [**1**]Cl_2_–[**2**]Cl_2_ in the dark (in black) or irradiated with green light (in green, 520 nm, 13.1 J/cm^2^).


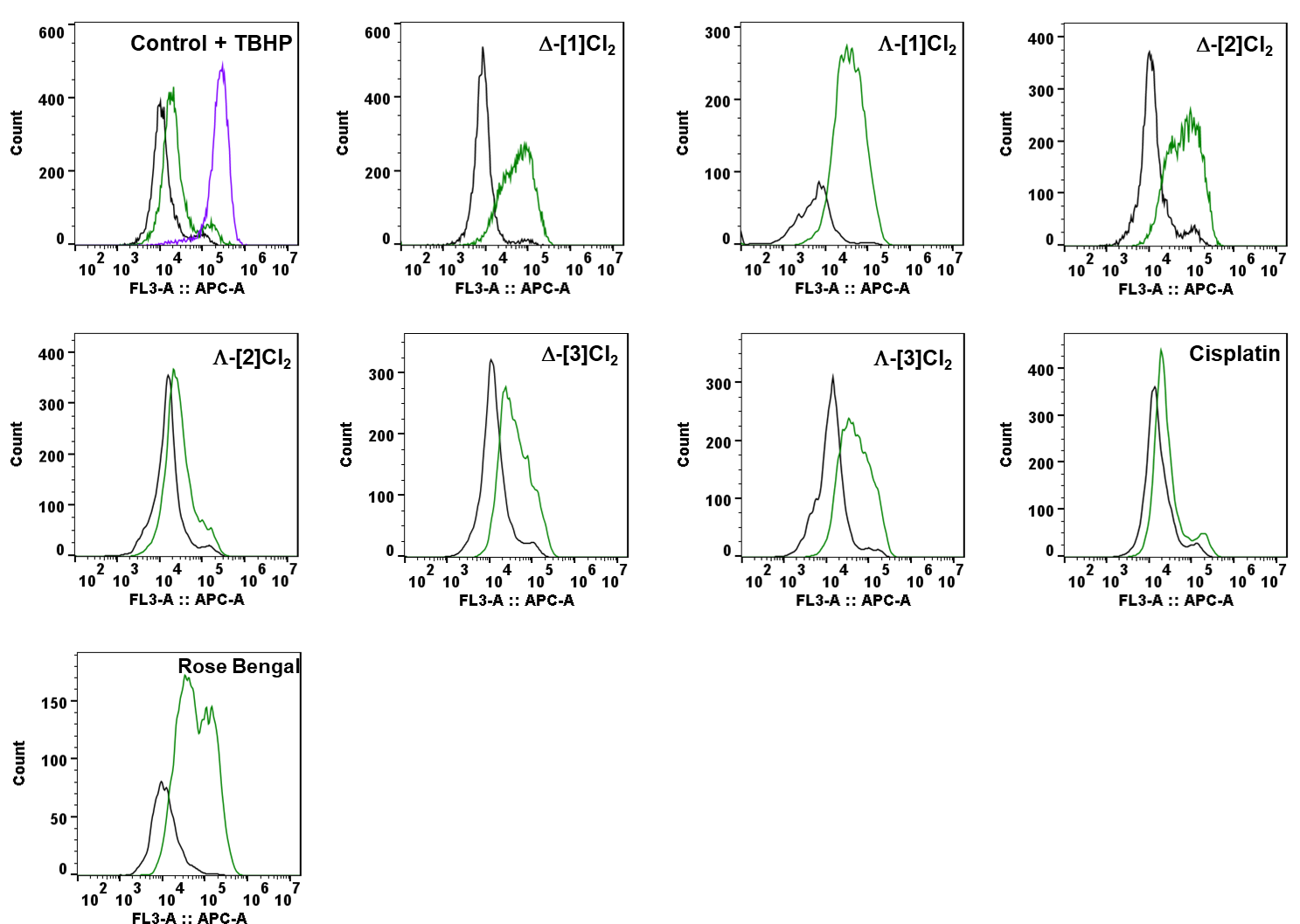


**Figure S30.** Reactive Oxygen Species generation in A549 cells under normoxia (21% O_2_) according to FACS analysis using CellROX™ Deep Red Reagent as non-selective ROS probe, after treatment with medium only (negative control), tBHP (250 μM, positive control), complexes [**1**]Cl_2_–[**3**]Cl_2_, cisplatin, or Rose Bengal (15 μM, 24 h) in the dark or after light irradiation (520 nm, 13.1 J/cm^2^). Dark group (black curve), light group (green curve) and tBHP (purple curve) samples are as shown. X-axis represents the ROS probe’s intensity detected by APC-A channel of FACS, higher value means higher ROS generation and Y-axis represents counted cell numbers.


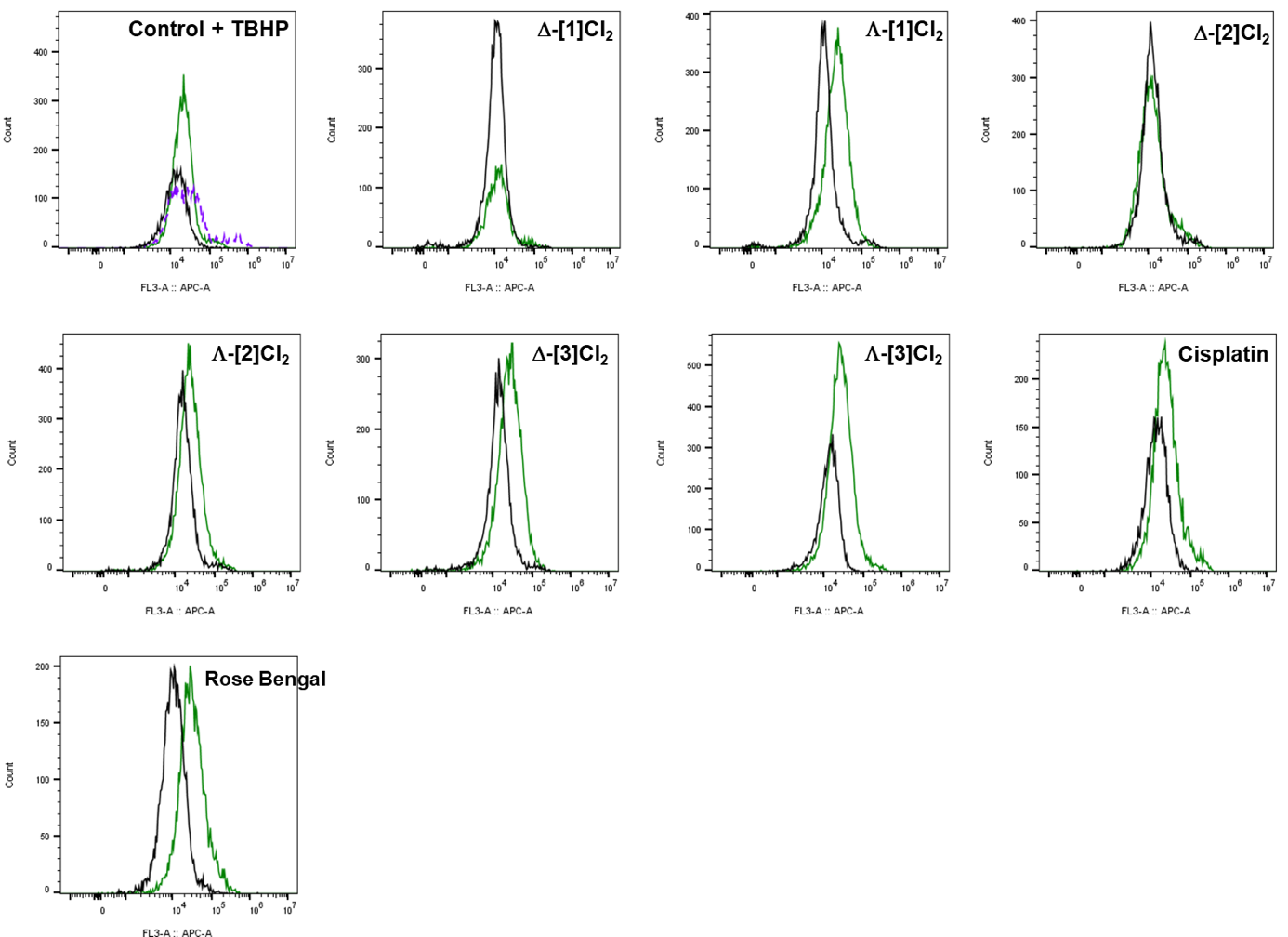


**Figure S31.** Reactive Oxygen Species generation in A549 cells under hypoxia (1% O_2_) according to FACS analysis using CellROX™ Deep Red Reagent as non-selective ROS probe, after treatment with medium only (negative control), tBHP (250 μM, positive control), complexes [**1**]Cl_2_–[**3**]Cl_2_, cisplatin, or Rose Bengal (15 μM, 24 h) in the dark or after light irradiation (520 nm, 13.1 J/cm^2^). Dark group (black curve), light group (green curve) and tBHP (purple curve) samples are as shown. X-axis represents the ROS probe’s intensity detected by APC-A channel of FACS, higher value means higher ROS generation and Y-axis represents counted cell numbers.


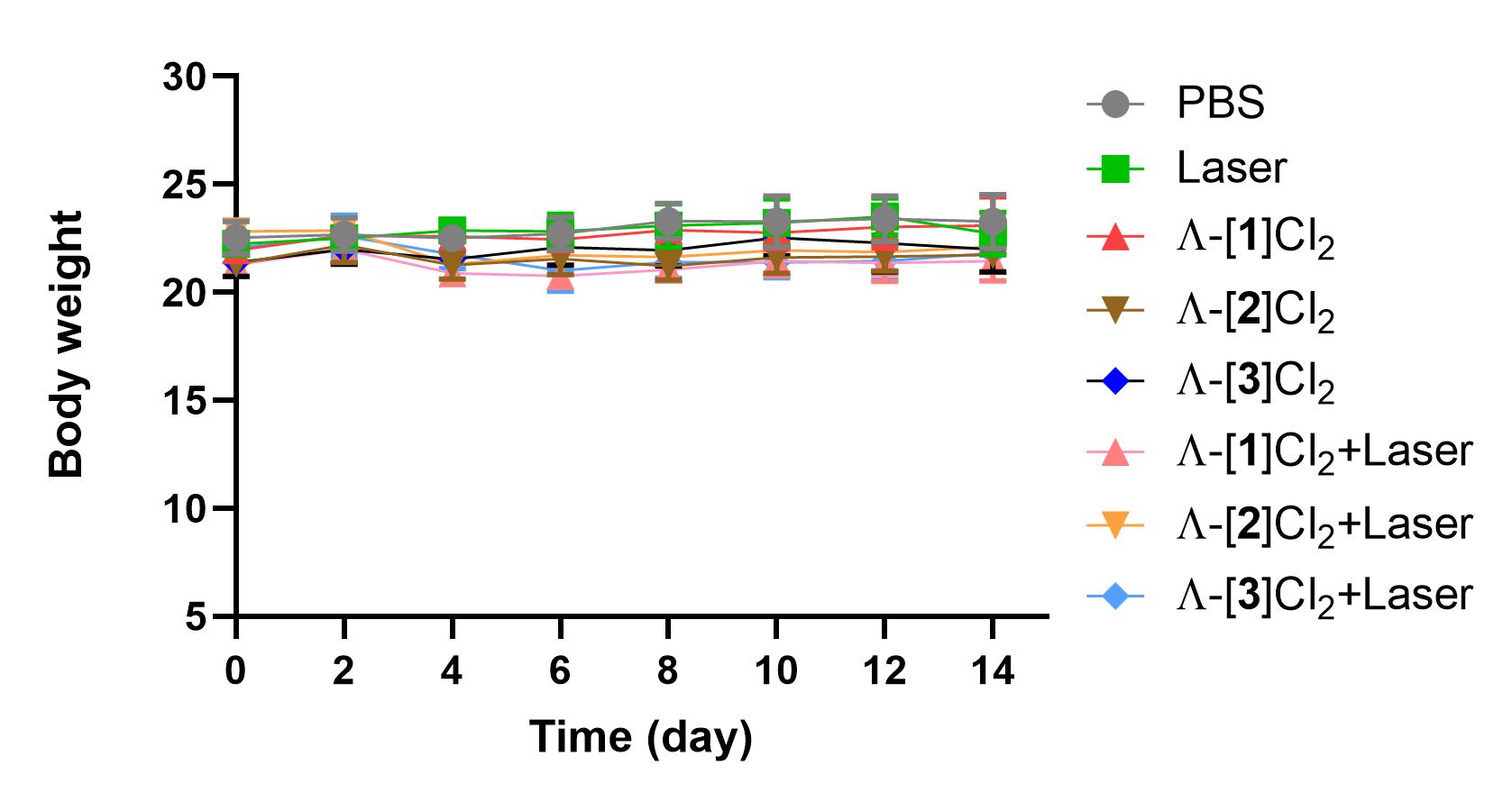


**Figure S32.** Body weight of mice following time evolution after treatments for 14 days, n=5.


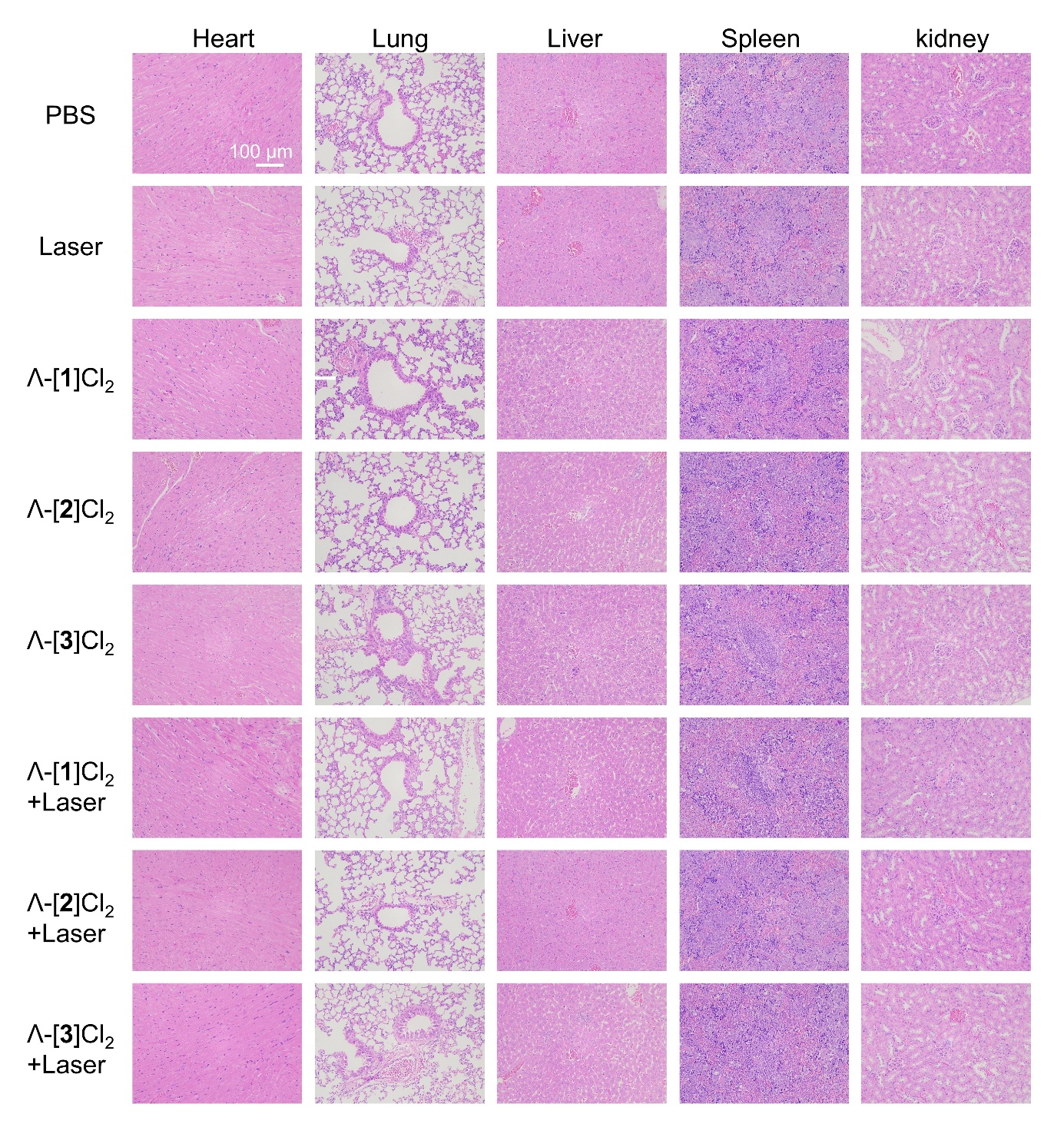


**Figure S33.** H&E stained images of major organs resected from of A549 tumor-bearing mice after different treatments at day 14.
